# Immunogenicity and safety of seasonal influenza vaccine co-administered with other vaccines: a systematic review and meta-analysis

**DOI:** 10.1038/s41541-026-01548-z

**Published:** 2026-09-02

**Authors:** Samuel Asri, Paraskevi C. Fragkou, Elena Ahrens, Svenja S. Hartmann, Clemens Thölken, Chrysanthi Skevaki

**Affiliations:** 1 Institute of Laboratory Medicine, Universities of Giessen and Marburg Lung Centre (UGMLC), Philipps University Marburg, German Centre for Lung Research (DZL), Marburg, Germany; 2 Internal Medicine Department, Aigli Medical Clinic, Athens, Greece; 3 Centre for Research and Education in Public Health (CEPHRE), Academy of Athens, Athens, Greece; 4 Department of Anesthesia, Critical Care and Pain Medicine, Beth Israel Deaconess Medical Center, Harvard Medical School, Boston, MA USA; 5 Center for Anesthesia Research Excellence (CARE), Beth Israel Deaconess Medical Center, Boston, MA USA; 6 Center for Synthetic Microbiology (SYNMIKRO), Philipps University Marburg, Marburg, Germany; 7 Institute of Clinical Chemistry and Laboratory Medicine, Institute of Virology and Research Center for Immunotherapy (FZI), University Medical Center of the Johannes Gutenberg University Mainz, Mainz, Germany; 8 Department of Environmental Health, Harvard T.H. Chan School of Public Health, Boston, MA USA

**Keywords:** Diseases, Health care, Immunology, Medical research, Microbiology

## Abstract

Seasonal influenza remains a leading cause of global morbidity and mortality, highlighting the need for vaccination strategies that improve coverage and streamline vaccine delivery. In this systematic review and meta-analysis, we searched PubMed, Embase, Web of Science, Scopus, and the Cochrane Central Register of Controlled Trials for randomised controlled trials (RCTs), cohort, case-control, and cross-sectional studies, evaluating immunogenicity and safety of same-day co-administration of influenza vaccines with COVID-19 or other vaccines, compared with non-concomitant administration. Comparators included sequential administration, single vaccine administration or placebo-controlled delayed vaccination. Risk of bias was evaluated using the Cochrane Risk-of-Bias tool for Randomized Trials and Risk of Bias In Non-randomized Studies of Interventions; certainty of evidence was evaluated using Grading of Recommendations, Assessment, Development and Evaluation. Immunogenicity was assessed using geometric mean fold rise (GMFR) in antibody titres and seroprotection rate. Safety was assessed by adverse event (AE) incidence. 52 eligible studies were included. Influenza immunogenicity was comparable between the co-administration and non-concomitant comparator group across all strains (H1N1 GMFR ratio of means (ROM): 1.02 [95% CI: 0.95–1.10]; H3N2, 1.05 [95% CI: 0.97–1.13]; B strain, 1.01 [95% CI: 0.97–1.05]). Pooled risk ratio (RR) for seroprotection was 1.00 for all three strains with 95% CIs ranging from 0.99–1.01. GMFR for COVID-19 vaccines was modestly reduced under co-administration (ROM 0.84 [95% CI: 0.74–0.95]; *p* = 0.006). Serious AEs were more frequent in the co-administration group compared to the non-concomitant group (RR 1.41 [95% CI: 1.07–1.86]; *p* = 0.014; absolute risk difference: 1.56 percentage points). Overall, co-administration preserves influenza immunogenicity but modestly reduces COVID-19 vaccine GMFR. Although safety findings warrant cautious interpretation, the low absolute risk difference supports its feasibility as a strategy to streamline vaccination schedules and improve uptake.

**Figure:**
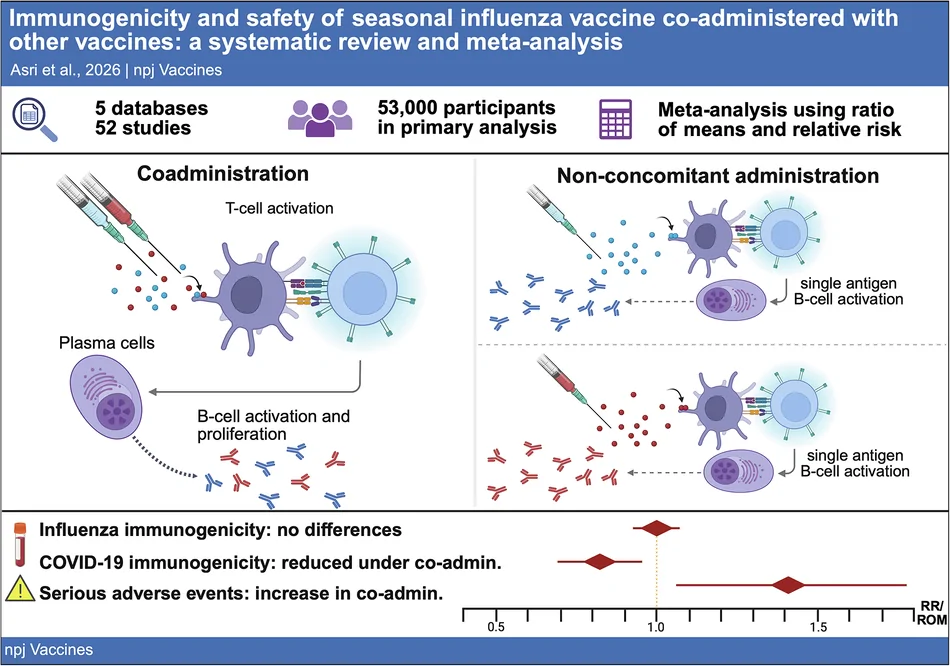

## Introduction

Seasonal influenza remains a substantial global health concern, with an estimated incidence exceeding one billion cases and approximately 350,000 deaths annually^1,2^. It currently ranks as the third leading global cause of mortality attributable to lower respiratory tract infections (LRTI)^2^. Following a temporary decline, influenza incidence has resurged significantly following the Coronavirus Disease 2019 (COVID-19) pandemic^3,4^. In the United States, approximately 8% of all outpatient medical visits are now attributable to influenza-like illness, a proportion not seen since the 2009 H1N1 pandemic^5^.

Given the proven effectiveness of vaccination in reducing morbidity and mortality, maximising vaccination coverage is essential to mitigate the clinical and economic burden of seasonal influenza^6,7^. This need is particularly important during overlapping respiratory virus seasons, when influenza, COVID-19, and other respiratory viruses co-circulate and increase pressure on healthcare systems^8^. High risk factors for severe outcomes among different respiratory pathogens significantly overlap^9^. However, adult vaccination coverage remains persistently suboptimal^10^, highlighting the need for strategies that reduce missed opportunities and improve vaccine uptake, including co-administration in pharmacy and primary care settings^11^. Non-concomitant vaccination poses considerable logistical and financial challenges, particularly when implemented at scale and during overlapping respiratory virus seasons^12^. Co-administration of influenza and COVID-19 vaccines in a single clinical encounter has emerged as a pragmatic strategy to improve uptake and reduce strain on healthcare infrastructure based on evidence concerning safety and immunogenicity of co-administration^13,14^. This approach is currently recommended by the World Health Organization (WHO)^15^, the US Centers for Disease Control and Prevention (CDC) and other public health authorities^16,17^. Despite these recommendations, real-world uptake remains inconsistent, with considerable variability in implementation practices. Recent findings emphasise the limited availability of robust, high-quality evidence from real-world settings confirming the safety and immunogenicity of co-administration, as well as the potential advantages of non-concomitant administration^17,18^.

We therefore conducted a systematic review and meta-analysis to evaluate the immunogenicity and safety of co-administering seasonal influenza vaccines primarily with COVID-19 vaccines, as well as other routinely recommended vaccines according to national immunisation schedules. Given the potential clinical and logistical advantages of co-administration, we aimed to evaluate its immunological and safety implications compared with non-concomitant vaccination.

## Results

**Fig. 1:**
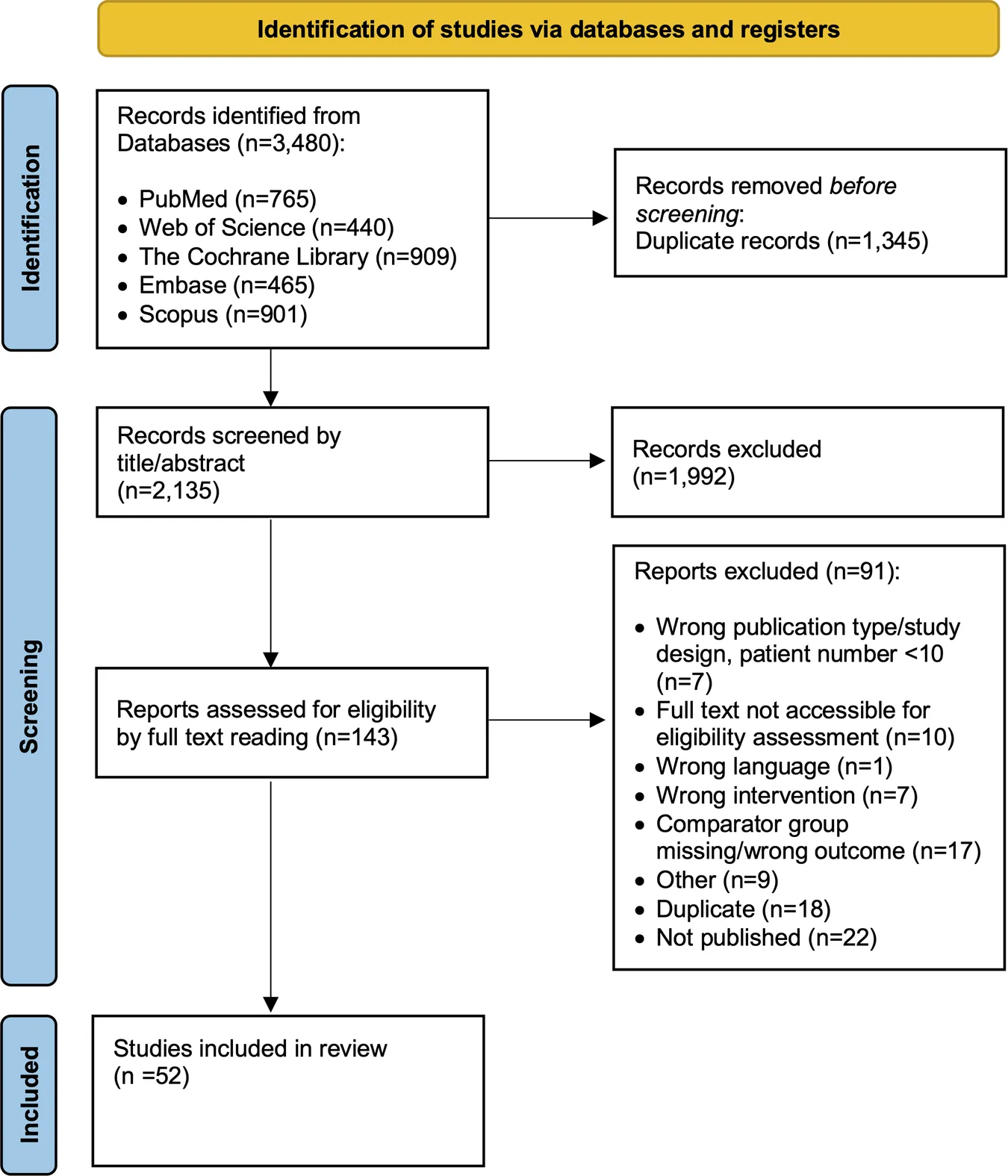
PRISMA Flow diagram of study search and selection.

Initially, a total of 3480 studies were identified by the search. After full-text examination of 143 articles, 52 met the inclusion criteria for this review. These studies, published between 1980 and 2024, encompassed 1,094,844 participants from 33 countries, representing the total populations reported across studies. The evidence base comprised 38 randomised controlled trials and 14 observational studies (Table 1). Detailed information on excluded articles and the PRISMA checklist can be found in the Supplementary Tables S2 and S4. The PRISMA flow diagram and reasons for exclusion are presented in Fig. 1.

**Table 1:** Characteristics of included studies

Study	Population (n)	Design	Country	Intervention
Aydillo et al. 2024	128	Cohort Study	Spain	Influenza + COVID-19 vaccine
Baj et al. 2022	64	Cohort Study	Italy	Influenza + COVID-19 vaccine
Choi et al. 2024	154	Non-randomised interventional trial	South Korea	Influenza + COVID-19 vaccine
Izikson et al. 2022	296	RCT	United States	Influenza + COVID-19 vaccine
Gonen et al. 2023	649	Cohort Study	Israel	Influenza + COVID-19 vaccine
Dulfer et al. 2023	154	RCT	The Netherlands	Influenza + COVID-19 vaccine
Radner et al. 2023	781	Cohort Study	Austria	Influenza + COVID-19 vaccine
Ramsay et al. 2023	248	RCT	Australia	Influenza + COVID-19 vaccine
Shenyu et al. 2022	480	RCT	China	Influenza + COVID-19 vaccine
Toback et al. 2021	15187	RCT	United Kingdom	Influenza + COVID-19 vaccine
Wagenhäuser et al. 2022	1231	Non-randomised interventional trial	Germany	Influenza + COVID-19 vaccine
Murdoch et al. 2023	1128	RCT	Australia; New Zealand	Influenza + COVID-19 vaccine
Moss et al. 2023	54	Cohort Study	Israel	Influenza + COVID-19 vaccine
Hause et al. 2022	981099	Cohort Study	United States	Influenza + COVID-19 vaccine
Lazarus et al. 2021	679	RCT	United Kingdom	Influenza + COVID-19 vaccine
Chen et al. 2022	1152	RCT	China	Influenza + COVID-19 vaccine
Moscara et al. 2023	1038	Cohort study	Italy	Influenza + COVID-19 vaccine
Cannon et al. 2023	1791	RCT	United States	Influenza + pneumococcal vaccine
Frenck et al. 2012	1116	RCT	United States	Influenza + pneumococcal vaccine
Nakashima et al. 2018	161	RCT	Japan	Influenza + pneumococcal vaccine
Ofori-Anyinam et al. 2017	356	RCT	Belgium	Influenza + pneumococcal vaccine
Severance et al. 2022	1200	RCT	United States	Influenza + pneumococcal vaccine
Thompson et al. 2019	882	RCT	United States	Influenza + pneumococcal vaccine
Schwarz et al. 2011	1190	RCT	Germany; The Netherlands; Belgium; Hungary	Influenza + pneumococcal vaccine
Jansen et al. 2008	579	RCT	The Netherlands	Influenza + pneumococcal vaccine
Song et al. 2015	224	RCT	South Korea	Influenza + pneumococcal vaccine
Song et al. 2016	1237	RCT	South Korea	Influenza + pneumococcal vaccine
Sočan et al. 2004	861	Cohort Study	Slovenia	Influenza + pneumococcal vaccine
Honkanen et al. 1996	9336	Non-randomised interventional trial	Finland	Influenza + pneumococcal vaccine
DeStefano et al. 1982	126	RCT	United States	Influenza + pneumococcal vaccine
Mufson et al. 1980	68	RCT	United States	Influenza + pneumococcal vaccine
Sadoff et al. 2021	180	RCT	United States	Influenza + RSV vaccine
Widagdo et al. 2023	771	RCT	The Netherlands	Influenza + RSV vaccine
Chandler et al. 2024	976	RCT	New Zealand; Panama; South Africa	Influenza + RSV vaccine
Athan et al. 2023	1403	RCT	Australia	Influenza + RSV vaccine
Zimmermann et al. 2013	954	RCT	France; Germany	Influenza + RSV vaccine
McNeil et al. 2007	720	RCT	Canada	Influenza + DTP vaccine
Sukumaran L et al. 2015	36844	Cohort Study	United States	Influenza + DTP vaccine
Glover et al. 2020	13758	Cohort Study	Australia	Influenza + DTP vaccine
Weston et al. 2009	1497	RCT	United States	Influenza + DTP vaccine
Breiman et al. 2009	2503	RCT	Bangladesh; Chile; Colombia; Malaysia; Peru; Philippines; Thailand	Influenza + Polio vaccine
Villanueva et al. 2022	2615	RCT	Australia	Influenza + BCG vaccine
Nolan et al. 2008	1251	RCT	United States	Influenza + MMR/Varicella vaccine
Lum et al. 2009	1233	RCT	Bangladesh; Belgium; Finland; Germany; Hong Kong; Lithuania; Malaysia; Mexico; Philippines; Poland; Singapore; South Korea; Thailand	Influenza + MMR vaccine
Nazareth et al. 2024	420	Cohort Study	United Kingdom; China; Australia; United States	Influenza + COVID-19 vaccine
Zhu et al. 2024	480	RCT	China	Influenza + pneumococcal vaccine
Clark et al. 2024	1045	RCT	Belgium; Finland; France; Spain; United Kingdom	Influenza + RSV vaccine
Buynak et al. 2024	1029	RCT	United States	Influenza + RSV vaccine
Naficy et al. 2024	995	RCT	United States	Influenza + COVID-19 vaccine
Huang et al. 2024	299	RCT	China	Influenza + COVID-19 vaccine
Chime et al. 2024	1586	RCT	Canada; Finland; South Korea; Spain; United States	Influenza + RSV vaccine
Bai et al. 2024	636	RCT	China	- Influenza + COVID-19 vaccine - Influenza + COVID-19 vaccine

### Influenza GMFR

**Fig. 2:**
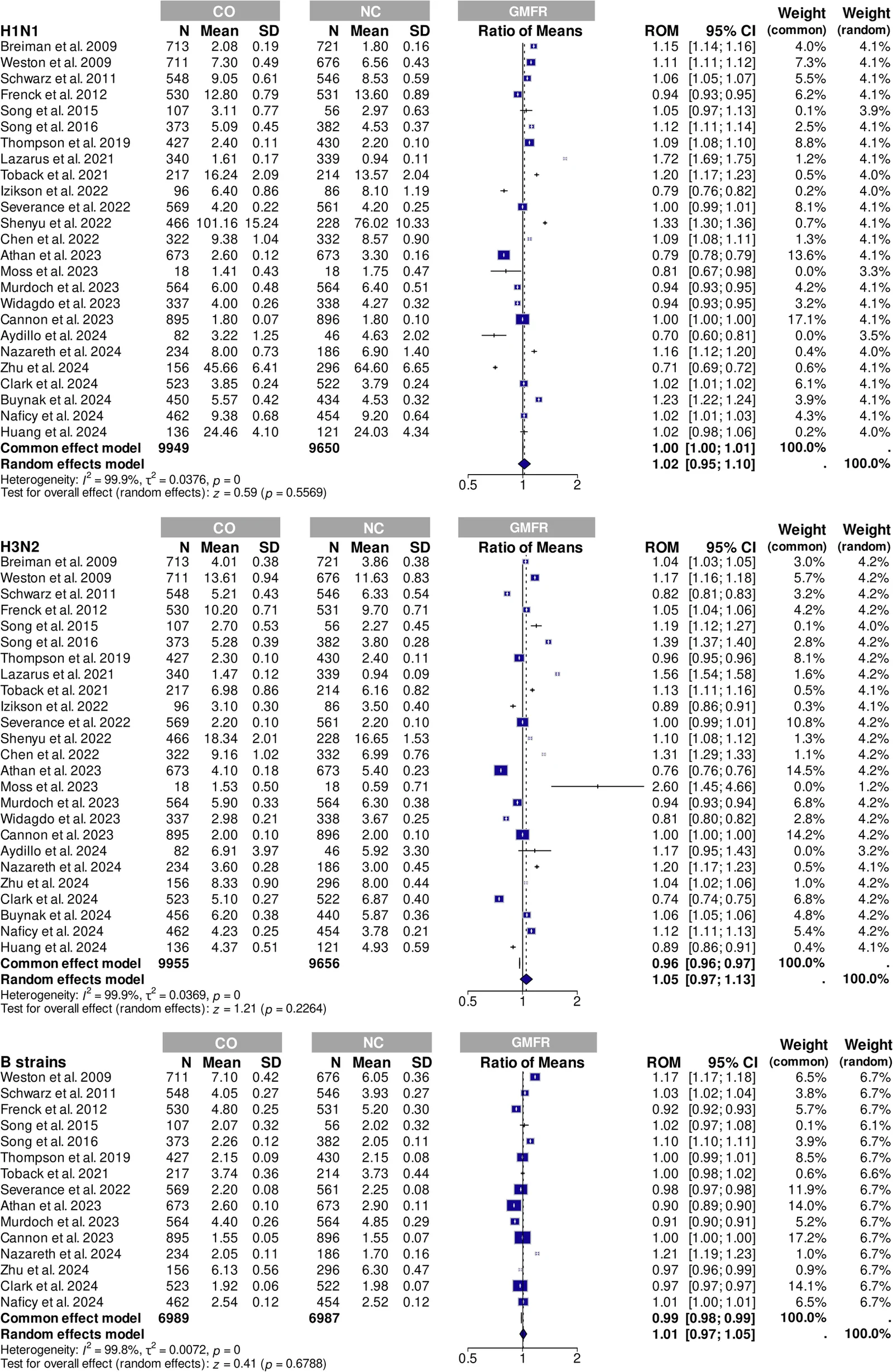
Influenza Ratio of Means (ROM) comparing geometric mean fold rise (GMFR) between co-administration (CO) and non-concomitant (NC) groups. Blue squares represent study-specific estimates, with square size proportional to study weight, and horizontal lines represent 95% confidence intervals. Diamonds represent overall estimates. ROM Ratio of Means, CO co-administration, NC non-concomitant administration.

For the primary outcome, 25 studies reporting on H1N1 and H3N2 and 15 studies reporting on the B strain were included in the GMFR analyses, comprising data from 26,893 individuals in the co-administration group and 26,293 in the non-concomitant administration group. Study publication dates ranged from 1980 to 2024. The GMFRs were similar between the co-administration and non-concomitant administration groups. There was no significant difference in the immune response across all three strains. The random-effects ratio of means (ROM) for H1N1 GMFR was 1.02 (95% CI: 0.95–1.10; *p* = 0.556) (Fig. 2 and Figure S1), though there was substantial heterogeneity across studies (*I*2 = 99.9%, *τ*2 = 0.0376). Similar patterns were observed for H3N2 (ROM 1.05 [95% CI: 0.97–1.13]; *p* = 0.226), and B strains (ROM 1.01 [95% CI: 0.97–1.05] *p* = 0.678) (Fig. 2; Supplementary Figures S2 and S3).

### Influenza seroprotection

**Fig. 3:**
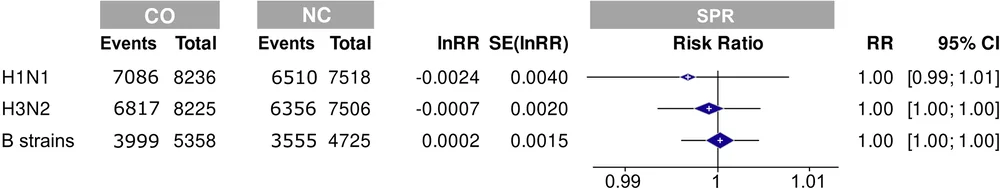
Pooled seroprotection risk ratios (RR) for influenza virus strains comparing co-administration (CO) and non-concomitant administration (NC) groups. Diamonds represent strain-specific estimates (weighted) with 95% CIs. RR values greater than 1 indicate higher seroprotection in the co-administration group. RR = risk ratio; CO = co-administration; NC = non-concomitant administration.

A total of 22 studies provided data on H1N1 and H3N2, and 16 studies reported on B lineage seroprotection rate, respectively. Across all influenza strains, the analysis included 21,819 patients in the co-administration group and 19,749 in the non-concomitant administration group. As shown in Fig. 3, the seroprotection rates were comparable between the two groups across all strains, with no evidence of reduced immunogenicity in any lineage: H1N1, RR: 1.00 (95% CI: 0.99–1.01; *p* = 0.559); H3N2, RR 1.00 (95% CI: 1.00–1.00; *p* = 0.733); and B strains, RR 1.00 (95% CI: 1.00–1.00; *p* = 0.910). Detailed stratified results for H1N1, H3N2, and B strains are presented in Supplementary Figures S4, S5 and S6.

### Safety

**Fig. 4:**
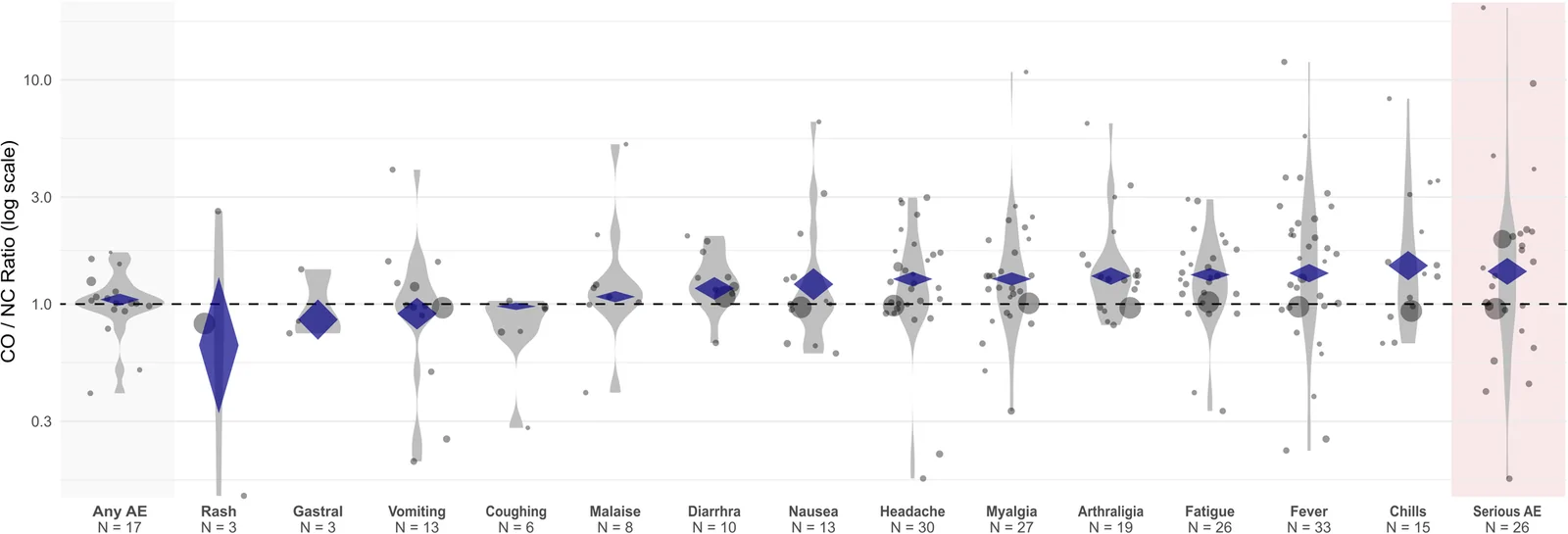
Ratios of adverse event (AE) proportions comparing co-administration (CO) with non-concomitant administration (NC) are shown on a log scale. Violin plots depict the distribution of study-level estimates, with individual study estimates shown as points and pooled estimates shown as blue diamonds. The dashed horizontal line indicates parity between groups (risk ratio [RR] = 1). Values above 1.0 indicate a higher AE proportion in the CO group relative to NC, whereas values below 1.0 indicate a higher AE proportion in the NC group. Numbers below each AE label indicate the number of included studies contributing estimates for that specific outcome. RR risk ratio, AE adverse event, CO co-administration, NC nonconcomitant administration.

Analysis of AE, including any reported AE, specific symptoms, and Grade ≥3 events, yielded heterogeneous results. While there was no overall difference in rates of AE between co- and non-concomitant administration (RR 1.05 [95% CI: 0.93–1.18]; *p* = 0.44) (Fig. 4 and Figure S7.1), serious AE were more likely to occur in the co-administration group (RR 1.41 [95% CI: 1.07–1.86]; *p* = 0.014) (Fig. 4 and Figure S7.2). This corresponded to an absolute pooled risk difference of 1.56 percentage points (95% CI: 0.38–2.74), or approximately 16 additional serious AE per 1,000 patients. For serious AE, 26 studies contributed to the pooled estimate and three zero-event studies were excluded. Across all AE outcomes, zero-event exclusions ranged from zero to three studies and are summarised in Table S11. Individuals in the co-administration group exhibited significantly higher rates of several specific adverse events, including arthralgia (RR 1.31 [95% CI: 1.10–1.57]; *p* = 0.002) (Figure S8), chills (RR 1.44 [95% CI: 1.07–1.94]; *p* = 0.017) (Figure S9), fatigue (RR 1.31 [95% CI: 1.13–1.52]; *p* < 0.001) (Figure S10), fever (RR 1.35 [95% CI: 1.11–1.63]; *p* = 0.002) (Figure S11), headache (RR 1.26 [95% CI: 1.07–1.47]; *p* = 0.004) (Figure S12) and myalgia (RR 1.27 [95% CI: 1.10–1.47]; *p* < 0.001) (Figure S13).

No statistically significant differences were observed for other AE including cough (Figure S14), diarrhea (Figure S15), gastrointestinal (Figure S16), malaise (Figure S17), nausea (Figure S18), rash (Figure S19), and vomiting (Figure S20). All safety data can be found in the Supplementary Figures S7–S20.

### Exploratory (COVID-19 GMFR)

**Fig. 5:**
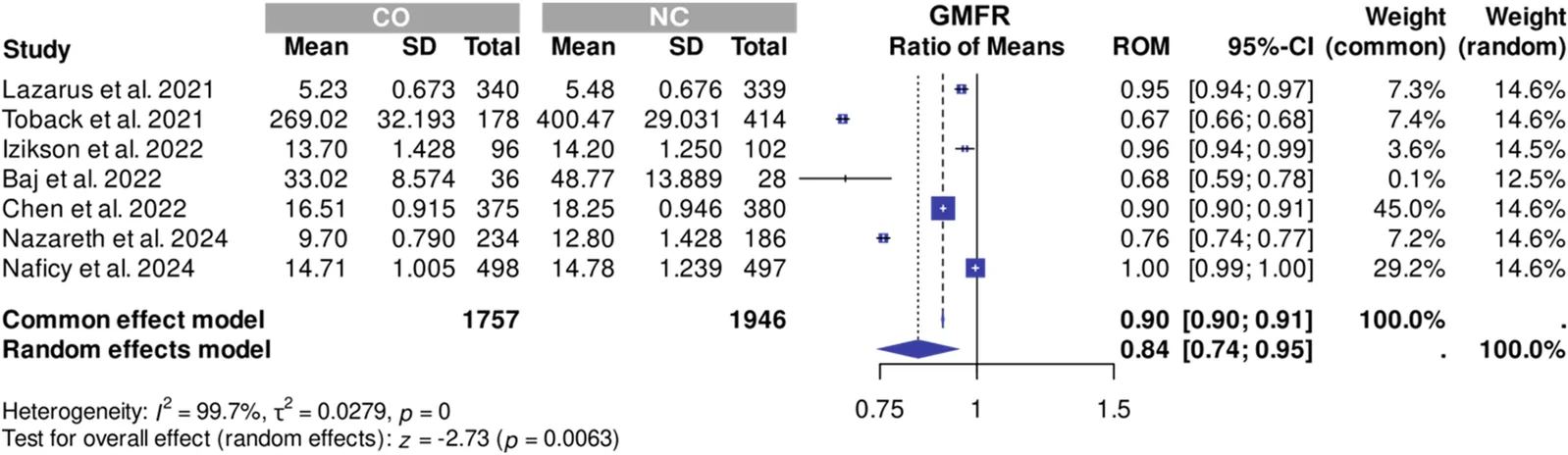
Pooled COVID-19 Ratio of Means (ROM) comparing geometric mean fold rise (GMFR) between co-administration (CO) and non-concomitant administration (NC) groups. Squares represent study-specific estimates, with square size proportional to study weight, and horizontal lines represent 95% confidence intervals. ROM values greater than 1 indicate higher GMFR in the co-administration group. ROM Ratio of Means, GMFR geometric mean fold rise, CO co-administration, NC non-concomitant administration.

For the exploratory COVID-19 GMFR analysis, seven studies met the eligibility criteria for this outcome by evaluating same-day co-administration of influenza and COVID-19 vaccines and reporting COVID-19 immunogenicity data with an eligible non-concomitant COVID-19 comparator. These studies comprised data from 1757 participants in the co-administration group and 1946 in the non-concomitant administration group. The overall GMFR was significantly lower in the co-administration group compared to the non-concomitant administration group (ROM 0.84 [95% CI: 0.74–0.95]; *p* = 0.006) in the REML (Fig. 5). Substantial heterogeneity was observed across studies (*I*2 = 99.7%, *τ*2 = 0.027), indicating significant between-study variability in the magnitude, but not the direction, of the effect. Therefore, the pooled estimate should be interpreted with caution as a summary of directionally consistent but highly heterogeneous study-level effects.

### Subgroup and sensitivity analyses

Age-stratified subgroup analyses showed a significant increase in GMFR for H3N2 with concurrent vaccination in younger participants (ROM 1.14 [95% CI: 1.03–1.27]; Figure S21). No statistically significant GMFR differences were observed for H1N1 or influenza B across age groups (Tables S7 and S9). Similar non-significant results were obtained in seroprotection rate analyses (Tables S7, S8, S9 and S10). In the younger COVID-19 subgroup, GMFR was significantly lower with co-administration than with non-concomitant administration (ROM 0.86 [95% CI: 0.75–0.99]; Figure S22).

Subgroup analysis by sex revealed no significant differences in GMFR or seroprotection rate between males and females across the majority of the strains examined. However, in the H3N2 strain, females exhibited a significantly higher immune response (ROM 1.18 [95% CI: 1.07–1.29]; Figure S23), with substantial heterogeneity (*I*2 = 99.9%, *τ*2 = 0.0187, *p* < 0.001).

No significant disparities in influenza GMFR and seroprotection rate were observed between non-RCT and RCT studies. In the COVID-19 analysis, non-RCT studies yielded a higher GMFR in the non-concomitant administration group compared to RCTs (ROM 0.73 [95% CI 0.66–0.81; Figure S24]). Further details can be found in Supplementary Tables S7, S8, S9 and S10.

Vaccine valency (trivalent vs. quadrivalent) did not influence GMFR or seroprotection rate. However, in a subgroup analysis limited to only two studies, co-administration with cell-based influenza vaccines was associated with higher GMFR compared to egg-based vaccines for H1N1 and H3N2 strains (ROM 1.22 [95% CI: 1.10–1.36] and ROM 1.19 [95% CI: 1.17–1.21]; Figures S25 and S26). Given the very small number of studies, this finding should be interpreted with caution. No major disparities of effect were observed with regard to the use of adjuvants except for a lower seroprotection rate in the H1N1 strain within the adjuvants group (RR 0.97 [95% CI: 0.95–0.99]; Figure S27). Exploratory subgroup analyses by geographic region should be interpreted cautiously, as some subgroups relied on only two comparative studies. No regional differences were observed for COVID-19 outcomes (Table S10). Influenza seroprotection rates were generally comparable across regions (Tables S7–S9). Only influenza B GMFR showed a lower relative response in southern-hemisphere studies after co-administration (ROM 0.90 [95% CI: 0.89–0.91]; *I*² = 87.2%) (Figure S28), but this finding remains highly exploratory.

### Quality assessment

Certainty of evidence assessment varied across outcomes (Table S5). For influenza immunogenicity, certainty was rated low for GMFR and moderate to high for seroprotection rates. For COVID-19, certainty was rated moderate for GMFR. Adverse event analyses showed low certainty for any AE, very low for serious AE, and very low to high depending on the specific reactogenic or predefined clinical AE assessed.

**Fig. 6:**
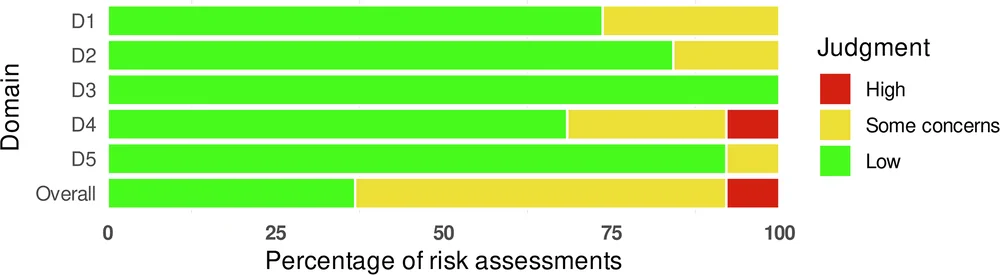
Proportional distribution of risk-of-bias assessments across included studies.

Assessment of study quality using RoB 2 adjudicated three studies as high risk of bias, 21 as intermediate risk, and 14 as low risk. The proportional results of the risk of bias assessment are presented in Fig. 6. The full assessment and traffic light plot is provided in Supplementary material (Table S6a). The observational studies were assessed separately using ROBINS-I. Among the 14 observational studies, seven were judged to be at high risk of bias and seven at intermediate risk, with none classified as low risk overall. The full assessment is provided in the Supplementary material (Table S6b). Visual assessment of the funnel plots did not indicate major asymmetry, suggesting little evidence of publication bias. However, most plots showed wide dispersion of estimates, indicating substantial heterogeneity not explained by the assessed subgroups.

## Discussion

This systematic review and meta-analysis found that co-administration of seasonal influenza vaccines with other routinely recommended vaccines (COVID-19, RSV, pneumococcal, DTP, polio, MMR and BCG) preserved influenza immunogenicity, with comparable GMFR and seroprotection rates to non-concomitant administration across all strains. However, co-administration was associated with higher rates of serious and certain predefined AEs. COVID-19 vaccine GMFR was significantly reduced under co-administration.

Our results align with most prior studies indicating that co-administration does not compromise immunogenicity^14,19–22^. However, unlike prior studies that primarily reported non-inferiority, our analysis identifies a more nuanced picture. For example, Lee et al. observed that same-arm co-administration produced a 1.2-fold higher influenza titre compared with different-arm administration, suggesting a potential synergistic effect^23^. Conversely, some studies have indicated attenuated immune responses, such as a 2023 report of reduced humoral responses to COVID-19 vaccines after co-administration^24^, Toback et al.’s findings of slightly lower COVID-19 antibody titres^14^, and Gonen et al.’s report of a 16% lower GMFR, though without statistical significance^25^. Through meta-analysis of these heterogeneous findings, our study quantitatively confirms that immunogenicity is broadly preserved while highlighting variation across vaccine types, study designs, and population subgroups.

Regarding safety, we identified a significant increase in the risk of serious and multiple reactogenicity-related events. This finding is consistent with several studies showing a higher rate of AE under co-administration. Hause et al., for instance, observed an 8–11% increase in reactogenicity^14,26^.

A previous meta-analysis suggested acceptable safety and immunogenicity of co-administration strategies^27^. However the analysis was only based on three published studies. A more recent qualitative systematic review confirmed these results but similarly did not report the elevated risks our analysis uncovered^28^. Our study strengthens the evidence by including comprehensive safety endpoints and highlighting specific AEs that warrant further investigation in future trials and pharmacovigilance efforts.

The higher frequency of serious and reactogenicity-related AEs occurred despite similar overall AE counts. This indicates that co-administration may alter the distribution of AE types rather than the total burden of reported events. Although mechanistic inference is beyond the scope of this meta-analysis, one possible hypothesis is that simultaneous activation of pattern-recognition receptors (e.g., Toll-like receptors) and inflammasome pathways amplifies systemic cytokine cascades (IL-1, IL-6, TNF-α), producing more pronounced inflammatory manifestations in a susceptible subset without increasing the overall number of affected individuals^29,30^.

Additionally, co-delivery may increase the antigenic and adjuvant load within draining lymph nodes, augmenting antigen presentation and cytokine spillover^31,32^. This effect may augment responses in individuals already near an activation threshold rather than expanding the total responder pool, consistent with threshold models of innate immune activation^33,34^.

These safety findings should still be interpreted with caution. Although the RR indicated an increased risk of serious AEs in the co-administration group, the corresponding absolute difference was modest, at approximately 16 additional serious AEs per 1000 patients. Therefore, the elevated relative risk should be interpreted in the context of a low absolute excess risk. Moreover, the association was not consistent in fixed-effects analyses, reflecting the substantial heterogeneity between studies. In addition, the exclusion of three zero-event studies and variability in AE reporting may have introduced bias in the pooled estimates.

Potential immunological mechanisms may help explain the observed patterns. Co-administration could influence innate immune activation and local inflammatory signalling, shaping subsequent antigen presentation and downstream adaptive immune responses. Consistent with this possibility, Lee et al. proposed that same-arm administration may elicit synergistic innate immune activation^23^. The observed attenuation in COVID-19 GMFR, most evident in younger adults and in non-RCTs, may be attributed to antigenic competition or cytokine-mediated interference, where the innate response to one vaccine modulates the adaptive response to another^14,35^. Although neutralising antibody titres are established correlates of protection for COVID-19 vaccines, the clinical relevance of the observed GMFR reduction remains uncertain. Previous studies have shown that humoral immune markers correlate with protection against SARS-CoV-2 infection, supporting the biological relevance of the observed reduction in COVID-19 GMFR^36–38^. However, a 16% reduction in GMFR cannot be directly translated into a proportional reduction in clinical protection, particularly across different vaccine platforms, populations and variants. This finding should also be interpreted cautiously because of the very high between-study heterogeneity observed for this outcome (*I*² = 99.7%).

Conversely, our subgroup findings suggest that younger patients exhibited enhanced responses to H3N2, implying that co-administration may offer immunological benefits in this population. A similar pattern was observed in young participants receiving COVID-19 vaccines. This likely reflects general age-related declines in immune responsiveness, a phenomenon well documented across vaccine platforms and consistent with previous studies reporting stronger vaccine-induced immunity in younger adults compared with those aged ≥65^33,39^.

Females exhibited a stronger H3N2 response, aligning with established sex-based immunological differences characterised by enhanced B lymphocyte activation and antibody production. Although speculative in the present context, this difference may be influenced by oestrogen signalling^40,41^.

Among influenza vaccine platforms, cell-based vaccines appeared to be associated with higher GMFR compared to egg-based vaccines in the co-administration group, likely due to avoidance of egg-adaptive mutations^42^. However, because this subgroup analysis was based on only two studies, this finding should be interpreted cautiously and should not be given the same weight as findings supported by larger evidence bases.

The geographical subgroup analysis was highly exploratory and limited by the small number of studies within each region. Accordingly, the lower relative influenza B GMFR observed in southern-hemisphere studies, which relied on only two studies, should be interpreted cautiously and not considered evidence of a true regional effect.

Our study has to be interpreted in the context of certain limitations. Substantial between-study heterogeneity was observed for several outcomes, limiting the precision and generalisability of the pooled estimates. This heterogeneity likely reflects clinical and methodological differences across studies, including vaccine platforms, co-administered vaccine types, study design, population characteristics, baseline immune status, prior infection or vaccination history, and assay methods. Although random-effects models account for between-study variability, pooling under conditions of extreme heterogeneity remains inherently limited. Accordingly, statistically significant pooled estimates should be interpreted cautiously as average effects across diverse contexts. The scarcity of long-term immunogenicity and effectiveness data prevents definitive conclusions about clinical protection. Furthermore, data from low-income countries were underrepresented, limiting applicability to settings where co-administration could yield the greatest public health impact.

Our analysis integrated data from both RCTs and non-RCTs, allowing broad assessment of immunogenicity and safety across diverse real-world settings. Importantly, findings were consistent in RCT-only subgroup analyses, supporting the robustness of the overall conclusions. Nevertheless, inclusion of observational evidence may have introduced unmeasured confounding, particularly from differences in baseline health, prior infection status, and AE reporting across observational studies.

Despite these limitations, this meta-analysis includes the largest number of studies on vaccine co-administration to date, integrating immunogenicity and safety data across multiple platforms and subgroups. The inclusion of 52 studies, stratified subgroup analyses, and comprehensive AE reporting strengthens the validity of our findings and avoids overgeneralization. The findings support current recommendations by the WHO and CDC that endorse co-administration as safe and effective. Despite these recommendations, real-world uptake remains limited^18,43^. Contributing factors include asynchronous vaccine availability, lack of integration into national immunisation schedules, and limited infrastructure to support simultaneous delivery^17,44^. Furthermore, persistent concerns among healthcare providers about potential reactogenicity and patient acceptability may also contribute to reluctance, despite reassuring trial data^13,45^.

Future research should prioritise RCTs assessing long-term immunogenicity, co-administration in high-risk or immunocompromised populations, and cost-effectiveness analyses to inform global policy. Overall, co-administration of seasonal influenza and COVID-19 vaccines demonstrated comparable immunogenicity for influenza and may therefore support integration into routine immunisation programmes. However, this potential benefit should be interpreted alongside some safety and immunogenicity considerations, including a potentially higher occurrence of serious and multiple predefined clinical AEs. COVID-19-specific GMFR was also significantly lower, indicating a reduced humoral response, although clinical relevance remains uncertain. Careful implementation and continued evaluation are therefore warranted to ensure the safe and effective integration of co-administration strategies into routine immunisation programmes.

## Methods

This systematic review and meta-analysis was registered at the International Prospective Register of Systematic Reviews (PROSPERO) in January 2024 (CRD42024502759). We followed the recommendations from the Cochrane Handbook for Systematic Reviews^46^. This review is reported in accordance with the Preferred Reporting Items for Systematic Reviews and Meta-Analyses (PRISMA) 2020 checklist^47^ presented in the Supplementary Table S1.

### Search strategy and selection criteria

We systematically searched PubMed, Embase, Web of Science, Scopus and the Cochrane Central Register of Controlled Trials (CENTRAL) from database inception until September 10, 2024 for eligible studies using controlled vocabulary and keywords related to: influenza vaccination, COVID-19 vaccination, co-administration, vaccine effectiveness and supplementary terms for other vaccines (inactivated or live-attenuated influenza, respiratory syncytial virus (RSV), pneumococcal, diphtheria-tetanus-pertussis (DTP), poliomyelitis, measles-mumps-rubella (MMR), and Bacillus Calmette–Guérin (BCG)) were included to capture relevant co-administration studies. The search strategy is detailed in Supplementary Table S2. We included human studies published in English or German that assessed same-day co-administration of influenza vaccines with other vaccines. No population-specific exclusions were applied a priori. Studies including immunocompromised individuals, pregnant women, paediatric populations, and adults were eligible for inclusion, provided they met the other predefined eligibility criteria. Detailed inclusion and exclusion criteria are provided in Supplementary Table S3.3. Eligible vaccine combinations are listed in Supplementary Table S12. The primary comparison of interest was same-day co-administration of seasonal influenza vaccine with COVID-19 or other routinely recommended vaccines, defined as administration within 24 h, versus a non-concomitant comparator. The comparator group was defined as non-concomitant vaccine administration, including sequential administration of the same vaccines on different days; administration of one component vaccine alone; or administration of one component vaccine with placebo or saline control. Comparator types were pooled to estimate the overall effect of same-day co-administration on immunogenicity and safety relative to the available non-concomitant comparator. Studies were excluded for *wrong intervention* if they did not assess concomitant vaccination within the 24-h window. Studies were excluded for *missing comparator* if they did not include an eligible non-concomitant comparator group. All exclusion reasons are reported in Supplementary Table S4.

Eligible studies reported immunogenicity and safety outcomes, included a comparator group (influenza vaccine alone, concomitant vaccine alone, or placebo), and enroled at least ten participants. All outcome domains used for study eligibility were defined a priori before screening and data extraction. Studies were eligible if they reported at least one predefined immunogenicity endpoint or at least one post-vaccination safety endpoint after vaccine co-administration. Eligible safety outcomes included solicited adverse events, unsolicited adverse events, severe adverse events, or serious adverse events.

For the immunogenicity synthesis, antibody titres were extracted from the prespecified nominal early post-vaccination assessment, defined as the scheduled assessment occurring 21–35 days after the relevant vaccine dose; where multiple eligible post-vaccination time points were reported, the value closest to day 28–30 was used. This window was prespecified to capture the early post-vaccination antibody response and is supported by evidence that haemagglutination-inhibition (HI) titres in older adults are comparable 2–4 weeks after influenza vaccination^48^. Immunogenicity data reported outside this prespecified post-vaccination assessment window were not included in the pooled immunogenicity synthesis. No restriction on publication year was applied. Both randomised controlled trials and observational study designs (cohort, case-control, or cross-sectional) were included to ensure a comprehensive synthesis of trial and real-world evidence. To account for design-related bias, we conducted predefined subgroup analyses restricted to RCTs for key outcomes. We excluded animal studies, non-comparative clinical reports, including case reports and case series, and non-primary research articles, such as editorials, reviews, and conference abstracts without sufficient data.

At least two authors (SA, EA or SH) independently screened all retrieved articles for inclusion criteria and removed duplicates. Discrepancies were resolved by discussion or adjudication by a third reviewer to reach consensus. Included studies were subsequently assessed for eligibility based on their full text using the same principle. We additionally performed a grey literature search by screening the reference lists of included studies and identifying further citations through targeted searching^49^.

### Data extraction and analysis

Data extraction was performed using a standardised extraction form. We extracted study characteristics (e.g. authors, year, country, design), participant demographics, details of the intervention (e.g. vaccine type, dose, adjuvants), and all reported immunogenicity, safety, and relevant clinical outcomes. In instances where data were not readily available in the trial reports, we requested additional data from corresponding authors.

The primary outcome was vaccine immunogenicity, quantified as the geometric mean fold rise (GMFR) in antigen-specific antibody titres from baseline (pre-vaccination) to follow-up (post-vaccination) measurements, comparing co-administration versus non-concomitant administration. For studies that did not directly report GMFR, it was calculated using geometric mean titre (GMT) values at baseline and follow-up measurements. Each of the three WHO-recommended seasonal influenza vaccine strains (H1N1, H3N2, and B) were analysed separately.

Secondary outcomes included the seroprotection rate, defined as the proportion of participants achieving HI antibody titres ≥1:40 post-vaccination regardless of baseline levels^19,50^. Additional secondary outcomes included the incidence of adverse events (AEs), serious AEs, and severe AEs, with severe AEs defined as grade 3 or higher according to Common Terminology Criteria for Adverse Events (CTCAE)^51^. Serious AEs were extracted according to the definitions used in the original studies, generally including medically attended events, life-threatening events, hospitalisation and persistent or clinically significant disability. We grouped severe and serious AEs as clinically significant adverse events, representing outcomes beyond mild or moderate reactogenicity. Because the studies did not consistently attribute AEs to specific vaccine components, safety outcomes were analysed at the regimen level to compare co-administration with non-concomitant administration. In addition, exploratory analyses were conducted for COVID-19 vaccine immunogenicity. Studies were eligible for analyses if they met the general review eligibility criteria, evaluated same-day co-administration of COVID-19 and other vaccines, included an eligible non-concomitant comparator for the COVID-19 vaccine response, and reported COVID-19 GMT or GMFR data suitable for estimating the relative antibody response.

Subgroup analyses were conducted to examine differences in the effect of the primary and secondary outcomes across a priori defined categories, including age, sex, study design (RCT vs. non-RCT) and vaccine characteristics (trivalent vs. quadrivalent, adjuvanted vs. non-adjuvanted formulation and cell-based vs. egg-based). Age subgroups were defined as *young* (upper age range <65 years or mean/median age +1 SD < 60 years), *aged* (lower age range >65 years or mean/median age -1 SD > 70 years) and *other* for intermediate or missing data. Sex based subgroups were defined as *male overrepresente*d (female participant ratio <45%), *female overrepresented* (ratio >55%) or *neutral* in between. For hemisphere-based analyses, studies were classified as *northern*, *southern*, or *mixed* according to the geographic location of the participating study centres relative to the equator.

The assessment of study quality was conducted by two investigators. RCTs were judged using the Cochrane Risk-of-Bias tool for Randomised Trials (RoB 2)^52^, which allowed for the classification of studies into categories of low, some, or high risk of bias. All non-randomised studies evaluating intervention effects, including non-randomised interventional studies and observational comparative studies, were assessed using the Risk Of Bias In Non-randomized Studies of Interventions (ROBINS-I)^53^. Because several studies reported multiple outcome types, ROBINS-I was applied at the study level using the most conservative applicable judgment across reported immunogenicity and safety outcomes. The quality and certainty of evidence for each outcome was assessed using the Grading of Recommendations, Assessment, Development and Evaluations (GRADE) approach^54^. Certainty was evaluated across the domains of *risk of bias*, *inconsistency*, *indirectness, imprecision*, and *other considerations*. Evidence was downgraded where applicable for risk of bias, substantial heterogeneity or inconsistency in effect estimates, indirectness, or imprecision based on prespecified minimal important differences, including a 10% absolute difference for seroprotection rate and RR = 1.25 for AEs. Because no established minimal important difference exists for GMFR, imprecision was not formally assessed for this outcome. Certainty was instead considered in relation to indirectness, as immunogenicity outcomes are surrogate outcomes. Certainty was rated conservatively where evidence included observational studies alongside randomised trials, and no upgrading criteria were applied. Detailed information regarding study characteristics (Tables S3.1, S3.2, S4) and the quality assessment process, including outcome-specific GRADE judgments, is provided in the Supplement (Table S5 and S6),

Meta-analyses were conducted when at least three studies reported the same prespecified outcome and were sufficiently similar clinically and methodologically to support pooling. Similarity was assessed based on vaccine/intervention characteristics, outcome definition, and timing of assessment. Outcomes that did not meet these criteria were not included in quantitative synthesis. Statistical analysis was carried out using the *meta* (v8.1.0) package^55^ in the *R version 4.5.1* (2025-06-13) programming language (R Core Team 2021).

Primary endpoints and analyses were pre-specified, while exploratory analyses were conducted *post hoc* after the main results became available. For GMFR and seroprotection rate, pooled analyses used the prespecified nominal early post-vaccination assessment, defined as 21–35 days after the relevant vaccine dose. When studies reported multiple post-vaccination assessments within or near this window, the value closest to day 28–30 was extracted, and exploratory earlier or longer-term time points were excluded from the pooled analyses. For meta-analysis of continuous outcomes, pooled estimates of ratio of means (ROM) are calculated using a random-effects model (REML) with inverse-variance weighting to account for between-study heterogeneity. Binary outcomes are analysed analogously by estimating the pooled relative risk (RR). Study-level estimates are reported with 95% confidence intervals (CIs). Subgroup differences were evaluated using a *χ*^2^ test for heterogeneity between subgroups. Studies with zero events in one arm were excluded from RR calculations when appropriate. Between-study heterogeneity was assessed by estimating *τ*^2^ using restricted maximum likelihood and quantified with the *I*^2^ statistic, with higher values indicating greater heterogeneity. Publication bias was assessed using visual inspection of funnel plots and Egger’s regression test when ≥3 studies were available. Asymmetry was formally assessed using both a linear regression test^56^ and a rank correlation test^57^.

When 95% CIs for GMTs were available, standard deviations (SD) were derived on the log scale and propagated to estimate uncertainty in GMFR. Definitions of outcomes and participant subgroups are provided in supplementary Table S3.2.

## Supplementary information

Supplementary_material.

## Data Availability

All meta-level data and extracted study datasets used in this meta-analysis are available from the corresponding author upon reasonable request. Requests will be reviewed by the study investigators, and a data use agreement may be required. No individual participant-level data were collected for this study.

## Code availability

The analyses were conducted in R version 4.5.1 using the *meta* package version 8.1.0. R code for conducting meta-analyses and the corresponding extracted data is available from the corresponding author upon reasonable request. No proprietary code was used.
