# Supplementary material for: Immunogenicity and safety of seasonal influenza vaccine co-administered with other vaccines: a systematic review and meta-analysis
Source: NPJ Vaccines. 2026 Sep 2;11:204. doi: 10.1038/s41541-026-01548-z (PMC13728179; doi:10.1038/s41541-026-01548-z)
Supplement: Supplementary file 1 [file 41541_2026_1548_MOESM1_ESM.pdf]

Supplementary Material

This appendix has been provided by the authors to give readers additional information about their work.

Supplement to: Samuel Asri, Paraskevi C. Fragkou, Elena Ahrens, Svenja S. Hartmann, Clemens Thölken, Chrysanthi Skevaki
Immunogenicity and safety of seasonal influenza vaccine co-administered with other vaccines: a systematic review and meta-analysis

Table of contents

Table S1: PRISMA checklist 1
Table S2: Search strategy 5
Search terms combination 7
Search results 7
Table S3.1 : Study characteristics 8
Table S3.2 : Definitions 11
Table S3.3 Inclusion and exclusion criteria for study selection and quantitative synthesis 12
Table S4: Exclusion Table 13
List of studies excluded at full-text screening stage 14
Table S5: Certainty of evidence 18
S5.1 Immunogenicity outcomes in influenza vaccine group 18
S5.2 Immunogenicity outcomes in COVID-19 vaccine group. 20
S5.3 Adverse events incidence 21
Table S6a: Risk of bias assessment – ROB2 23
Table S6b: Risk of bias assessment – ROBINS-I 25
Supplementary figures 26
Primary outcome 26
Figure S1: GMFR Influenza H1N1 26
Figure S2: GMFR Influenza H3N2 27
Figure S3: GMFR Influenza B 28
Secondary outcome: seroprotection 29
Figure S4: Seroprotection rate Influenza H1N1 29
Figure S5: Seroprotection rate Influenza H3N2 30
Figure S6: Seroprotection rate Influenza B 31
Secondary outcome: safety 32
Figure S7.1: adverse events - any 32
Figure S7.2: adverse events - (serious and/or grade ≥3) 33
Figure S8: adverse events - arthralgia 34
Figure S9: adverse events - chills 35
Figure S10: adverse events – fatigue 36
Figure S11: adverse events – fever 37

Figure S12: adverse events – headache....38
Figure S13: adverse events - myalgia....39
Figure S14: adverse events - cough....40
Figure S15: adverse events - diarrhea....41
Figure S16: adverse events - gastrointestinal....42
Figure S17: adverse events - malaise....43
Figure S18: adverse events - nausea....44
Figure S19: adverse events - rash....45
Figure S20: adverse events - vomiting....46
Subgroup analyses....47
Age subgroups....47
Figure S21: GMFR Influenza H3N2 - age subgroup analysis....47
Figure S22: GMFR COVID-19 - age subgroup analysis....48
Sex subgroups....49
Figure S23: GMFR Influenza H3N2 - sex subgroup analysis....49
Study and vaccine type....50
Figure S24: GMFR COVID-19 - RCT/non-RCT subgroup analysis....50
Figure S25: GMFR Influenza H1N1 - cell-based/egg-based....51
Figure S26: GMFR Influenza H3N2 - cell-based/egg-based....52
Figure S27: SPR Influenza H1N1 - Adjuvants vs no adjuvants....53
Figure S28: GMFR Influenza B - north vs. south....54
Table S7: subgroup analyses for Influenza H1N1....55
Table S8: subgroup analyses for Influenza H3N2....56
Table S9: subgroup analyses for Influenza B....58
Table S10: subgroup analyses for COVID-19....59
Table S11: Number of studies contributing to AE pooled estimate and zero-event studies excluded....59
Table S12: Eligible seasonal influenza vaccine co-administration combinations and comparator groups[1]....60

Table S1: PRISMA checklist

Section and Topic	Item #	Checklist item	Location where item is reported
**TITLE**			
Title	1	Identify the report as a systematic review.	Page 1
**ABSTRACT**			
Abstract	2	See the PRISMA 2020 for Abstracts checklist.	Page 3
**INTRODUCTION**			
Rationale	3	Describe the rationale for the review in the context of existing knowledge.	Page 5-6
Objectives	4	Provide an explicit statement of the objective(s) or question(s) the review addresses.	Page 6
**METHODS**			
Eligibility criteria	5	Specify the inclusion and exclusion criteria for the review and how studies were grouped for the syntheses.	Pages 23–25; Supplementary Table S3.3
Information sources	6	Specify all databases, registers, websites, organisations, reference lists and other sources searched or consulted to identify studies. Specify the date when each source was last searched or consulted.	Page 23; Supplementary Table S2
Search strategy	7	Present the full search strategies for all databases, registers and websites, including any filters and limits used.	Page 23; Supplementary Table S2
Selection process	8	Specify the methods used to decide whether a study met the inclusion criteria of the review, including how many reviewers screened each record and each report retrieved, whether they worked independently, and if applicable, details of automation tools used in the process.	Pages 24–25
Data collection process	9	Specify the methods used to collect data from reports, including how many reviewers collected data from each report, whether they worked independently, any processes for obtaining or confirming data from study investigators, and if applicable, details of automation tools used in the process.	Page 25
Data items	10a	List and define all outcomes for which data were sought. Specify whether all results that were compatible with each outcome domain in each study were sought (e.g. for all measures, time points, analyses), and if not, the methods used to decide which results to collect.	Pages 25–26; Supplementary Table S3.2
	10b	List and define all other variables for which data were sought (e.g. participant and intervention characteristics, funding sources). Describe any assumptions made about any missing or unclear information.	Pages 25–26; Supplementary Tables S3.1 and S3.2

Study risk of bias assessment	11	Specify the methods used to assess risk of bias in the included studies, including details of the tool(s) used, how many reviewers assessed each study and whether they worked independently, and if applicable, details of automation tools used in the process.	Page 27; Supplementary Tables S6a and S6b
Effect measures	12	Specify for each outcome the effect measure(s) (e.g. risk ratio, mean difference) used in the synthesis or presentation of results.	Pages 25–26 and 28
Synthesis methods	13a	Describe the processes used to decide which studies were eligible for each synthesis (e.g. tabulating the study intervention characteristics and comparing against the planned groups for each synthesis (item #5)).	Pages 23–28; Supplementary Tables S3.2 and S3.3
	13b	Describe any methods required to prepare the data for presentation or synthesis, such as handling of missing summary statistics, or data conversions.	Pages 25 and 28
	13c	Describe any methods used to tabulate or visually display results of individual studies and syntheses.	Pages 25–28; Figures 3–6 and Supplementary Figures S1–S28
	13d	Describe any methods used to synthesize results and provide a rationale for the choice(s). If meta-analysis was performed, describe the model(s), method(s) to identify the presence and extent of statistical heterogeneity, and software package(s) used.	Pages 27–28
	13e	Describe any methods used to explore possible causes of heterogeneity among study results (e.g. subgroup analysis, meta-regression).	Pages 26 and 28
	13f	Describe any sensitivity analyses conducted to assess robustness of the synthesized results.	Pages 13–15 and 18
Reporting bias assessment	14	Describe any methods used to assess risk of bias due to missing results in a synthesis (arising from reporting biases).	Page 28
Certainty assessment	15	Describe any methods used to assess certainty (or confidence) in the body of evidence for an outcome.	Page 27; Supplementary Table S5
**RESULTS**			
Study selection	16a	Describe the results of the search and selection process, from the number of records identified in the search to the number of studies included in the review, ideally using a flow diagram.	Page 7; Figure 2
	16b	Cite studies that might appear to meet the inclusion criteria, but which were excluded, and explain why they were excluded.	Supplementary Table S4

Study characteristics	17	Cite each included study and present its characteristics.	Page 7; Table 1; Supplementary Table S3.1
Risk of bias in studies	18	Present assessments of risk of bias for each included study.	Pages 15–16; Supplementary Tables S6a and S6b
Results of individual studies	19	For all outcomes, present, for each study: (a) summary statistics for each group (where appropriate) and (b) an effect estimate and its precision (e.g. confidence/credible interval), ideally using structured tables or plots.	Figures 3–6; Supplementary Figures S1–S28
Results of syntheses	20a	For each synthesis, briefly summarise the characteristics and risk of bias among contributing studies.	Pages 7 and 15–16; Table 1; Supplementary Tables S3.1, S6a and S6b
	20b	Present results of all statistical syntheses conducted. If meta-analysis was done, present for each the summary estimate and its precision (e.g. confidence/credible interval) and measures of statistical heterogeneity. If comparing groups, describe the direction of the effect.	Pages 8–15; Figures 3–6; Supplementary Figures S1–S28; Supplementary Tables S7–S11
	20c	Present results of all investigations of possible causes of heterogeneity among study results.	Pages 13–15; Supplementary Figures S21–S28; Supplementary Tables S7–S10
	20d	Present results of all sensitivity analyses conducted to assess the robustness of the synthesized results.	Pages 13–15 and 18
Reporting biases	21	Present assessments of risk of bias due to missing results (arising from reporting biases) for each synthesis assessed.	Page 15
Certainty of evidence	22	Present assessments of certainty (or confidence) in the body of evidence for each outcome assessed.	Page 15; Supplementary Table S5
**DISCUSSION**			
Discussion	23a	Provide a general interpretation of the results in the context of other evidence.	Pages 17–20
	23b	Discuss any limitations of the evidence included in the review.	Pages 18 and 20–21

	23c	Discuss any limitations of the review processes used.	Pages 20–21
	23d	Discuss implications of the results for practice, policy, and future research.	Pages 21–22
**OTHER INFORMATION**			
Registration and protocol	24a	Provide registration information for the review, including register name and registration number, or state that the review was not registered.	Page 23
	24b	Indicate where the review protocol can be accessed, or state that a protocol was not prepared.	Page 23
	24c	Describe and explain any amendments to information provided at registration or in the protocol.	Page 23
Support	25	Describe sources of financial or non-financial support for the review, and the role of the funders or sponsors in the review.	Pages 29–30
Competing interests	26	Declare any competing interests of review authors.	Page 30
Availability of data, code and other materials	27	Report which of the following are publicly available and where they can be found: template data collection forms; data extracted from included studies; data used for all analyses; analytic code; any other materials used in the review.	Page 29

Table S2: Search strategy

**1**	**Influenza**<br>((influenza[Title/Abstract]) OR (influenza[MeSH]) OR (flu[MeSH]) OR (flu[Title/Abstract]) OR (seasonal influenza[Title/Abstract]) OR (pandemic influenza[Title/Abstract]) OR (H5N1[Title/Abstract]) OR (H1N1[Title/Abstract]) OR (influenza virus[Title/Abstract]))
**2**	**Vaccination**<br>((vaccination[Title/Abstract]) OR (vaccine[MeSH]) OR (vaccination[MeSH]) OR (immunisation[Title/Abstract]) OR (immunization[MeSH]) OR (immunisation[Title/Abstract]) OR (inoculation[Title/Abstract]) OR (immunity[Title/Abstract]) OR (quadrivalent influenza vaccine*[Title/Abstract]) OR (quadrivalent flu vaccine[Title/Abstract]) OR (tetravalent influenza vaccine*[Title/Abstract]) OR (live attenuated influenza vaccine*[Title/Abstract]) OR (live attenuated flu vaccine*[Title/Abstract]) OR (Afluria Quadrivalent[Title/Abstract]) OR (Fluarix[Title/Abstract]) OR (Fluzone[Title/Abstract]) OR (FluMist Quadrivalent[Title/Abstract]) OR (Afluria Tetra[Title/Abstract]) OR (Efluel-da[Title/Abstract]) OR (Flu-ad Tetra[Title/Abstract]) OR (Flucel-vax Tetra[Title/Abstract]) OR (Fluenz Tetra[Title/Abstract]))
**2.1**	**Influenza and vaccination combined**<br>((influenza vaccine*[Title/Abstract]) OR (influenza vaccine[MeSH]) OR (flu vaccine[MeSH]) OR (flu vaccine*[Title/Abstract]) OR (seasonal influenza vaccine*[Title/Abstract]) OR (pandemic influenza vaccine*[Title/Abstract]) OR (H5N1[Title/Abstract]) OR (H1N1[Title/Abstract]) OR (influenza virus vaccine*[Title/Abstract]) OR (influenza immun*[Title/Abstract]) OR (influenza immunisation[MeSH]) OR (influenza inoculation[Title/Abstract]) OR (flu immun*[Title/Abstract]) OR (flu immunisation[MeSH]) OR (flu inoculation[Title/Abstract]) OR (Inactivated Influenza Vaccine*[Title/Abstract]) OR (quadrivalent influenza vaccine*[Title/Abstract]) OR (quadrivalent vaccine*[Title/Abstract]) OR (quadrivalent flu vaccine[Title/Abstract]) OR (tetravalent influenza vaccine*[Title/Abstract]) OR (tetravalent vaccine*[Title/Abstract]) OR (Quadrivalent Seasonal Influenza Vaccine*[Title/Abstract]) OR (live attenuated influenza vaccine*[Title/Abstract]) OR (live attenuated flu vaccine*[Title/Abstract]) OR (Afluria Quadrivalent[Title/Abstract]) OR (Fluarix[Title/Abstract]) OR (Fluzone[Title/Abstract]) OR (FluMist Quadrivalent[Title/Abstract]) OR (Afluria Tetra[Title/Abstract]) OR (Efluel-da[Title/Abstract]) OR (Flu-ad Tetra[Title/Abstract]) OR (Flucel-vax Tetra[Title/Abstract]) OR (Fluenz Tetra[Title/Abstract]))
**3**	**Covid**<br>((SARS-CoV-2[MeSH]) OR (COVID-19[MeSH]) OR (Covid-19[Title/Abstract]) OR (Coronavirus[MeSH]) OR (Coronavirus[Title/Abstract]) OR (coronavirus disease 2019[Title/Abstract]) OR (COVID[Title/Abstract]))
**4**	**Covid Vaccines**<br>((COVID vaccine[MeSH]) OR (COVID-19 vaccine[MeSH]) OR (SARS-CoV-2 vaccine[MeSH]) OR (Omicron BA.4/BA.5[Title/Abstract]) OR (BBV152[Title/Abstract]) OR (BBIBP-CorV[Title/Abstract]) OR (AZD1222[Title/Abstract]) OR (BNT162b2[Title/Abstract]) OR (NVX-CoV2373[Title/Abstract]) OR (COVID-19 vaccine*[Title/Abstract]) OR (COVID vaccine*[Title/Abstract]) OR (SARS-CoV-2 vaccine*[Title/Abstract]) OR (Coronavirus vaccine*[Title/Abstract]) OR (covid-19 immun*[Title/Abstract]) OR (SARS-CoV-2 immun*[Title/Abstract]) OR (Pfizer-BioNTech vaccine*[Title/Abstract]) OR (Moderna vaccine*[Title/Abstract]) OR (Johnson & Johnson vaccine*[Title/Abstract]) OR (AstraZeneca vaccine*[Title/Abstract]) OR (Covaxin[Title/Abstract]) OR (Novavax vaccine*[Title/Abstract]) OR (Spikevax[Title/Abstract]) OR (Comirnaty[Title/Abstract]) OR (BBV152[Title/Abstract]) OR (BBIBP-CorV[Title/Abstract]) OR (AZD1222[Title/Abstract]) OR (Bimer-vax[Title/Abstract]) OR (Omi-cron XBB.1.5[Title/Abstract]) OR (Omi-cron BA.4-5[Title/Abstract]) OR (Omi-cron BA.1[Title/Abstract]) OR (Jcov-den[Title/Abstract]) OR (Nu-vax-ovid[Title/Abstract]) OR (Nu-vax-ovid XBB.1.5[Title/Abstract]) OR (NVX-CoV2373[Title/Abstract]) OR (Spike-vax XBB.1.5[Title/Abstract]) OR (Vaxzevria[Title/Abstract]) OR (covid-19 mRNA vaccine*[Title/Abstract]))
**5**	**Co-Administration**<br>((coadministration[Title/Abstract]) OR (coadministered[Title/Abstract]) OR (co-administration[Title/Abstract]) OR (co-administered[Title/Abstract]) OR (co-injection[Title/Abstract]) OR (coinjection[Title/Abstract]) OR (concomitant administration[Title/Abstract]) OR (concomitant*[Title/Abstract]) OR (concomitant injection[Title/Abstract]) OR (concomitant vaccination[Title/Abstract]) OR (covaccination[Title/Abstract]) OR (co-vaccination[Title/Abstract]) OR (in combination with[Title/Abstract]) OR (administered concomitantly[Title/Abstract]) OR (combined with[Title/Abstract]) OR (in conjunction with[Title/Abstract]) OR (simultaneous administration[Title/Abstract]) OR (together with[Title/Abstract]) OR (simultaneous injection[Title/Abstract]) OR (combined administration[Title/Abstract]) OR (combined injection[Title/Abstract]) OR (concurrent administration[Title/Abstract]) OR (concurrent injection[Title/Abstract]) OR (concomitant use[Title/Abstract]) OR (used jointly[Title/Abstract]) OR (administered simultaneously[Title/Abstract]))
**6**	**Effectiveness**

	((effectiveness[Title/Abstract]) OR (useful*[Title/Abstract]) OR (potency[Title/Abstract]) OR (advantage[Title/Abstract]) OR (efficacy[Title/Abstract]))
7	**Other Vaccines:**<br>**a. RSV (+vaccination)**<br>(Respiratory Syncytial Virus[MeSH]) OR (RSV[Title/Abstract]) OR (Respiratory Syncytial Virus[Title/Abstract]) OR (Human Respiratory Syncytial Virus[Title/Abstract]) OR (HRSV[Title/Abstract]) OR (Pneumovirus[Title/Abstract]) OR (Paramyxovirus[Title/Abstract]) OR (Syncytial Virus, Human[Title/Abstract]) OR (RSV vaccine*[Title/Abstract]) OR (RSV immun*[Title/Abstract]) OR (Respiratory Syncytial Virus immun*[Title/Abstract]) OR (Respiratory Syncytial Virus vaccine*[Title/Abstract]) OR (Arexvy[Title/Abstract]) OR (Abrysvo[Title/Abstract]) OR (RSVPreF3[Title/Abstract]) OR (RSVpreF[Title/Abstract])<br><br>**b. MMR (mumps, measles, rubella)**<br>(Mumps[Title/Abstract]) OR (Mumps[MeSH]) OR (mumps vaccine[MeSH]) OR (Epidemic Parotitis[Title/Abstract]) OR (Infectious Parotitis[Title/Abstract]) OR (Mumps Virus[Title/Abstract]) OR (MMR[Title/Abstract]) OR (Mumps Measles Rubella[Title/Abstract]) OR (Mumps-Measles-Rubella[Title/Abstract]) OR (Mumps Measles Rubella[MeSH]) OR (Rubella[Title/Abstract] OR (German Measles[Title/Abstract]) OR (Rubella Virus[Title/Abstract]) OR (MMR Vaccin*[Title/Abstract]) OR (Mumps Measles Rubella Vaccine*[Title/Abstract]) OR (Mumps Measles Rubella immun*[Title/Abstract]) OR (MMR immun*[Title/Abstract]) OR (Priorix[Title/Abstract]) OR (Pri-or-ix-Tetra[Title/Abstract]) OR (ProQuad[Title/Abstract]) OR (M-M-R II[Title/Abstract])<br><br>**c. Pneumococcus**<br>(Pneumococcal[Title/Abstract]) OR (Streptococcus pneumoniae[Title/Abstract]) OR (Pneumococcus[Title/Abstract]) OR (Pneumonia Vaccine[Title/Abstract]) OR (Pneumonia Vaccin[MeSH]) OR (Pneumococcal vaccine*[Title/Abstract]) OR (Pneumococcal immun*[Title/Abstract]) OR (Pneumococcal Conjugate Vaccine*[Title/Abstract]) OR (PCV[Title/Abstract]) OR (PCV15[Title/Abstract]) OR (PCV20[Title/Abstract]) OR (Apexxnar[Title/Abstract]) OR (Pneu-mo-coc-cal Polysac-cha-ride Vac-cine*[Title/Abstract]) OR (Pneumovax[Title/Abstract]) OR (Syn-florix[Title/Abstract]) OR (Pre-ve-nar 13[Title/Abstract])<br><br>**d. Hepatitis B**<br>(Hepatitis B[Title/Abstract]) OR (Hepatitis B[MeSH]) OR (Hepatitis B vaccine[MeSH]) OR (HBV[Title/Abstract]) OR (Serum hepatitis[Title/Abstract]) OR (Hep B[Title/Abstract]) OR (Anti-HBs[Title/Abstract]) OR (Hepatitis B Vaccine*[Title/Abstract]) OR (Hepatitis B Immun*[Title/Abstract]) OR (Am-bir-ix[Title/Abstract]) OR (En-ger-ix B[Title/Abstract]) OR (Twinrix[Title/Abstract]) OR (Recombivax HB[Title/Abstract])<br>**e. Rotavirus**<br>(Rotavirus[Title/Abstract]) OR (Rotavirus[MeSH]) OR (Rotavirus vaccine[MeSH]) OR (RV[Title/Abstract]) OR (Rotavirus vaccine*[Title/Abstract]) OR (RV vaccine*[Title/Abstract]) OR (Rotavirus Immun*[Title/Abstract]) OR (RV immun*[Title/Abstract]) OR (Rotarix[Title/Abstract]) OR (RotaTeq[Title/Abstract])<br>**f. DTP: Diphtheria, tetanus, Pertussis**<br>(Diphtheria[Title/Abstract]) OR (Diphtheria[MeSH]) OR (DTP[Title/Abstract]) OR (Tetanus[Title/Abstract]) OR (Tetanus[MeSH]) OR (Pertussis[Title/Abstract]) OR (Pertussis[MeSH]) OR (Diphtheria vaccine*[Title/Abstract]) OR (Tetanus vaccine*[Title/Abstract]) OR (Pertussis vaccine*[Title/Abstract]) OR (Diphtheria immun*[Title/Abstract]) OR (Tetanus immun*[Title/Abstract]) OR (Pertussis immun*[Title/Abstract]) OR (DTP vaccine*[Title/Abstract]) OR (Adacel[Title/Abstract]) OR (Boostrix[Title/Abstract]) OR (Daptacel[Title/Abstract])<br>**g. Tuberculosis (BCG)**<br>(Tuberculosis[Title/Abstract]) OR (Tuberculosis[MeSH]) OR (TBC[Title/Abstract]) OR (Tuberculosis vaccine*[Title/Abstract]) OR (Tuberculosis immun*[Title/Abstract]) OR (TBC vaccine*[Title/Abstract]) OR (TBC immun*[Title/Abstract]) OR (BCG[Title/Abstract]) OR (BCG Vaccination[Title/Abstract]) OR (Bacille Calmette-Guerin[Title/Abstract]) OR (Bacille Calmette Guerin[Title/Abstract])<br>**h. H. influenzae**<br>(H. influenzae[Title/Abstract]) OR (H. influenzae[MeSH]) OR (Haemophilus influenzae[Title/Abstract]) OR (Haemophilus influenzae[MeSH]) OR (H. influenzae vaccine*[Title/Abstract]) OR (Haemophilus influenzae vaccine*[Title/Abstract]) OR (H. influenzae immun*[Title/Abstract]) OR (Haemophilus influenzae immun*[Title/Abstract]) OR (Pentacel[Title/Abstract]) OR (Pen-tavac[Title/Abstract]) OR (Vaxelis[Title/Abstract]) OR (Act-HIB[Title/Abstract]) OR (Hexy-on[Title/Abstract]) OR (Hexaci-ma[Title/Abstract]) OR (Hi-ber-ix[Title/Abstract]) OR (In-fan-rix[Title/Abstract])<br>**i. Poliomyelitis**<br>**j.** (Poliomyelitis[Title/Abstract]) OR (Poliomyelitis[MeSH]) OR (Polio[Title/Abstract]) OR (Polio[MeSH]) OR (Poliomyelitis vaccine*[Title/Abstract]) OR (Polio vaccine*[Title/Abstract]) OR (Poliomyelitis immun*[Title/Abstract]) OR (Polio immun*[Title/Abstract]) OR (Boost-rix Po-lio[Title/Abstract]) OR (Imovax Po-lio[Title/Abstract])<br>**k. HPV**

	(HPV[Title/Abstract]) OR (Human Papillomavirus[Title/Abstract]) OR (Human Papillomavirus[MeSH]) OR (HPV vaccine*[Title/Abstract]) OR (HPV immun*[Title/Abstract]) OR (Human Papillomavirus vaccine*[Title/Abstract]) OR (Human Papillomavirus immun*[Title/Abstract]) OR (Cer-var-ix[Title/Abstract

Search terms combination
#2.1 AND #5 AND (#4 OR #7a-j - added with OR)

Search results
The final search was conducted on 5 February 2024, and an updated search on 10 September 2024.

Database	First search	Second search	Total
**PubMed**	717	48	765
**The Cochrane Library**	876	33	909
**EMBASE**	423	42	465
**Web of Science**	411	29	440
**Scopus**	901	N/A	901

Table S3.1 : Study characteristics

Study	Population (n)	Design	Country	No. of centers	Intervention	Comparator	Outcome
Aydillo et al. 2024	128	Cohort Study	Spain	2	Influenza + COVID-19 vaccine	Influenza vaccine only	- Influenza SPR<br>- Influenza GMFR<br>- AE analysis
Baj et al. 2022	64	Cohort Study	Italy	1	Influenza + COVID-19 vaccine	COVID-19 vaccine only	- Covid GMFR
Choi et al. 2024	154	Non-randomised interventional trial	South Korea	1	Influenza + COVID-19 vaccine	Influenza vaccine only	- AE analysis
Izikson et al. 2022	296	RCT	United States	6	Influenza + COVID-19 vaccine	- Influenza vaccine only<br>- COVID-19 vaccine only	- Influenza SPR<br>- Influenza GMFR<br>- AE analysis
Gonen et al. 2023	649	Cohort Study	Israel	1	Influenza + COVID-19 vaccine	- Influenza vaccine only<br>- COVID-19 vaccine only	- AE analysis
Dulfer et al. 2023	154	RCT	The Netherlands	1	Influenza + COVID-19 vaccine	- Influenza vaccine + placebo<br>- COVID-19 vaccine + placebo	- AE analysis
Radner et al. 2023	781	Cohort Study	Austria	1	Influenza + COVID-19 vaccine	COVID-19 vaccine only	- Influenza SPR
Ramsay et al. 2023	248	RCT	Australia	1	Influenza + COVID-19 vaccine	- Influenza vaccine + placebo<br>- COVID-19 vaccine + placebo	- AE analysis
Shenyu et al. 2022	480	RCT	China	1	Influenza + COVID-19 vaccine	Sequential administration of vaccines	- Influenza SPR<br>- Influenza GMFR<br>- AE analysis
Toback et al. 2021	15187	RCT	United Kingdom	33	Influenza + COVID-19 vaccine	- COVID-19 vaccine only<br>- Influenza vaccine + placebo	- Influenza GMFR<br>- AE analysis
Wagenhäuser et al. 2023	1231	Non-randomised interventional trial	Germany	N/A	Influenza + COVID-19 vaccine	COVID-19 vaccine only	- AE analysis
Murdoch et al. 2023	1128	RCT	Australia; New Zealand	25	Influenza + COVID-19 vaccine	- COVID-19 vaccine only<br>- Influenza vaccine + placebo	- Influenza SPR<br>- Influenza GMFR<br>- AE analysis

Moss et al. 2023	54	Cohort Study	Israel	1	Influenza + COVID-19 vaccine	- COVID-19 vaccine only<br>- Influenza vaccine only	- Influenza GMFR
Hause et al. 2022	981099	Cohort Study	United States	N/A	Influenza + COVID-19 vaccine	- COVID-19 vaccine only	- AE analysis
Lazarus et al. 2021	679	RCT	United Kingdom	12	Influenza + COVID-19 vaccine	- COVID-19 + placebo	- Covid GMFR<br>- AE analysis
Chen et al. 2022	1152	RCT	China	2	Influenza + COVID-19 vaccine	- COVID-19 vaccine only<br>- Influenza vaccine only	- Influenza SPR<br>- Influenza GMFR<br>- Covid GMFR<br>- AE analysis
Moscara et al. 2023	1038	Cohort study	Italy	1	Influenza + COVID-19 vaccine	- COVID-19 vaccine only<br>- Influenza vaccine only	- AE analysis
Cannon et al. 2023	1791	RCT	United States	N/A	Influenza + pneumococcal vaccine	Sequential administration of vaccines	- Influenza GMFR
Frenck et al. 2012	1116	RCT	United States	34	Influenza + pneumococcal vaccine	- Influenza vaccine only	- Influenza GMFR
Nakashima et al. 2018	161	RCT	Japan	1	Influenza + pneumococcal vaccine	- Influenza vaccine only	- Influenza SPR<br>- AE analysis
Ofori-Anyinam et al. 2017	356	RCT	Belgium	6	Influenza + pneumococcal vaccine	- Influenza vaccine + placebo with sequential pneumococcal vaccine	- Influenza SPR<br>- AE analysis
Severance et al. 2022	1200	RCT	United States	45	Influenza + pneumococcal vaccine	- Influenza vaccine + placebo	- Influenza SPR<br>- Influenza GMFR
Thompson et al. 2019	882	RCT	United States	42	Influenza + pneumococcal vaccine	- Influenza vaccine + placebo	- Influenza GMFR
Schwarz et al. 2011	1190	RCT	Germany; The Netherlands; Belgium; Hungary	39	Influenza + pneumococcal vaccine	- Influenza vaccine + placebo<br>- Pneumococcal vaccine + placebo	- Influenza SPR<br>- Influenza GMFR<br>- AE analysis
Jansen et al. 2008	579	RCT	The Netherlands	N/A	Influenza + pneumococcal vaccine	- Influenza vaccine + placebo	- AE analysis
Song et al. 2015	224	RCT	South Korea	1	Influenza + pneumococcal vaccine	- Influenza vaccine only<br>- Pneumococcal vaccine only	- Influenza SPR<br>- AE analysis

Song et al. 2016	1237	RCT	South Korea	4	Influenza + pneumococcal vaccine	- Influenza vaccine only<br>- Pneumococcal vaccine only	- Influenza SPR<br>- AE analysis
Sočan et al. 2004	861	Cohort Study	Slovenia	4	Influenza + pneumococcal vaccine	- Pneumococcal vaccine only	- AE analysis
Honkanen et al. 1996	9336	Non-randomised interventional trial	Finland	N/A	Influenza + pneumococcal vaccine	- Influenza vaccine only	- AE analysis
DeStefano et al. 1982	126	RCT	United States	1	Influenza + pneumococcal vaccine	- Influenza vaccine + placebo	- AE analysis
Mufson et al. 1980	68	RCT	United States	N/A	Influenza + pneumococcal vaccine	- Influenza vaccine only	- AE analysis
Sadoff et al. 2021	180	RCT	United States	1	Influenza + RSV vaccine	- Influenza vaccine + placebo	- AE analysis
Widagdo et al. 2023	771	RCT	The Netherlands	N/A	Influenza + RSV vaccine	- Influenza vaccine + placebo	- Influenza SPR<br>- Influenza GMFR<br>- AE analysis
Chandler et al. 2024	976	RCT	New Zealand; Panama; South Africa	14	Influenza + RSV vaccine	- Influenza vaccine only	- Influenza SPR<br>- AE analysis
Athan et al. 2023	1403	RCT	Australia	31	Influenza + RSV vaccine	- Influenza vaccine + placebo<br>- sequential RSV-vaccine	- Influenza SPR<br>- Influenza GMFR<br>- AE analysis
Zimmermann et al. 2013	954	RCT	France; Germany	3	Influenza + RSV vaccine	Sequential administration of vaccines	- Influenza SPR<br>- AE analysis
McNeil et al. 2007	720	RCT	Canada	6	Influenza + DTP vaccine	- Influenza vaccine only	- AE analysis
Sukumaran L et al. 2015	36844	Cohort Study	United States	7	Influenza + DTP vaccine	Sequential administration of vaccines	- AE analysis
Glover et al. 2020	13758	Cohort Study	Australia	219	Influenza + DTP vaccine	- Influenza vaccine only<br>- DTP vaccine only	- AE analysis
Weston et al. 2009	1497	RCT	United States	12	Influenza + DTP vaccine	Sequential administration of vaccines	- Influenza SPR<br>- Influenza GMFR
Breiman et al. 2009	2503	RCT	Bangladesh; Chile; Colombia; Malaysia; Peru; Philippines; Thailand	N/A	Influenza + Polio vaccine	Polio vaccine + placebo	- Influenza GMFR<br>- AE analysis

Villanueva et al. 2022	2615	RCT	Australia	6	Influenza + BCG vaccine	- Influenza vaccine only	- AE analysis
Nolan et al. 2008	1251	RCT	United States	42	Influenza + MMR/Varicella vaccine	- Influenza vaccine only<br>- MMR/VAR vaccine only	- AE analysis
Lum et al. 2009	1233	RCT	Bangladesh; Belgium; Finland; Germany; Hong Kong; Lithuania; Malaysia; Mexico; Philippines; Poland; Singapore; South Korea; Thailand	32	Influenza + MMR vaccine	- MMR vaccine + placebo	- AE analysis
Nazareth et al. 2024	420	Cohort Study	United Kingdom; China; Australia; United States	1	Influenza + COVID-19 vaccine	Sequential administration of vaccines	- Influenza GMFR<br>- Covid GMFR
Zhu et al. 2024	480	RCT	China	1	Influenza + pneumococcal vaccine	Sequential administration of vaccines	- Influenza SPR<br>- Influenza GMFR
Clark et al. 2024	1045	RCT	Belgium; Finland; France; Spain; United Kingdom	37	Influenza + RSV vaccine	Sequential administration of vaccines	- Influenza SPR<br>- AE analysis
Buynak et al. 2024	1029	RCT	United States	48	Influenza + RSV vaccine	- Influenza vaccine only<br>- RSV vaccine only	- Influenza SPR<br>- Influenza GMFR<br>- AE analysis
Naficy et al. 2024	995	RCT	United States	47	Influenza + COVID-19 vaccine	Sequential administration of vaccines	- Influenza SPR<br>- Influenza GMFR<br>- Covid GMFR<br>- AE analysis
Huang et al. 2024	299	RCT	China	1	Influenza + COVID-19 vaccine	Sequential administration of vaccines	- Influenza SPR<br>- Influenza GMFR<br>- AE analysis
Chime et al. 2024	1586	RCT	Canada; Finland; South Korea; Spain; United States	N/A	Influenza + RSV vaccine	- Influenza vaccine + placebo<br>- RSV vaccine only	- AE analysis
Bai et al. 2024	636	RCT	China	1	- Influenza + COVID-19 vaccine<br>- Influenza + COVID-19 vaccine	Influenza vaccine only	- Influenza SPR

Table S3.2 : Definitions

Subgroup / Outcome	Definition
GMFR	Geometric mean fold rise in antigen-specific antibody titres from baseline (pre-vaccination) to follow-up (post-vaccination) measurements, comparing co-administration versus non-concomitant administration
Seroprotection	Proportion of participants achieving haemagglutination inhibition (HI) antibody titres ≥1:40 post-vaccination regardless of baseline levels

GMT	Geometrical mean titre (GMFR was calculated using GMT values at baseline and follow-up measurements in studies that did not directly report GMFR)
Young age group	Upper age range <65 years or mean/median age +1 SD < 60 years
Aged group	Lower age range >65 years or mean/median age -1 SD >70 years
Other group	Intermediate or missing data.
Female group	Defined as female overrepresented (female ratio >55%)
Male group	Defined as male overrepresented (female participant ratio <45%)
Sex neutral	Defined as in between male and female group representation
Serious adverse events	Defined as grade 3 or higher according to Common Terminology Criteria for Adverse Events (CTCAE)

Table S3.3 Inclusion and exclusion criteria for study selection and quantitative synthesis

Domain	Inclusion criteria	Exclusion criteria
**Population**	Human participants of any age or clinical background: adults, paediatric populations, pregnant women, immunocompromised individuals	Animal or other non-human studies
**Language**	Full-text articles published in English or German	Articles published in languages other than English or German
**Publication status**	Published primary research reports with sufficient information for eligibility assessment and data extraction	Unpublished studies; conference abstracts; full text unavailable for eligibility assessment
**Study design**	Randomised controlled trials and comparative non-randomised studies, including cohort, case-control, and cross-sectional studies	Non-comparative clinical reports, case reports, case series, editorials, reviews, commentaries, protocols without results, and other non-primary research articles
**Sample size**	≥ 10 participants	≤ 10 participants
**Intervention**	Same-day co-administration of a seasonal influenza vaccine with COVID-19 or another routinely recommended vaccine. Same-day administration was defined as administration within 24 hours.	No concomitant vaccination within predefined 24-hour window
**Eligible co-administered vaccines**	Seasonal influenza vaccine co-administered with: COVID-19, respiratory syncytial virus (RSV), pneumococcal, diphtheria–tetanus–pertussis (DTP), poliomyelitis, measles–mumps–rubella (MMR), or Bacillus Calmette–Guérin vaccines (BCG)	Vaccine combinations outside the predefined review scope or studies not involving seasonal influenza vaccination
**Comparator**	sequential administration of the same vaccines on different days; administration of the influenza vaccine alone; administration of the co-administered vaccine alone; or administration of one component vaccine with placebo/saline control	No comparator group
**Eligible outcomes**	At least one predefined immunogenicity endpoint or at least one post-vaccination safety endpoint following vaccine co-administration	No eligible immunogenicity or post-vaccination safety outcome

**Immunogenicity outcomes**	Eligible measures included geometric mean titres, geometric mean fold rise, seroprotection rate, or other antibody-response data suitable for the predefined analyses	No Immunogenicity data
**Timing of immunogenicity assessment**	Prespecified early post-vaccination assessment occurring 21–35 days after the relevant vaccine dose. When multiple eligible assessments were available, the time point closest to day 28–30 was selected	Immunogenicity measurements outside the 21–35-day
**Safety outcomes**	Solicited adverse events, unsolicited adverse events, severe adverse events, serious adverse events, or other predefined post-vaccination safety outcomes	Studies reporting no post-vaccination safety data and no eligible immunogenicity endpoint
**COVID-19 immunogenicity analysis**	Studies evaluating same-day influenza and COVID-19 vaccine co-administration, including an eligible non-concomitant comparator for the COVID-19 vaccine response, and reporting COVID-19 GMT or GMFR data suitable for estimating relative antibody response	No eligible comparator for COVID-19 immunogenicity
**Duplicate reports**	Where multiple reports described the same study population, the most complete or relevant report was retained, with companion reports used where necessary to supplement data	Duplicate records or duplicate publications not contributing additional relevant information

Table S4: Exclusion Table

Reasons for exclusion

ID	Reason	No. of studies
1	Wrong publication type/study design, patient number <10	7
2	Full text not accessible for eligibility assessment	10
3	Wrong Language	1
4	Wrong intervention	7
5	Comparator group missing/wrong outcome	17
6	other	9
7	duplicate	18
8	not published	22

List of studies excluded at full-text screening stage

Author/trial number	Year	Title	Exclusion reason
ACTRN12621001063808	2021	A randomised control trial to evaluate the reactions of co-administration of influenza and COVID-19 vaccines	7
Baos et al.	2024	Delivering COVID-19 vaccine trials at speed: the implementation of a phase IV UK multi-centre randomised controlled trial to determine safety and immunogenicity of COVID-19 vaccines co-administered with seasonal influenza vaccines (ComFluCOV)	6
Chandler et al.	2022	Immunogenicity, reactogenicity and safety of a Respiratory Syncytial Virus prefusion F (RSVPreF3) candidate vaccine co-administered with the seasonal quadrivalent influenza vaccine in older adults	2
Chang et al.	2012	Additive benefits of pneumococcal and influenza vaccines among elderly persons aged 75 years or older in Taiwan--a representative population-based comparative study	4
Choi et al.	2021	Detecting signals for the vaccine-vaccine interaction between pneumococcal vaccines and influenza vaccines using the Korea adverse event reporting system (KAERS)	2
Comeaux et al.	2019	Safety and immunogenicity of a seasonal influenza vaccine and AD26.RSV.pref vaccine with and without co-administration: a randomized, double-blind, placebo-controlled phase 2A study in adults aged ≥ 60 years	2
Domínguez et al.	2013	Effectiveness of vaccination with 23-valent pneumococcal polysaccharide vaccine in preventing hospitalization with laboratory confirmed influenza during the 2009-2010 and 2010-2011 seasons	4
Domnich et al.	2022	Acceptance of COVID-19 and Influenza Vaccine Co-Administration: Insights from a Representative Italian Survey	5
Duffy et al.	2013	Risk of febrile seizure following inactivated influenza vaccine and concomitant vaccines	2
EUCTR2007-002326-29-BE	2007	A Phase 3, Randomized, Double-blind Trial to Evaluate the Safety, Tolerability, and Immunogenicity of a 13-Valent Pneumococcal Conjugate Vaccine When Administered Concomitantly With Trivalent Inactivated Influenza Vaccine in Healthy Adults 65 Years of Age or Older Who Are Naive to 23-Valent Pneumococcal Polysaccharide Vaccine	8
EUCTR2010-021068-13-FR	2010	An open-label, randomised, multicentre study to evaluate the immunogenicity and safety of a booster dose of diphtheria, tetanus, acellular pertussis and inactivated poliomyelitis adsorbed vaccine (REPEVAX) administered concomitantly versus non-concomitantly with an influenza vaccine (VAXIGRIP) to subjects of 60 years of age and older	7
EUCTR2014-001118-24-BE	2014	Evaluation of the immunogenicity and safety of GlaxoSmithKline (GSK) Biologicals' Quadrivalent Influenza Vaccine Influsplit™ Tetra (Fluarix™ Tetra) (GSK2321138A) when co-administered with Pneumovax™ 23 in adults 50 years of age and older	8
EUCTR2021-000357-26-FI	2021	A Phase III study to assess the lot-to-lot consistency of GSK's investigational RSV maternal vaccine and the immune response and safety of RSV maternal vaccine when given alone or co-administered with GSK's influenza D-QIV vaccine in healthy non-pregnant women	8
EUCTR2021-003953-43-BE	2021	COVID-19: a Phase 3 Study to Evaluate Safety, Reactogenicity, and Immunogenicity of Co-administration of Ad26.COV2.S and Influenza Vaccines in Healthy Adults 18 Years of Age and Older	8
EUCTR2022-000623-21-BE	2022	A study on the immune response and safety of a vaccine against respiratory syncytial virus (RSV) when given alone or co-administered with an adjuvanted vaccine against influenza in adults aged 65 years and above	8
Fletcher et al.	1996	The simultaneous administration of influenza vaccine and 23-valent pneumococcal polysaccharide vaccine in patients with chronic respiratory disease	2
Gavazzi et al.	2012	Immunogenicity and safety of concomitant versus sequential administration of diphtheria/tetanus/pertussis/poliomyelitis vaccine (Tdap-IPV) and trivalent influenza vaccine in elderly individuals	2

Ghazy et al.	2023	The Effect of Combining the COVID-19 Vaccine with the Seasonal Influenza Vaccine on Reducing COVID-19 Vaccine Rejection Among Libyans	5
He et al.	2018	Safety of influenza vaccine and 23-valent pneumococcal polysaccharide vaccine immunized alone or in combination in the elderly	3
Hussein et al.	2023	Combining COVID-19 and seasonal influenza vaccines together to increase the acceptance of newly developed vaccines in the Eastern Mediterranean Region: a cross-sectional study	6
ISRCTN14391248	2021	Combining influenza and COVID-19 vaccination (ComFluCOV) study	7
Kaine et al.	2007	Immune responses following administration of influenza and pneumococcal vaccines to patients with rheumatoid arthritis receiving adalimumab	4
Kenigsberg et al.	2023	Simultaneous administration of mRNA COVID-19 bivalent booster and influenza vaccines	6
Nakashima et al.	2016	Interim analysis of a randomized, open-label, parallel design study to compare the immunogenicity of simultaneous administration versus sequential administration of quadrivalent influenza vaccine and 23-valent polysaccharide pneumococcal vaccine	2
Nakashima et al.	2017	A randomized, open-label, parallel design study to compare the immunogenicity of simultaneous administration versus sequential administration of quadrivalent influenza vaccine and 23-valent pneumococcal polysaccharide vaccine in the elderly	2
NCT00192166	2005	Trial to Assess Safety, Efficacy, Tolerability and Immunogenicity of Influenza Virus Vaccine, Liquid Formulation (CAIV-T), Administered Concomitantly With a Combination Live, Attenuated, Mumps, Measles, and Rubella Vaccine in Healthy Children Aged 11 - 24 Months	7
NCT00192491	2005	Trial to Assess Safety, Tolerability, and Immunogenicity of Influenza Virus Vaccine, Trivalent, Types A & B, Live Cold-Adapted (FluMist) and Measles, Mumps, Rubella, and Varicella Vaccines Administered Concurrently to Healthy Children	7
NCT00385255	2006	Immunogenicity, Safety of GSKs Tdap Vaccine Boostrix When Coadministered With GSKs Influenza Vaccine Fluarix in Adults	7
NCT00579345	2007	Second Extension Study to Evaluate Safety and Tolerability of Influenza Vaccines in Adults and Elderly, and to Evaluate Immunogenicity and Concomitant Vaccination With Pneumococcal Vaccine in a Subgroup	7
NCT01249183	2010	Safety and Immunogenicity Study of Concomitant Versus Non Concomitant Administration of a Diphtheria, Tetanus, Pertussis and Poliomyelitis Vaccine (REPEVAX) and Influenza Vaccine (VAXIGRIP) in Subjects Aged From 60 Years Old	8
NCT02124161	2014	Concomitant Administration of 13-valent Pneumococcal Conjugate Vaccine (13vPnC) With Influenza Vaccine in 23-valent Pneumococcal Polysaccharide (23vPS) Pre-vaccinated Adults	7
NCT02218697	2014	Evaluation of the Immunogenicity and Safety of GlaxoSmithKline (GSK) Biologicals' Quadrivalent Influenza Vaccine Influsplit™ Tetra (Fluarix™ Tetra) (GSK2321138A) When Co-administered With Pneumovax™ 23 in Adults 50 Years of Age and Older	7
NCT02225327	2014	MF59-adjuvanted Influenza Vaccine and 23-valent Pneumococcal Polysaccharide Vaccine	7
NCT02582047	2015	Influenza Vaccine and Pneumococcal Vaccine	8
NCT02592486	2015	The Immunogenicity of Simultaneous Administration of Quadrivalent Influenza Vaccine and 23-valent Pneumococcal Vaccine	7
NCT02765126	2016	Heterologous Effect of Diptheria, Tetanus, Acellular Pertussis Vaccination on Influenza Challenge in the Elderly	8
NCT02783170	2016	Safety and Immunogenicity of Simultaneous Tdap and IIV in Pregnant Women	2
NCT03339713	2017	A Study to Evaluate the Safety and Immunogenicity of Seasonal Influenza Vaccine and an Adenovirus Serotype 26- Based Vaccine Encoding for the Respiratory Syncytial Virus Pre-fusion F Protein (Ad26.RSV.preF), With and Without Co-administration, in Adults Aged 60 Years and Older in Stable Health	7
NCT03615482	2018	A Study to Evaluate the Safety, Tolerability, and Immunogenicity of V114 When Administered Concomitantly With Influenza Vaccine in Healthy Adults 50 Years of Age or Older (V114-021/PNEU-FLU)	7

NCT04526574	2020	Safety and Immunogenicity of 20vPnC Coadministered With SIIV in Adults ≥65 Years of Age	7
NCT04559204	2020	Evaluation of Safety and Immunogenicity of Combined Immunization of PPV23 and IIV4	6
NCT04801888	2021	Study on Combined Vaccination With SARS-CoV-2 Inactivated Vaccine and Quadrivalent Influenza Vaccine	7
NCT04841577	2021	A Study on the Immune Response and Safety Elicited by a Vaccine Against Respiratory Syncytial Virus (RSV) When Given Alone and Together With a Vaccine Against Influenza in Adults Aged 60 Years and Above	7
NCT04848467	2021	COVID-19: a Trial Studying the SARS-CoV-2 mRNA Vaccine CVnCoV to Learn About the Immune Response, the Safety, and the Degree of Typical Vaccination Reactions When CVnCoV is Given at the Same Time as a Flu Vaccine Compared to When the Vaccines Are Separately Given in Adults 60 Years of Age and Older	8
NCT05045144	2021	A Phase III Study to Assess the Lot-to-lot Consistency of GSK's Investigational RSV Maternal Vaccine and the Immune Response and Safety of RSV Maternal Vaccine When Given Alone or Co-administered With GSK's Influenza D-QIV Vaccine in Healthy Non-pregnant Women	8
NCT05071313	2021	A Study of an Ad26.RSV.preF-based Vaccine and High-dose Seasonal Influenza Vaccine, With and Without Coadministration, in Adults Aged 65 Years and Older	8
NCT05091307	2021	A Study of Ad26.COV2.S and Influenza Vaccines in Healthy Adults	8
NCT05107375	2021	Clinical Study of Recombinant Novel Coronavirus（COVID-19） Vaccine (CHO Cell) Combined With Influenza Vaccine	8
NCT05499351	2022	A Study of Booster Immunization With COVID-19 Vaccine,Inactivated Co -Administration With Influenza Vaccine and Pneumococcal Polysaccharide Vaccine	8
NCT05559476	2022	A Study on the Immune Response and Safety Elicited by a Vaccine Against Respiratory Syncytial Virus (RSV) When Given Alone and Together With a Vaccine Against Influenza in Adults Aged 65 Years and Above	8
NCT05568797	2022	A Study on the Immune Response and Safety of a Vaccine Against Respiratory Syncytial Virus (RSV) When Given Alone or Co-administered With an Adjuvanted Vaccine Against Influenza in Adults Aged 65 Years and Above	8
NCT05981846	2023	A Phase II Trial to Evaluate the Safety and Immunogenicity of BIMERVAX® When Coadministered With Seasonal Influenza Vaccine (SIIV) in Adults Older Than 65 Years of Age Fully Vaccinated Against COVID-19	8
NCT06020118	2023	Comparative Immunogenicity of Concomitant vs Sequential mRNA COVID-19 and Influenza Vaccinations	8
NCT06060457	2023	A Study to Evaluate the Safety and Immune Response of mRNA-1345, a Vaccine Targeting Respiratory Syncytial Virus (RSV), When Co-administered With a Fluzone HD, in Adults ≥65 Years of Age	8
NCT06091410	2023	Immunogenicity of Concomitant Administration of COVID-19 Vaccines With Influenza Vaccines	8
Pascucci et al.	2023	Co-administration of SARS-CoV-2 and influenza vaccines in healthcare workers: Results of two vaccination campaigns in a large teaching hospital in Rome	5
Schönbeck et al.	2005	Rationale and design of the prevention of respiratory infections and management in children (PRIMAKid) study: a randomized controlled trial on the effectiveness and costs of combined influenza and pneumococcal vaccination in pre-school children with recurrent respiratory tract infections	1
Seo et al.	2017	Comparison of immunogenicity and safety of an influenza vaccine administered concomitantly with a 13-valent pneumococcal conjugate vaccine or 23-valent polysaccharide pneumococcal vaccine in the elderly	5
Shen et al.	2023	Pilot Study for Immunogenicity of SARS-CoV-2 Vaccine with Seasonal Influenza and Pertussis Vaccines in Pregnant Women	4
Stefanizzi et al.	2022	Acceptability of the third dose of anti-SARS-CoV-2 vaccine co-administered with influenza vaccine: preliminary data in a sample of Italian HCWs	1
Stefanizzi et al.	2022	Immunogenicity of third dose of anti-SARS-CoV-2 vaccine co-administered with influenza vaccine: An open question	5

Buynder et al.	2013	Increased reactions to pediatric influenza vaccination following concomitant pneumococcal vaccination	4
Vilella et al.	2005	Reactogenicity profile of a combined hepatitis A and B vaccine in clinical practice: A naturalistic study in adult travellers	4
Wagenhäuser et al.	2022	Immunogenicity and safety of coadministration of COVID-19 and influenza vaccination among healthcare workers	7
Wang et al.	2019	Immunogenicity and safety of co-immunization with 23-valent pneumococcal polysaccharide vaccine and influenza virus split vaccine for children aged 3-7 years	2
Winthrop et al.	2013	Evaluation of influenza and pneumococcal vaccine responses in rheumatoid arthritis patients using tofacitinib	1
Baker et al.	2020	The risk of febrile seizures following influenza and 13-valent pneumococcal conjugate vaccines	6
Carlson et al.	1979	Pneumococcal Vaccine: Dose, Revaccination, and Coadministration with Influenza Vaccine	7
Falsey et al.	2008	Comparison of the safety and immunogenicity of 2 respiratory syncytial virus (rsv) vaccines--nonadjuvanted vaccine or vaccine adjuvanted with alum--given concomitantly with influenza vaccine to high-risk elderly individuals	4
Falsey et al.	2022	Phase 1/2 Randomized Study of the Immunogenicity, Safety, and Tolerability of a Respiratory Syncytial Virus Prefusion F Vaccine in Adults With Concomitant Inactivated Influenza Vaccine	1
Grilli et al.	1997	Simultaneous influenza and pneumococcal vaccination in elderly individuals	6
Hilleman et al.	1981	Streptococcus pneumoniae polysaccharide vaccine: Age and dose responses, safety, persistence of antibody, revaccination, and simultaneous administration of pneumococcal and influenza vaccines	6
NCT01709019	2012	RSV-F Vaccine and Influenza Vaccine Co-Administration Study in the Elderly	1
Schwarz et al.	2013	Assessment of functional antibacterial opsonophagocytic antibodies elicited by 13-valent pneumococcal conjugate vaccine administered concomitantly with trivalent influenza vaccine in a randomized clinical trial in adults aged ≥65 years	5
Szymczakiewicz-Multanowska et al.	2012	Safety assessment and immunogenicity of a cell-culture-derived influenza vaccine in adults and elderly subjects over three successive influenza seasons	6
Yilmaz et al.	2023	The Effect of Pneumococcal, Influenza, and COVID-19 Vaccinations on COVID-19 Hospitalization and Progression in People over 65 Years Old Living in Nursing Homes	6
CTIS2023-506189-29-00	2023	A phase II, randomized, double-blind, multi-centre trial to evaluate the safety and immunogenicity of BIMERVAX® when coadministered with seasonal surface antigen, inactivated, adjuvanted influenza vaccine (SIIV) in adults older than 65 years of age fully vaccinated against COVID-19	8
Ali et al.	2024	Co-administration of the adjuvanted recombinant zoster vaccine with other adult vaccines: An overview	1
Harris et al.	2024	COVID-19 and Influenza Vaccine Coadministration Among Older U.S. Adults	5
Baum et al.	2024	Detection of SARS-CoV-2-specific mucosal antibodies in saliva following concomitant COVID-19 and influenza vaccination in the ComFluCOV trial	5
Handabile et al.	2024	Immunogenicity and protective efficacy of a co-formulated two-in-one inactivated whole virus particle COVID-19/influenza vaccine	1
NCT06279871	2024	Immunogenicity and Safety Study of Self-amplifying mRNA COVID-19 Vaccine Administered With Influenza Vaccines in Adults	8
Pattinson et al.	2024	Ipsilateral and contralateral coadministration of influenza and COVID-19 vaccines produce similar antibody responses	5
Costantino et al.	2024	Real-life experience on COVID-19 and seasonal influenza vaccines co-administration in the vaccination hub of the University Hospital of Palermo, Italy	5
Moro et al.	2024	Safety of Simultaneous Administration of Bivalent mRNA COVID-19 and Influenza Vaccines in the Vaccine Adverse Event Reporting System (VAERS)	5

Sansone et al.	2024	Simultaneous vaccination against seasonal influenza and COVID-19 among the target population in Italy	5
Biegus et al.	2024	The early safety profile of simultaneous vaccination against influenza and Respiratory Syncytial Virus (RSV) in patients with high-risk heart failure	5
Russo et al.	2022	First SARS-CoV-2 vaccine booster and influenza vaccination: risk assessment of COVID-19 hospitalisation and death	5
McGrath et al.	2023	Estimated Effectiveness of Coadministration of the BNT162b2 BA.4/5 COVID-19 Vaccine With Influenza Vaccine	5
Won et al.	2024	Safety of concomitant administration of 23-valent polysaccharide pneumococcal vaccine and influenza vaccine among the elderly	5
Chan et al.	2012	Prevention of Mortality and Pneumonia Among Nursing Home Older Adults by Dual Pneumococcal and Seasonal Influenza Vaccination During a Pandemic Caused by Novel Pandemic Influenza A (H1N1)	5

Table S5: Certainty of evidence

S5.1 Immunogenicity outcomes in influenza vaccine group

Grading of recommendations, assessment, development and evaluations (GRADE) of immunogenicity outcomes in influenza vaccine group. The assessments were performed using GRADEpro (GRADEpro Guideline Development Tool, McMaster University).

Outcome	Certainty assessment							№ of patients		Effect		Certainty	Importance
	№ of studies	Study design	Risk of bias	Inconsistency	Indirectness	Imprecision	Other considerations	Coadmin.	Non concomitant admin.	Relative (95% CI)	Absolute (95% CI)		
**GMFR** A/H1N1	25[a,b]	RCTs	not serious[c]	very serious[d]	not serious[e]	not serious[f]	none	9949	9650	-	RoM 1.02 (0.95 to 1.1)	⊕⊕◯◯ Low[c,d,e,f]	Critical
**GMFR** A/H3N2	25[a,b]	RCTs	not serious[c]	very serious[d]	not serious[e]	not serious[f]	none	9955	9656	-	RoM 1.05 (0.97 to 1.13)	⊕⊕◯◯ Low[c,d,e,f]	Critical
**GMFR** Influenza B	15[b,g]	RCTs	not serious[c]	very serious[d]	not serious[e]	not serious[f]	none	6989	6987	-	RoM 1.01 (0.97 to 1.05)	⊕⊕◯◯ Low[c,d,e,f]	Critical
**SPR** A/H1N1	22[b,h]	RCTs	not serious[c]	not serious[i]	not serious[e]	not serious[j]	none	8236	7518	RR 1.00 (0.99 to 1.01)	-	⊕⊕⊕⊕ High[c,e,i,j]	Critical
**SPR** A/H3N2	22[b,k]	RCTs	not serious[c]	serious[l]	not serious[e]	not serious[j]	none	8225	7506	RR 1 (1 to 1)	-	⊕⊕⊕◯ Moderate[c,e,j,l]	Critical
**SPR** Influenza B	16[b,m]	RCTs	not serious[c]	not serious[n]	not serious[e]	not serious[j]	none	5358	4725	RR 1 (1 to 1)	-	⊕⊕⊕⊕ High[c,e,j,n]	Critical

Explanations
a. 22 RCTs, 3 observational studies

b. Evidence includes both randomized controlled trials and observational studies. Certainty of evidence was rated conservatively due to inclusion of non-randomized studies. For RCT-only subgroup analyses, see Supplementary Material

c. Most evidence came from RCTs with low risk or some concerns; no downgrades applied.

d. Downgraded 2 for very serious inconsistency: substantial heterogeneity ($I^2 > 70\%$), effect estimates varied widely in both magnitude and direction

e. Populations, interventions, comparators, and outcomes (PICO) were generally consistent with the review question

f. No established MID exists for GMFR, therefore imprecision was not assessed. Certainty was instead rated down for indirectness as immunogenicity is a surrogate outcome

g. 14 RCTs, 1 observational studies

h. 20 RCTs, 2 observational studies

i. No downgrade: low-to-moderate heterogeneity ($I^2 = 35\%$), effect estimates were consistent in direction across studies

j. Minimal important difference (MID) prespecified as a 10% absolute difference in SPR based on regulatory practice.

k. 20 RCTs, 2 observational studies

l. Downgraded 1: moderate heterogeneity ($I^2 = 49.9\%$), effect estimates were consistent in direction across studies

m. 14 RCTs, 2 observational studies

n. No downgrade: low-to-moderate heterogeneity ($I^2 = 9.4\%$), effect estimates were consistent in direction across studies

S5.2 Immunogenicity outcomes in COVID-19 vaccine group.
Grading of recommendations, assessment, development and evaluations (GRADE) of immunogenicity outcomes in COVID-19 vaccine group.

Outcome	Certainty assessment							№ of patients		Effect		Certainty	Importance
	№ of studies	Study design	Risk of bias	Inconsistency	Indirectness	Imprecision	Other considerations	Coadmin.	Non concomitant admin.	Relative (95% CI)	Absolute (95% CI)		
**GMFR**	7[a]	RCTs	not serious[b]	serious[c]	not serious[d]	not serious[e]	none	1757	1946	-	RoM 0.84 (0.74 to 0.95)	⨁⨁⨁◯ Moderate [b,c,d,e]	Critical

Explanations

a. Evidence includes both randomized controlled trials (5 RCTs) and observational studies (2 OBS). Certainty of evidence was rated conservatively due to inclusion of non-randomized studies. For RCT-only subgroup analyses, see Supplementary Material

b. Most evidence came from RCTs with low risk or some concerns; no downgrades applied.

c. Downgraded 1 for inconsistency: very high heterogeneity ($I^2 = 99.7\%$), effect estimates were consistent in direction across studies

d. Populations, interventions, comparators, and outcomes (PICO) were generally consistent with the review question

e. No established MID exists for GMFR, therefore imprecision was not assessed. Certainty was instead rated down for indirectness as immunogenicity is a surrogate outcome

S5.3 Adverse events incidence

Grading of recommendations, assessment, development and evaluations (GRADE) of adverse events incidence

Outcome	Certainty assessment							№ of patients		Effect		Certainty	Importance
	№ of studies	Study design	Risk of bias	Inconsistency	Indirectness	Imprecision	Other considerations	Coadmin.	Non concomitant admin.	Relative (95% CI)	Absolute (95% CI)		
**Any**	17	RCTs	not serious	very serious[a]	not serious[b]	not serious[c]	none	8436	20604	RR 1.05 (0.93 to 1.18)	-	⊕⊕◯◯ Low[a,b,c]	Critical
**Serious AE**	26	RCTs	not serious	very serious[a]	not serious[b]	serious[c]	none	196853	1259332	RR 1.41 (1.07 to 1.86)	-	⊕◯◯◯ Very low[a,b,c]	Critical
**Arthralgia**	19	RCTs	not serious	very serious[a]	not serious[b]	serious[c]	none	99641	896955	RR 1.31 (1.10 to 1.57)	-	⊕◯◯◯ Very low[a,b,c]	Important
**Chills**	15	RCTs	not serious	very serious[a]	not serious[b]	serious[c]	none	96914	893286	RR 1.44 (1.07 to 1.94)	-	⊕◯◯◯ Very low[a,b,c]	Important
**Cough**	6	RCTs	not serious	not serious[d]	not serious[b]	not serious[c]	none	3802	3397	RR 0.98 (0.91 to 1.05)	-	⊕⊕⊕⊕ High[b,c,d]	Not Important
**Diarrhea**	10	RCTs	not serious	serious[e]	not serious[b]	serious[c]	none	97160	905206	RR 1.10 (0.87 to 1.39)	-	⊕⊕◯◯ Low[b,c,e]	Important
**Fatigue**	26	RCTs	not serious	serious[e]	not serious[b]	serious[c]	none	102370	901006	RR 1.31 (1.13 to 1.52)	-	⊕⊕◯◯ Low[b,c,e]	Important
**Fever**	33	RCTs	not serious	serious[e]	not serious[b]	serious[c]	none	108946	915717	RR 1.35 (1.11 to 1.63)	-	⊕⊕◯◯ Low[b,c,e]	Important
**Gastrointestinal**	3	RCTs	not serious	not serious[d]	not serious[b]	serious[c]	none	1256	978	RR 0.85 (0.57 to 1.28)	-	⊕⊕⊕◯ Moderate[b,c,d]	Important
**Headache**	30	RCTs	not serious	serious[e]	not serious[b]	serious[c]	none	106476	913582	RR 1.26 (1.07 to 1.47)	-	⊕⊕◯◯ Low[b,c,e]	Important
**Malaise**	8	RCTs	not serious	not serious[f]	not serious[b]	not serious[c]	none	3071	3128	RR 1.08 (0.96 to 1.21)	-	⊕⊕⊕⊕ High[b,c,f]	Important
**Myalgia**	27	RCTs	not serious	serious[e]	not serious[b]	serious[c]	none	104040	900150	RR 1.27 (1.10 to 1.47)	-	⊕⊕◯◯ Low[b,c,e]	Important

**Nausea**	13	RCTs	not serious	very serious[a]	not serious[b]	serious[c]	none	96823	894911	RR 1.25 (0.90 to 1.73)	-	⊕○○○ Very low[a,b,c]	Important
**Rash**	3	RCTs	not serious	very serious[a]	not serious[b]	serious[c]	none	93147	890024	RR 0.71 (0.14 to 3.53)	-	⊕○○○ Very low[a,b,c]	Important
**Vomiting**	13	RCTs	not serious	very serious[a]	not serious[b]	serious[c]	none	101913	908431	RR 0.90 (0.65 to 1.25)	-	⊕○○○ Very low[a,b,c]	Important

Explanations

a. Downgraded 2 for inconsistency: high heterogeneity ($I^2 > 70\%$), effect estimates varied in direction and magnitude across studies

b. Populations, interventions, comparators, and outcomes (PICO) were generally consistent with the review question

c. Minimal Important Difference (MID) was a priori set to RR = 1.25 for adverse events

d. No downgrade: low-to-moderate heterogeneity ($I^2 <35\%$), effect estimates were consistent in direction across studies

e. Downgraded 1 for inconsistency: high heterogeneity ($I^2 >70\%$), effect estimates were mostly consistent in direction across studies

f. No downgrade: low-to-moderate heterogeneity ($I^2 = 42.6\%$), effect estimates were consistent in direction across studies

Table S6a: Risk of bias assessment – ROB2

	D1	D2	D3	D4	D5	Overall
Mufson et al. 1980	−	+	+	−	−	−
DeStefano et al.l 1982	−	+	+	+	+	−
McNeil et al. 2007	−	+	+	+	+	−
Jansen et al. 2008	−	+	+	+	−	−
Nolan et al. 2008	+	+	+	+	+	+
Weston et al. 2009	−	+	+	×	+	×
Breiman et al. 2009	+	+	+	+	+	+
Lum et al. 2009	+	+	+	+	+	+
Schwarz et al. 2011	+	+	+	+	+	+
Frenck et al. 2012	−	+	+	+	+	−
Zimmermann et al. 2013	−	+	+	+	+	−
Song et al. 2015	−	−	+	×	+	×
Song et al. 2016	+	−	+	−	+	−
Ofori-Anyinam et al 2017	+	+	+	+	+	+
Nakashima et al. 2018	+	+	+	−	+	−
Thompson et al. 2019	+	+	+	+	+	+
Chandler et al. 2020	+	−	+	−	+	−
Toback et al. 2021	+	+	+	+	+	+
Lazarus et al. 2021	+	+	+	+	−	−
Severance et al. 2021	+	+	+	+	+	+
Sadoff et al. 2021	+	−	+	+	+	−
Izikson et al. 2022	+	+	+	+	+	+
Shenyu et al. 2022	+	+	+	+	+	−
Chen et al. 2022	+	+	+	+	+	−
Villanueva et al. 2022	−	−	+	×	+	×
Dulfer et al. 2023	+	+	+	+	+	+
Ramsay et al. 2023	−	+	+	−	+	−
Murdoch et al. 2023	+	+	+	+	+	−
Cannon et al. 2023	+	+	+	+	+	−
Widagdo et al. 2023	+	+	+	+	+	+
Athan et al. 2023	+	+	+	+	+	+
Zhu et al. 2024	+	−	+	+	+	−
Clark et al. 2024	+	+	+	−	+	−
Naficy et al. 2024	+	+	+	−	+	−
Huang et al. 2024	+	+	+	−	+	−
Chime et al. 2024	+	+	+	+	+	+
Bai et al. 2024	+	+	+	+	+	+
Buynak et al 2024	+	+	+	−	+	−

Domain explanation:
D1: Bias arising from the randomization process
D2: Bias due to deviations from intended interventions
D3: Bias due to missing outcome data
D4: Bias in measurement of the outcome
D5: Bias in selection of the reported result

Proportions:

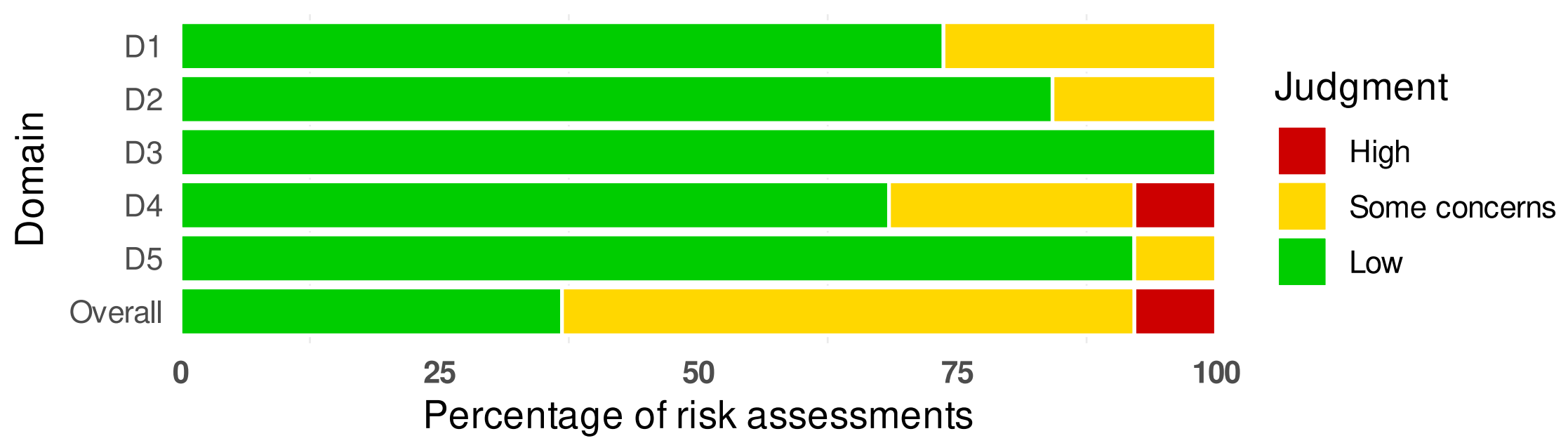

Table S6b: Risk of bias assessment – ROBINS-I

Study	D1	D2	D3	D4	D5	D6	D7	Overall
Choi et al. 2024	✖	⊖	⊕	⊖	⊕	⊖	⊕	✖
Aydillo et al. 2024	✖	⊖	⊕	⊕	⊖	⊖	⊖	✖
Baj et al. 2022	✖	⊖	⊕	⊕	⊖	⊖	⊖	✖
Gonen et al. 2023	⊖	✖	⊕	⊖	⊖	⊖	⊖	✖
Radner et al. 2023	⊖	⊖	⊕	⊕	⊖	⊖	⊕	⊖
Wagenhäuser et al. 2023	⊖	⊖	⊕	⊕	⊕	⊖	⊖	⊖
Moss et al. 2023	⊖	⊖	⊕	⊕	⊕	⊕	⊖	⊖
Hause et al. 2022	⊖	✖	⊖	⊕	⊖	✖	⊖	✖
Moscara et al. 2023	⊖	⊖	⊕	⊕	⊕	⊖	⊖	⊖
Sočan et al. 2004	⊖	✖	⊕	⊕	✖	⊖	⊖	✖
Sukumaran et al. 2015	⊖	⊖	⊕	⊕	⊕	⊖	⊖	⊖
Glover et al. 2020	⊖	⊖ v	⊕	⊕	✖	⊖	⊖	✖
Nazareth et al. 2024	⊖	⊖	⊕	⊕	⊖	⊕	⊖	⊖
Honkanen et al. 1996	⊖	⊖	⊕	⊕	⊕	⊖	⊖	⊖

ROBINS-I was applied at the study level using the most conservative applicable judgment across reported immunogenicity, safety/reactogenicity, and effectiveness outcomes.

Domain explanation:
D1: Bias due to confounding
D2: Bias in selection of participants into the study
D3: Bias in classification of interventions
D4: Bias due to deviations from intended interventions
D5: Bias due to missing data
D6: Bias in measurement of outcomes
D7: Bias in selection of the reported result

Supplementary figures

Primary outcome
Figure S1: GMFR Influenza H1N1

Study	CO Total	CO Mean	CO SD	SEQ Total	SEQ Mean	SEQ SD	ROM	95%-CI	Weight (common)	Weight (random)
Breiman et al. 2009	713	2.08	0.1980	721	1.80	0.1631	1.15	[1.14; 1.16]	4.0%	4.1%
Weston et al. 2009	711	7.30	0.4945	676	6.56	0.4390	1.11	[1.11; 1.12]	7.3%	4.1%
Schwarz et al. 2011	548	9.05	0.6144	546	8.53	0.5961	1.06	[1.05; 1.07]	5.5%	4.1%
Frenck et al. 2012	530	12.80	0.7908	531	13.60	0.8929	0.94	[0.93; 0.95]	6.2%	4.1%
Song et al. 2015	107	3.11	0.7724	56	2.97	0.6389	1.05	[0.97; 1.13]	0.1%	3.9%
Song et al. 2016	373	5.09	0.4549	382	4.53	0.3718	1.12	[1.11; 1.14]	2.5%	4.1%
Thompson et al. 2019	427	2.40	0.1199	430	2.20	0.1020	1.09	[1.08; 1.10]	8.8%	4.1%
Lazarus et al. 2021	340	1.61	0.1783	339	0.94	0.1146	1.72	[1.69; 1.75]	1.2%	4.1%
Toback et al. 2021	217	16.24	2.0946	214	13.57	2.0405	1.20	[1.17; 1.23]	0.5%	4.0%
Izikson et al. 2022	96	6.40	0.8674	86	8.10	1.1990	0.79	[0.76; 0.82]	0.2%	4.0%
Severance et al. 2022	569	4.20	0.2296	561	4.20	0.2551	1.00	[0.99; 1.01]	8.1%	4.1%
Shenyu et al. 2022	466	101.16	15.2447	228	76.02	10.3301	1.33	[1.30; 1.36]	0.7%	4.1%
Chen et al. 2022	322	9.38	1.0401	332	8.57	0.9055	1.09	[1.08; 1.11]	1.3%	4.1%
Athan et al. 2023	673	2.60	0.1225	673	3.30	0.1658	0.79	[0.78; 0.79]	13.6%	4.1%
Moss et al. 2023	18	1.41	0.4318	18	1.75	0.4761	0.81	[0.67; 0.98]	0.0%	3.3%
Murdoch et al. 2023	564	6.00	0.4847	564	6.40	0.5102	0.94	[0.93; 0.95]	4.2%	4.1%
Widagdo et al. 2023	337	4.00	0.2649	338	4.27	0.3208	0.94	[0.93; 0.95]	3.2%	4.1%
Cannon et al. 2023	895	1.80	0.0765	896	1.80	0.1020	1.00	[1.00; 1.00]	17.1%	4.1%
Aydillo et al. 2024	82	3.22	1.2572	46	4.63	2.0274	0.70	[0.60; 0.81]	0.0%	3.5%
Nazareth et al. 2024	234	8.00	0.7398	186	6.90	1.4031	1.16	[1.12; 1.20]	0.4%	4.0%
Zhu et al. 2024	156	45.66	6.4185	296	64.60	6.6506	0.71	[0.69; 0.72]	0.6%	4.1%
Clark et al. 2024	523	3.85	0.2424	522	3.79	0.2475	1.02	[1.01; 1.02]	6.1%	4.1%
Buynak et al 2024	450	5.57	0.4209	434	4.53	0.3240	1.23	[1.22; 1.24]	3.9%	4.1%
Naficy et al. 2024	462	9.38	0.6862	454	9.20	0.6480	1.02	[1.01; 1.03]	4.3%	4.1%
Huang et al. 2024	136	24.46	4.1031	121	24.03	4.3400	1.02	[0.98; 1.06]	0.2%	4.0%
**Common effect model**	**9949**			**9650**			**1.00**	**[1.00; 1.01]**	**100.0%**	**.**
**Random effects model**							**1.02**	**[0.95; 1.10]**	**.**	**100.0%**

Heterogeneity: $I^2 = 99.9\%$, $\tau^2 = 0.0376$, $p = 0$
Test for overall effect (random effects): $z = 0.59$ ($p = 0.5569$)

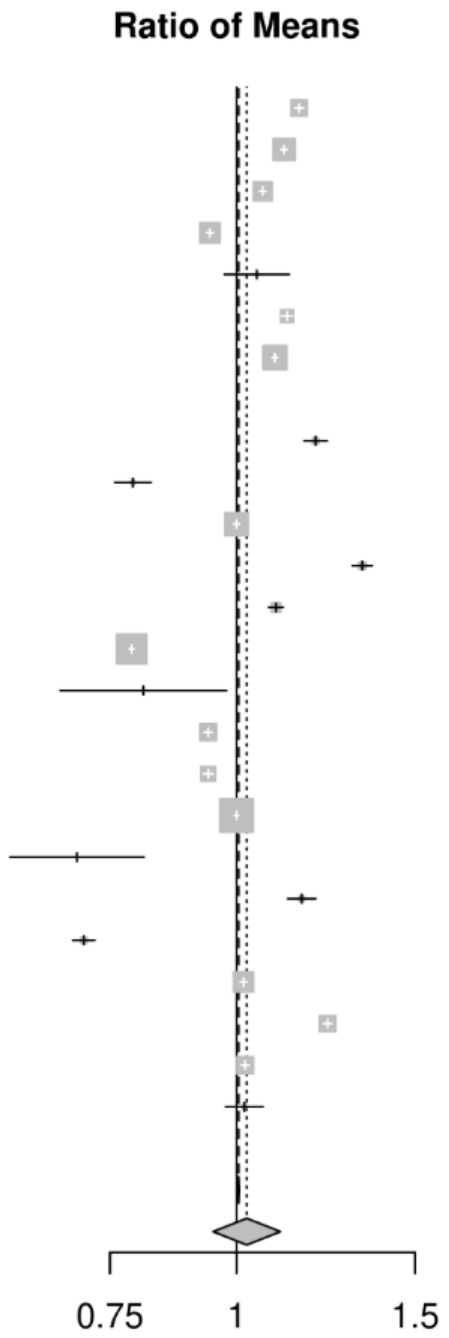

Figure S1: Influenza H1N1 Ratio of Means (ROM) comparing geometric mean fold rise (GMFR) between co-administration (CO) and non-concomitant groups (SEQ). Squares represent study-specific estimates, with square size proportional to study weight, and horizontal lines represent 95% confidence intervals. Diamonds represent overall estimates. ROM values greater than 1 indicate higher GMFR in the co-administration group.

Figure S2: GMFR Influenza H3N2

Study	CO Total	CO Mean	CO SD	SEQ Total	SEQ Mean	SEQ SD	ROM	95%-CI	Weight (common)	Weight (random)
Breiman et al. 2009	713	4.01	0.3894	721	3.86	0.3880	1.04	[1.03; 1.05]	3.0%	4.2%
Weston et al. 2009	711	13.61	0.9414	676	11.63	0.8348	1.17	[1.16; 1.18]	5.7%	4.2%
Schwarz et al. 2011	548	5.21	0.4324	546	6.33	0.5425	0.82	[0.81; 0.83]	3.2%	4.2%
Frenck et al. 2012	530	10.20	0.7143	531	9.70	0.7143	1.05	[1.04; 1.06]	4.2%	4.2%
Song et al. 2015	107	2.70	0.5389	56	2.27	0.4528	1.19	[1.12; 1.27]	0.1%	4.0%
Song et al. 2016	373	5.28	0.3939	382	3.80	0.2862	1.39	[1.37; 1.40]	2.8%	4.2%
Thompson et al. 2019	427	2.30	0.1071	430	2.40	0.1122	0.96	[0.95; 0.96]	8.1%	4.2%
Lazarus et al. 2021	340	1.47	0.1266	339	0.94	0.0947	1.56	[1.54; 1.58]	1.6%	4.2%
Toback et al. 2021	217	6.98	0.8612	214	6.16	0.8230	1.13	[1.11; 1.16]	0.5%	4.1%
Izikson et al. 2022	96	3.10	0.3061	86	3.50	0.4082	0.89	[0.86; 0.91]	0.3%	4.1%
Severance et al. 2022	569	2.20	0.1020	561	2.20	0.1020	1.00	[0.99; 1.01]	10.8%	4.2%
Shenyu et al. 2022	466	18.34	2.0124	228	16.65	1.5399	1.10	[1.08; 1.12]	1.3%	4.2%
Chen et al. 2022	322	9.16	1.0247	332	6.99	0.7640	1.31	[1.29; 1.33]	1.1%	4.2%
Athan et al. 2023	673	4.10	0.1811	673	5.40	0.2347	0.76	[0.76; 0.76]	14.5%	4.2%
Moss et al. 2023	18	1.53	0.5002	18	0.59	0.7186	2.60	[1.45; 4.66]	0.0%	1.2%
Murdoch et al. 2023	564	5.90	0.3316	564	6.30	0.3827	0.94	[0.93; 0.94]	6.8%	4.2%
Widagdo et al. 2023	337	2.98	0.2120	338	3.67	0.2578	0.81	[0.80; 0.82]	2.8%	4.2%
Cannon et al. 2023	895	2.00	0.1020	896	2.00	0.1020	1.00	[1.00; 1.00]	14.2%	4.2%
Aydillo et al. 2024	82	6.91	3.9787	46	5.92	3.3085	1.17	[0.95; 1.43]	0.0%	3.2%
Nazareth et al. 2024	234	3.60	0.2806	186	3.00	0.4592	1.20	[1.17; 1.23]	0.5%	4.1%
Zhu et al. 2024	156	8.33	0.9031	296	8.00	0.4439	1.04	[1.02; 1.06]	1.0%	4.2%
Clark et al. 2024	523	5.10	0.2704	522	6.87	0.4056	0.74	[0.74; 0.75]	6.8%	4.2%
Buynak et al 2024	456	6.20	0.3852	440	5.87	0.3623	1.06	[1.05; 1.06]	4.8%	4.2%
Naficy et al. 2024	462	4.23	0.2577	454	3.78	0.2168	1.12	[1.11; 1.13]	5.4%	4.2%
Huang et al. 2024	136	4.37	0.5160	121	4.93	0.5941	0.89	[0.86; 0.91]	0.4%	4.1%
**Common effect model**	**9955**			**9656**			**0.96**	**[0.96; 0.97]**	**100.0%**	**.**
**Random effects model**							**1.05**	**[0.97; 1.13]**	**.**	**100.0%**

Heterogeneity: $I^2 = 99.9\%$, $\tau^2 = 0.0369$, $p = 0$

Test for overall effect (random effects): $z = 1.21$ ($p = 0.2264$)

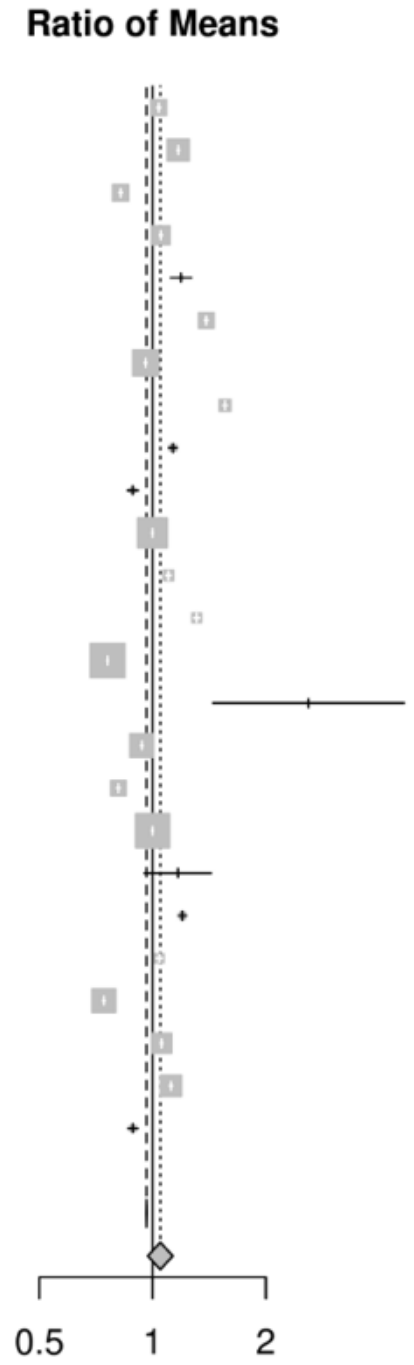

Figure S2: Influenza H3N2 Ratio of Means (ROM) comparing geometric mean fold rise (GMFR) between co-administration (CO) and non-concomitant groups (SEQ). Squares represent study-specific estimates, with square size proportional to study weight, and horizontal lines represent 95% confidence intervals. Diamonds represent overall estimates. ROM values greater than 1 indicate higher GMFR in the co-administration group.

Figure S3: GMFR Influenza B

Study	CO Total	CO Mean	CO SD	SEQ Total	SEQ Mean	SEQ SD	ROM	95%-CI	Weight (common)	Weight (random)
Weston et al. 2009	711	7.10	0.4250	676	6.05	0.3630	1.17	[1.17; 1.18]	6.5%	6.7%
Schwarz et al. 2011	548	4.05	0.2798	546	3.93	0.2740	1.03	[1.02; 1.04]	3.8%	6.7%
Frenck et al. 2012	530	4.80	0.2551	531	5.20	0.3061	0.92	[0.92; 0.93]	5.7%	6.7%
Song et al. 2015	107	2.07	0.3233	56	2.02	0.3203	1.02	[0.97; 1.08]	0.1%	6.1%
Song et al. 2016	373	2.26	0.1279	382	2.05	0.1195	1.10	[1.10; 1.11]	3.9%	6.7%
Thompson et al. 2019	427	2.15	0.0906	430	2.15	0.0867	1.00	[0.99; 1.01]	8.5%	6.7%
Toback et al. 2021	217	3.74	0.3698	214	3.73	0.4425	1.00	[0.98; 1.02]	0.6%	6.6%
Severance et al. 2022	569	2.20	0.0893	561	2.25	0.0893	0.98	[0.97; 0.98]	11.9%	6.7%
Athan et al. 2023	673	2.60	0.1020	673	2.90	0.1199	0.90	[0.89; 0.90]	14.0%	6.7%
Murdoch et al. 2023	564	4.40	0.2679	564	4.85	0.2934	0.91	[0.90; 0.91]	5.2%	6.7%
Cannon et al. 2023	895	1.55	0.0510	896	1.55	0.0765	1.00	[1.00; 1.00]	17.2%	6.7%
Nazareth et al. 2024	234	2.05	0.1148	186	1.70	0.1658	1.21	[1.19; 1.23]	1.0%	6.7%
Zhu et al. 2024	156	6.13	0.5689	296	6.30	0.4796	0.97	[0.96; 0.99]	0.9%	6.7%
Clark et al. 2024	523	1.92	0.0651	522	1.98	0.0727	0.97	[0.97; 0.97]	14.1%	6.7%
Naficy et al. 2024	462	2.54	0.1237	454	2.52	0.1225	1.01	[1.00; 1.01]	6.5%	6.7%
**Common effect model**	**6989**			**6987**			**0.99**	**[0.98; 0.99]**	**100.0%**	**.**
**Random effects model**							**1.01**	**[0.97; 1.05]**	**.**	**100.0%**

Heterogeneity: $I^2 = 99.8\%$, $\tau^2 = 0.0072$, $p = 0$

Test for overall effect (random effects): $z = 0.41$ ($p = 0.6788$)

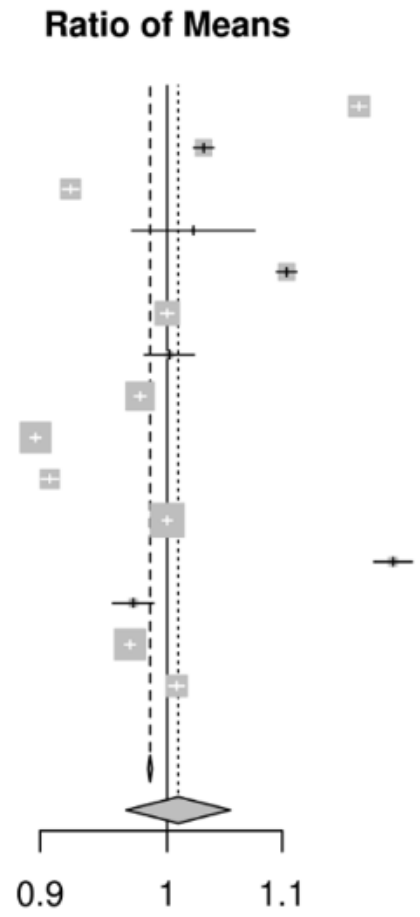

Figure S3: Influenza B Ratio of Means (ROM) comparing geometric mean fold rise (GMFR) between co-administration (CO) and non-concomitant groups (SEQ). Squares represent study-specific estimates, with square size proportional to study weight, and horizontal lines represent 95% confidence intervals. Diamonds represent overall estimates. ROM values greater than 1 indicate higher GMFR in the co-administration group.

Secondary outcome: seroprotection

Figure S4: Seroprotection rate Influenza H1N1

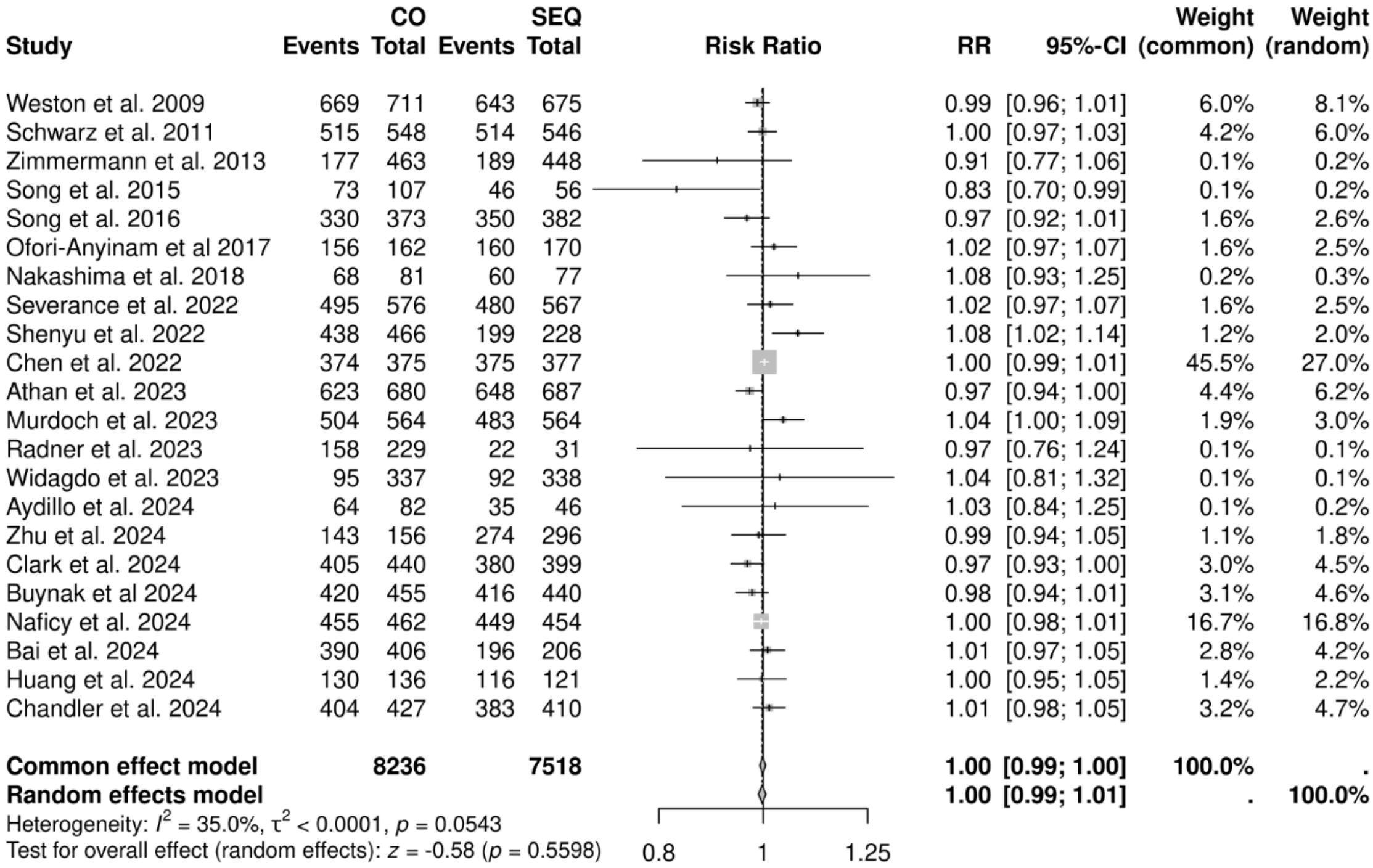

Figure S4: Influenza H1N1 risk ratio (RR) comparing seroprotection rates (SPR) between co-administration (CO) and non-concomitant groups (SEQ). Squares represent study-specific estimates, with square size proportional to study weight, and horizontal lines represent 95% confidence intervals. Diamonds represent overall estimates. RR values greater than 1 indicate higher seroprotection in the co-administration group.

Figure S5: Seroprotection rate Influenza H3N2

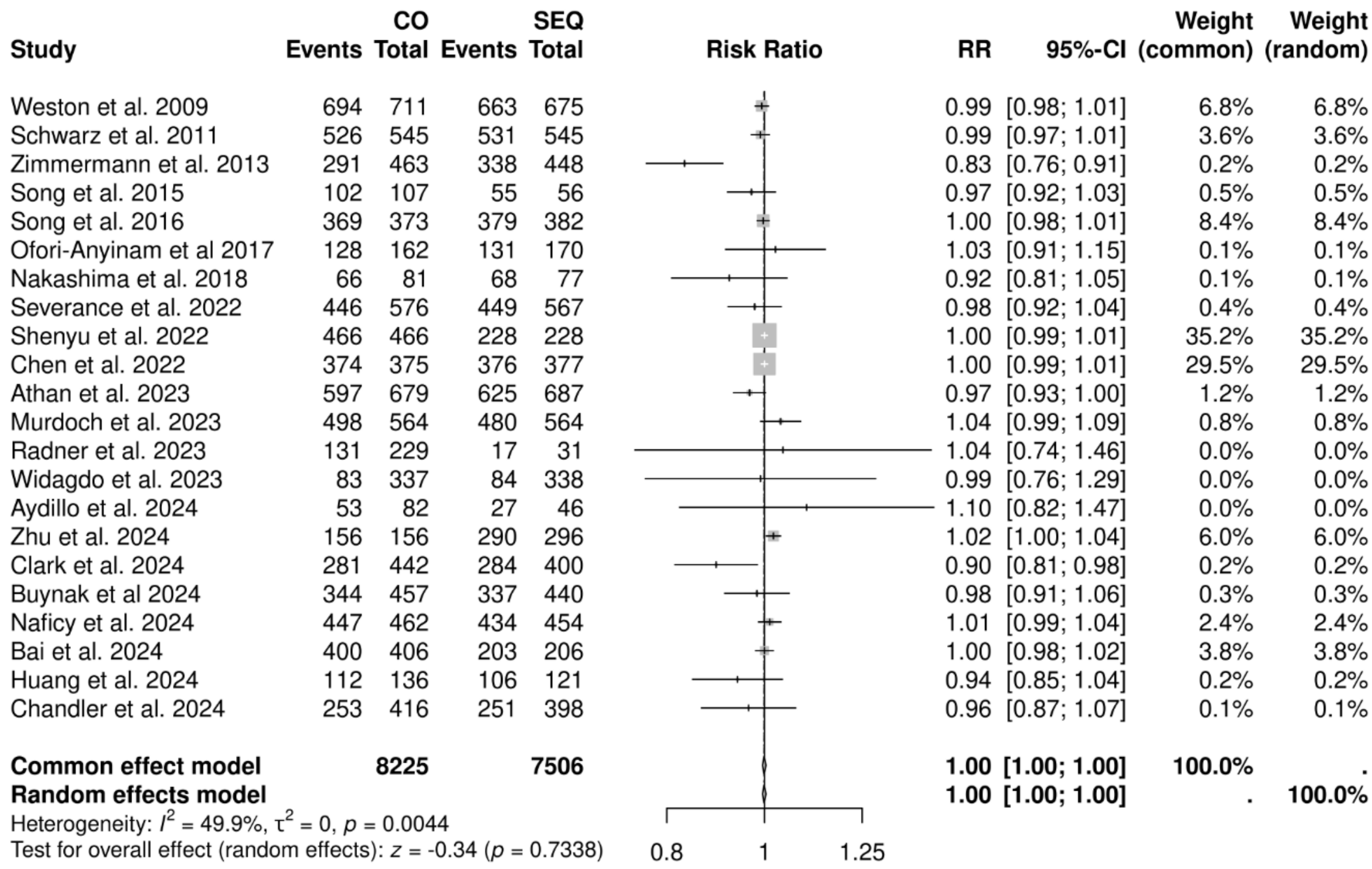

Figure S5: Influenza H3N2 risk ratio (RR) comparing seroprotection rates (SPR) between co-administration (CO) and non-concomitant groups (SEQ). Squares represent study-specific estimates, with square size proportional to study weight, and horizontal lines represent 95% confidence intervals. Diamonds represent overall estimates. RR values greater than 1 indicate higher seroprotection in the co-administration group.

Figure S6: Seroprotection rate Influenza B

Study	CO Events	CO Total	SEQ Events	SEQ Total	RR	95%-CI	Weight (common)	Weight (random)
Ofori-Anyinam et al 2017	162	162	169	170	1.01	[0.99; 1.02]	6.9%	6.9%
Nakashima et al. 2018	48	81	48	77	0.95	[0.74; 1.22]	0.0%	0.0%
Severance et al. 2022	317	576	311	567	1.00	[0.90; 1.11]	0.1%	0.1%
Shenyu et al. 2022	440	466	221	228	0.97	[0.94; 1.01]	0.9%	0.9%
Chen et al. 2022	341	375	350	377	0.98	[0.94; 1.02]	0.5%	0.5%
Murdoch et al. 2023	441	564	418	564	1.06	[0.99; 1.13]	0.2%	0.2%
Radner et al. 2023	69	229	8	31	1.17	[0.62; 2.19]	0.0%	0.0%
Widagdo et al. 2023	73	337	72	338	1.02	[0.76; 1.36]	0.0%	0.0%
Aydillo et al. 2024	54	82	27	46	1.12	[0.84; 1.50]	0.0%	0.0%
Zhu et al. 2024	147	156	284	296	0.98	[0.94; 1.03]	0.4%	0.4%
Clark et al. 2024	442	442	400	400	1.00	[1.00; 1.00]	41.9%	41.9%
Buynak et al 2024	457	457	440	440	1.00	[1.00; 1.00]	47.8%	47.8%
Naficy et al. 2024	292	462	294	454	0.98	[0.89; 1.08]	0.1%	0.1%
Bai et al. 2024	395	406	199	206	1.01	[0.98; 1.04]	1.0%	1.0%
Huang et al. 2024	117	136	92	121	1.13	[1.00; 1.28]	0.1%	0.1%
Chandler et al. 2024	204	427	222	410	0.88	[0.77; 1.01]	0.1%	0.1%
**Common effect model**		**5358**		**4725**	**1.00**	**[1.00; 1.00]**	**100.0%**	**.**
**Random effects model**					**1.00**	**[1.00; 1.00]**	**.**	**100.0%**

Heterogeneity: $I^2 = 9.4\%$, $\tau^2 = 0$, $p = 0.3466$

Test for overall effect (random effects): $z = 0.11$ ($p = 0.9109$)

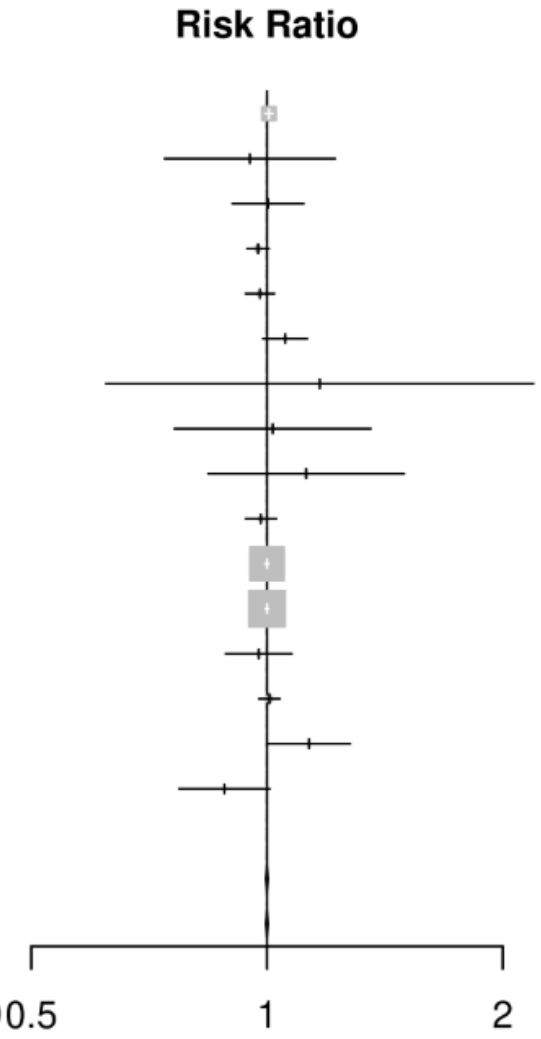

Figure S6: Influenza B risk ratio (RR) comparing seroprotection rates (SPR) between co-administration (CO) and non-concomitant groups (SEQ). Squares represent study-specific estimates, with square size proportional to study weight, and horizontal lines represent 95% confidence intervals. Diamonds represent overall estimates. RR values greater than 1 indicate higher seroprotection in the co-administration group.

Secondary outcome: safety
Figure S7.1: adverse events - any

Study	CO Events	CO Total	SEQ Events	SEQ Total	RR	95%-CI	Weight (common)	Weight (random)
16	125	1684	710	12074	1.26	[1.05; 1.52]	0.7%	5.9%
25	701	806	311	406	1.14	[1.07; 1.21]	6.7%	6.8%
28	474	610	243	332	1.06	[0.98; 1.15]	4.0%	6.7%
30	20	23	23	45	1.70	[1.23; 2.36]	0.2%	4.6%
34	103	412	125	388	0.78	[0.62; 0.97]	0.5%	5.6%
36	525	530	261	273	1.04	[1.01; 1.06]	33.7%	6.9%
39	77	90	51	90	1.51	[1.24; 1.84]	0.6%	5.8%
40	229	488	219	470	1.01	[0.88; 1.15]	1.3%	6.4%
42	482	600	488	596	0.98	[0.93; 1.04]	8.1%	6.8%
5	672	818	1333	1640	1.01	[0.97; 1.05]	15.5%	6.9%
52	202	249	857	982	0.93	[0.87; 0.99]	5.8%	6.8%
57	308	480	293	459	1.01	[0.91; 1.11]	2.6%	6.6%
59	1	160	5	320	0.40	[0.05; 3.40]	0.0%	0.3%
61	251	516	264	513	0.95	[0.84; 1.07]	1.6%	6.5%
64	49	148	97	149	0.51	[0.39; 0.66]	0.4%	5.2%
65	403	438	940	1099	1.08	[1.04; 1.12]	17.8%	6.9%
9	93	384	117	768	1.59	[1.25; 2.03]	0.4%	5.4%
**Common effect model**		**8436**		**20604**	**1.04**	**[1.02; 1.05]**	**100.0%**	**.**
**Random effects model**					**1.05**	**[0.93; 1.18]**	**.**	**100.0%**

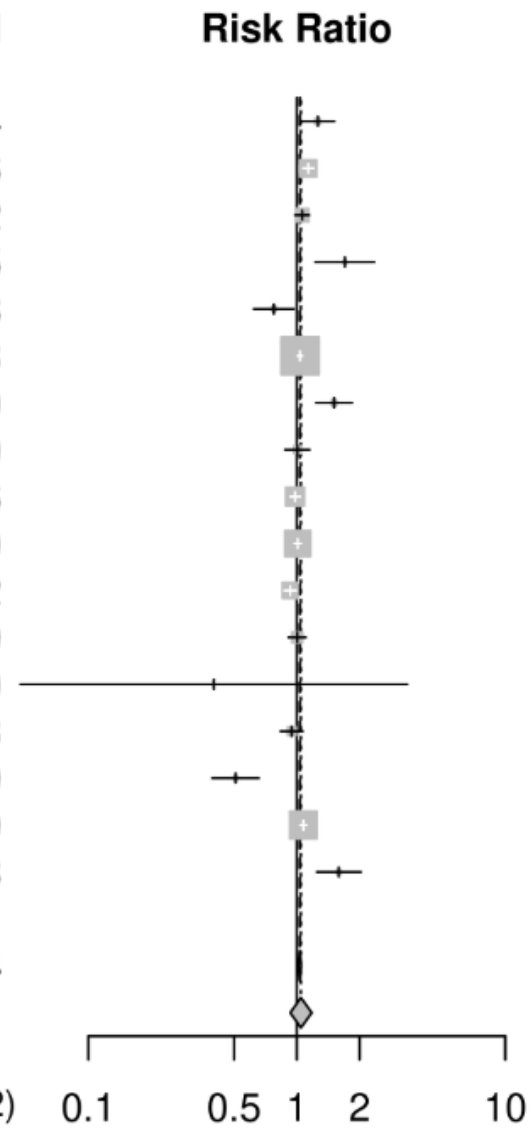

Heterogeneity: $I^2 = 85.2\%$, $\tau^2 = 0.0534$, $p < 0.0001$
Test for overall effect (random effects): $z = 0.76$ ($p = 0.4462$)

Figure S7.1: Risk ratio (RR) for any adverse event comparing co-administration (CO) and non-concomitant groups (SEQ). Squares represent study-specific estimates, with square size proportional to study weight, and horizontal lines represent 95% confidence intervals. Diamonds represent overall estimates. RR values greater than 1 indicate a higher risk in the co-administration group.

Figure S7.2: adverse events - (serious and/or grade ≥3)

Study	CO Events	CO Total	SEQ Events	SEQ Total	RR	95%-CI	Weight (common)	Weight (random)
10	4	77	1	77	4.00	[0.46; 34.98]	0.0%	1.3%
19	11225	92023	113749	889076	0.95	[0.94; 0.97]	89.9%	5.6%
21	116	1000	54	1000	2.15	[1.57; 2.93]	0.3%	5.2%
22	17	100	23	196	1.45	[0.81; 2.58]	0.1%	4.5%
23	179	1676	168	1594	1.01	[0.83; 1.24]	0.7%	5.4%
24	29	340	14	338	2.06	[1.11; 3.83]	0.1%	4.4%
25	7	806	8	406	0.44	[0.16; 1.21]	0.0%	3.2%
27	149	352	146	339	0.98	[0.83; 1.17]	1.0%	5.5%
34	67	412	30	388	2.10	[1.40; 3.16]	0.2%	5.0%
35	6	177	8	179	0.76	[0.27; 2.14]	0.0%	3.1%
39	21	90	1	90	21.00	[2.89; 152.81]	0.0%	1.4%
40	423	5036	429	4999	0.98	[0.86; 1.11]	1.8%	5.5%
45	9	107	2	109	4.58	[1.01; 20.73]	0.0%	2.1%
46	28	373	32	776	1.82	[1.11; 2.98]	0.1%	4.7%
50	32	205	22	1358	9.64	[5.71; 16.25]	0.1%	4.7%
51	6	1293	11	1322	0.56	[0.21; 1.50]	0.0%	3.2%
53	3	714	7	679	0.41	[0.11; 1.57]	0.0%	2.4%
54	20	385	15	386	1.34	[0.69; 2.57]	0.1%	4.3%
55	928	87899	1906	351596	1.95	[1.80; 2.11]	4.9%	5.6%
57	4	480	6	459	0.64	[0.18; 2.24]	0.0%	2.6%
6	10	895	5	896	2.00	[0.69; 5.83]	0.0%	3.0%
60	1	523	6	522	0.17	[0.02; 1.38]	0.0%	1.3%
61	7	516	4	513	1.74	[0.51; 5.91]	0.0%	2.7%
62	81	498	86	497	0.94	[0.71; 1.24]	0.4%	5.3%
65	8	438	13	1099	1.54	[0.64; 3.70]	0.0%	3.6%
8	24	438	20	438	1.20	[0.67; 2.14]	0.1%	4.5%
**Common effect model**		**196853**		**1259332**	**1.00**	**[0.98; 1.01]**	**100.0%**	**.**
**Random effects model**					**1.41**	**[1.07; 1.86]**	**.**	**100.0%**

Heterogeneity: $I^2 = 94.5\%$, $\tau^2 = 0.3541$, $p < 0.0001$
Test for overall effect (random effects): $z = 2.44$ ($p = 0.0147$)

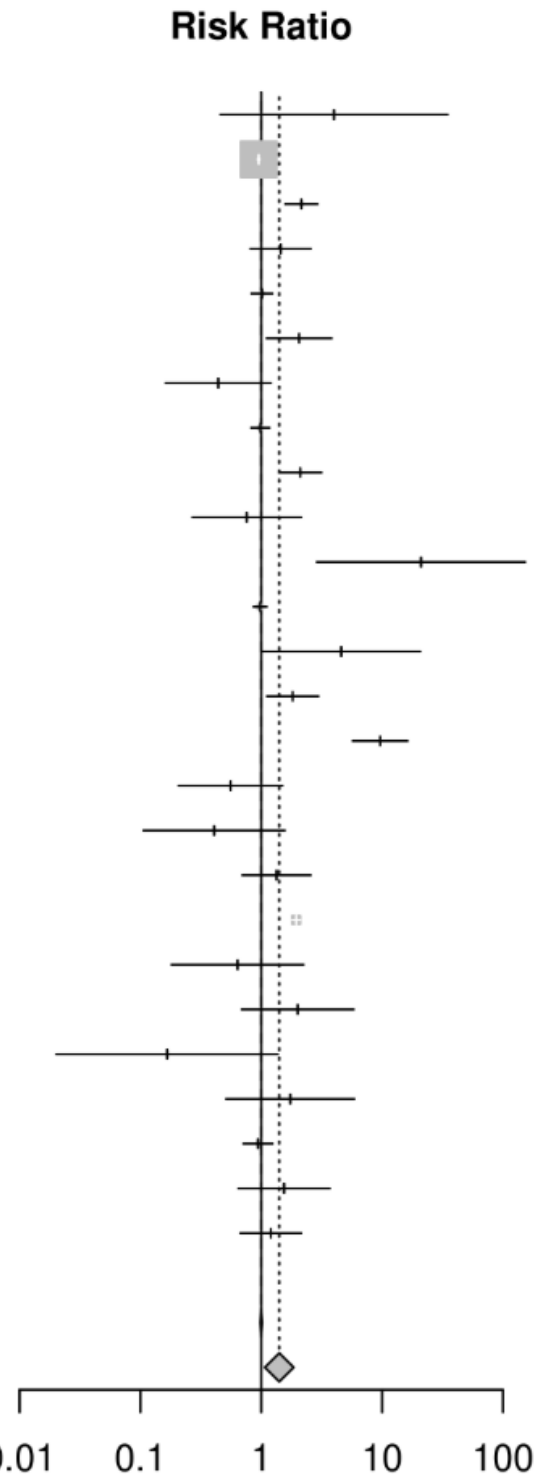

Figure S7.2: Risk ratio (RR) for serious and/or grade ≥3 adverse events comparing co-administration (CO) and non-concomitant groups (SEQ). Squares represent study-specific estimates, with square size proportional to study weight, and horizontal lines represent 95% confidence intervals. Diamonds represent overall estimates. RR values greater than 1 indicate a higher risk in the co-administration group.

Figure S8: adverse events - arthralgia

Study	CO Events	CO Total	SEQ Events	SEQ Total	RR	95%-CI	Weight (common)	Weight (random)
1	84	701	62	693	1.34	[0.98; 1.83]	0.2%	6.4%
10	9	77	3	77	3.00	[0.84; 10.66]	0.0%	1.6%
12	7	38	10	114	2.10	[0.86; 5.13]	0.0%	2.6%
15	182	551	138	560	1.34	[1.11; 1.62]	0.5%	7.4%
19	18218	92023	182941	889076	0.96	[0.95; 0.98]	97.2%	8.0%
24	71	340	50	338	1.41	[1.02; 1.96]	0.2%	6.3%
27	44	352	32	339	1.32	[0.86; 2.04]	0.1%	5.5%
31	84	564	83	553	0.99	[0.75; 1.31]	0.2%	6.7%
32	11	161	11	0			0.0%	0.0%
35	23	177	25	179	0.93	[0.55; 1.58]	0.1%	4.7%
37	10	117	13	127	0.83	[0.38; 1.83]	0.0%	3.1%
39	32	90	5	90	6.40	[2.61; 15.68]	0.0%	2.6%
40	73	1190	59	0			0.0%	0.0%
42	56	600	69	596	0.81	[0.58; 1.13]	0.2%	6.3%
45	11	107	9	109	1.25	[0.54; 2.88]	0.0%	2.9%
46	27	373	44	776	1.28	[0.80; 2.03]	0.1%	5.2%
50	15	205	77	1358	1.29	[0.76; 2.20]	0.1%	4.7%
60	135	523	81	522	1.66	[1.30; 2.13]	0.3%	7.0%
61	83	516	71	513	1.16	[0.87; 1.56]	0.2%	6.6%
62	148	498	153	497	0.97	[0.80; 1.17]	0.5%	7.4%
8	71	438	21	438	3.38	[2.12; 5.40]	0.1%	5.1%
**Common effect model**		**99641**		**896955**	**0.97**	**[0.96; 0.98]**	**100.0%**	.
**Random effects model**					**1.31**	**[1.10; 1.57]**	.	**100.0%**

Heterogeneity: $I^2 = 81.6\%$, $\tau^2 = 0.1015$, $p < 0.0001$
Test for overall effect (random effects): $z = 3.02$ ($p = 0.0025$)

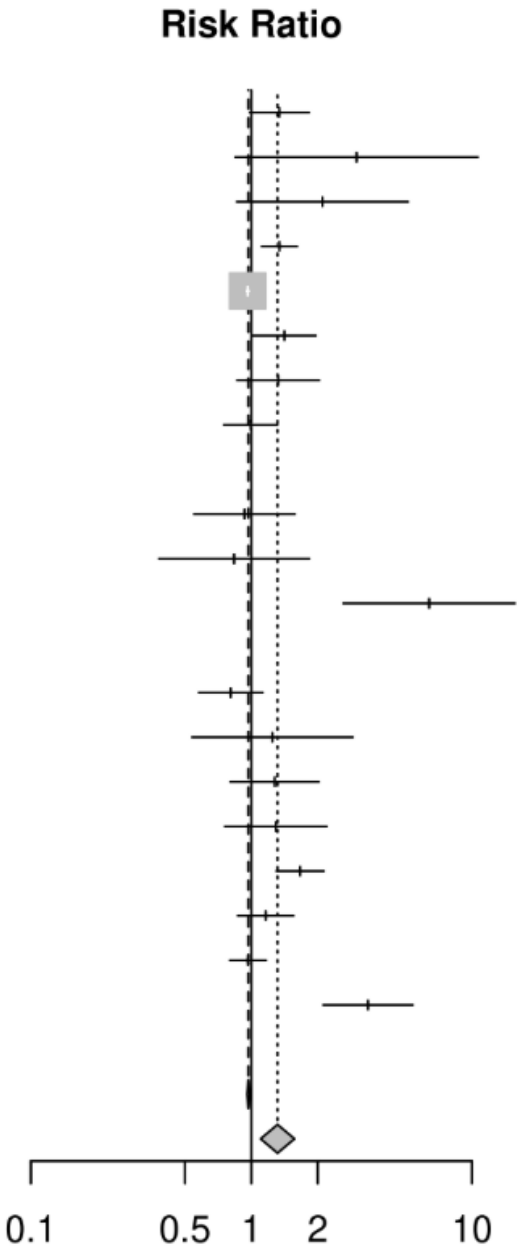

Figure S8: Risk ratio (RR) for arthralgia comparing co-administration (CO) and non-concomitant groups (SEQ). Squares represent study-specific estimates, with square size proportional to study weight, and horizontal lines represent 95% confidence intervals. Diamonds represent overall estimates. RR values greater than 1 indicate a higher risk in the co-administration group.

Figure S9: adverse events - chills

Study	CO Events	CO Total	SEQ Events	SEQ Total	RR	95%-CI	Weight (common)	Weight (random)
10	21	77	10	77	2.10	[1.06; 4.16]	0.0%	6.1%
12	5	38	11	114	1.36	[0.51; 3.67]	0.0%	4.5%
15	173	551	118	560	1.49	[1.22; 1.82]	0.5%	8.7%
19	17977	92023	186490	889076	0.93	[0.92; 0.94]	98.0%	9.0%
2	19	82	3	46	3.55	[1.11; 11.37]	0.0%	3.8%
22	33	100	21	196	3.08	[1.88; 5.03]	0.1%	7.3%
24	56	340	57	338	0.98	[0.70; 1.37]	0.2%	8.1%
27	51	352	46	339	1.07	[0.74; 1.55]	0.1%	7.9%
31	113	564	106	553	1.05	[0.82; 1.33]	0.3%	8.5%
34	5	412	7	388	0.67	[0.22; 2.10]	0.0%	3.9%
37	8	117	13	127	0.67	[0.29; 1.55]	0.0%	5.3%
39	33	90	4	90	8.25	[3.05; 22.33]	0.0%	4.5%
40	61	1190	40	0			0.0%	0.0%
45	24	107	7	109	3.49	[1.57; 7.76]	0.0%	5.5%
46	44	373	69	776	1.33	[0.93; 1.90]	0.1%	8.0%
62	145	498	165	497	0.88	[0.73; 1.06]	0.5%	8.7%
**Common effect model**		**96914**		**893286**	**0.94**	**[0.92; 0.95]**	**100.0%**	**.**
**Random effects model**					**1.44**	**[1.07; 1.94]**	**.**	**100.0%**

Heterogeneity: $I^2 = 84.4\%$, $\tau^2 = 0.2586$, $p < 0.0001$
Test for overall effect (random effects): $z = 2.38$ ($p = 0.0173$)

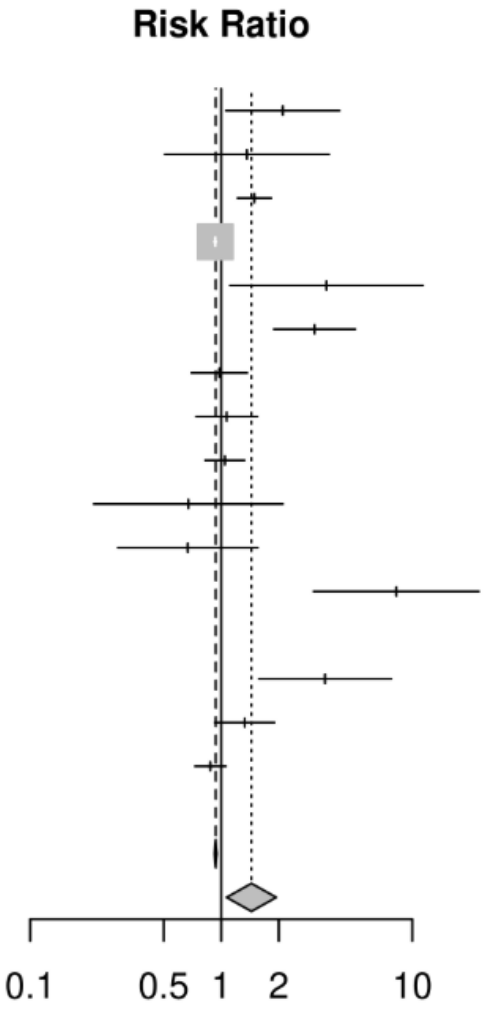

Figure S9: Risk ratio (RR) for chills comparing co-administration (CO) and non-concomitant groups (SEQ). Squares represent study-specific estimates, with square size proportional to study weight, and horizontal lines represent 95% confidence intervals. Diamonds represent overall estimates. RR values greater than 1 indicate a higher risk in the co-administration group.

Figure S10: adverse events – fatigue

Study	CO Events	CO Total	SEQ Events	SEQ Total	RR	95%-CI	Weight (common)	Weight (random)
1	210	701	187	693	1.11	[0.94; 1.31]	0.2%	4.5%
10	36	77	24	77	1.50	[1.00; 2.26]	0.0%	3.5%
12	9	38	21	114	1.29	[0.65; 2.56]	0.0%	2.3%
15	320	551	293	560	1.11	[1.00; 1.23]	0.4%	4.7%
17	35	146	49	442	2.16	[1.46; 3.20]	0.0%	3.6%
19	43323	92023	410196	889076	1.02	[1.01; 1.03]	95.6%	4.8%
22	62	100	72	196	1.69	[1.33; 2.14]	0.1%	4.2%
24	198	340	175	338	1.12	[0.98; 1.29]	0.3%	4.6%
31	361	564	519	553	0.68	[0.64; 0.73]	1.2%	4.7%
32	9	161	19	0			0.0%	0.0%
35	43	177	43	179	1.01	[0.70; 1.46]	0.0%	3.7%
37	40	117	33	127	1.32	[0.89; 1.94]	0.0%	3.6%
39	44	90	15	90	2.93	[1.77; 4.87]	0.0%	3.1%
40	178	1190	154	0			0.0%	0.0%
42	163	600	179	596	0.90	[0.76; 1.08]	0.2%	4.5%
43	1	720	1	240	0.33	[0.02; 5.31]	0.0%	0.3%
45	22	107	11	109	2.04	[1.04; 3.99]	0.0%	2.4%
46	102	373	173	776	1.23	[0.99; 1.52]	0.1%	4.4%
50	57	205	283	1358	1.33	[1.05; 1.70]	0.1%	4.2%
52	119	249	517	982	0.91	[0.79; 1.05]	0.2%	4.6%
54	242	385	129	386	1.88	[1.60; 2.21]	0.2%	4.5%
6	297	895	204	896	1.46	[1.25; 1.70]	0.2%	4.5%
60	239	523	149	522	1.60	[1.36; 1.89]	0.2%	4.5%
61	157	516	113	513	1.38	[1.12; 1.70]	0.1%	4.4%
62	287	498	316	497	0.91	[0.82; 1.00]	0.5%	4.7%
64	4	148	10	149	0.40	[0.13; 1.26]	0.0%	1.2%
65	262	438	228	1099	2.88	[2.51; 3.31]	0.3%	4.6%
8	98	438	56	438	1.75	[1.30; 2.36]	0.1%	4.0%
**Common effect model**		**102370**		**901006**	**1.02**	**[1.02; 1.03]**	**100.0%**	**.**
**Random effects model**					**1.31**	**[1.13; 1.52]**	**.**	**100.0%**

Heterogeneity: $I^2 = 95.6\%$, $\tau^2 = 0.1188$, $p < 0.0001$
Test for overall effect (random effects): $z = 3.62$ ($p = 0.0003$)

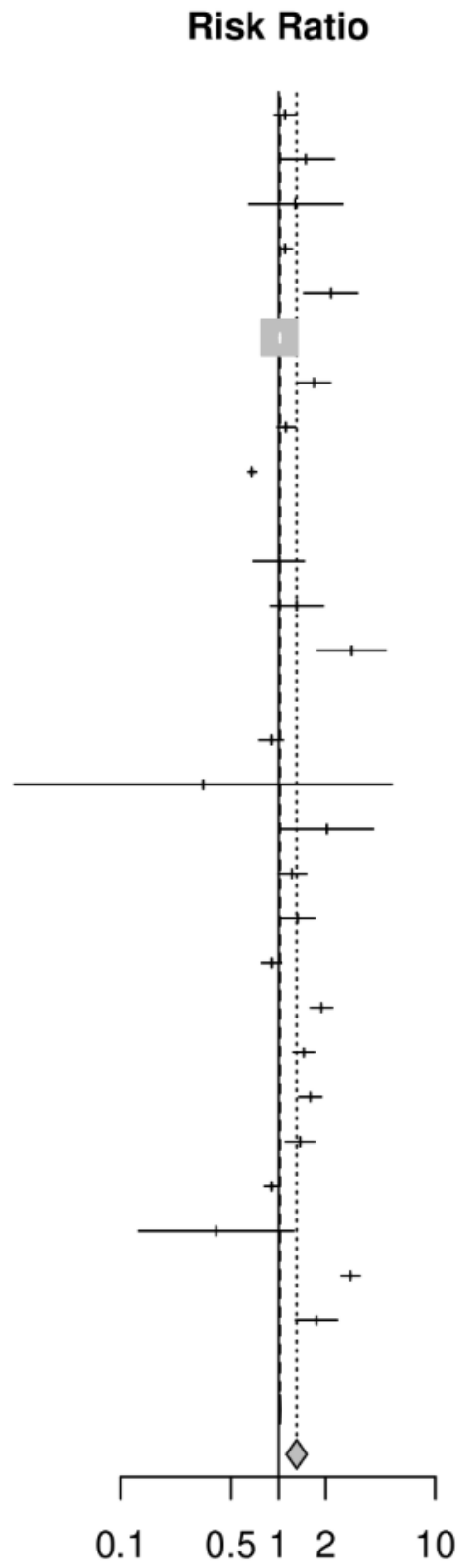

Figure S10: Risk ratio (RR) for fatigue comparing co-administration (CO) and non-concomitant groups (SEQ). Squares represent study-specific estimates, with square size proportional to study weight, and horizontal lines represent 95% confidence intervals. Diamonds represent overall estimates. RR values greater than 1 indicate a higher risk in the co-administration group.

Figure S11: adverse events – fever

Study	CO Events	CO Total	SEQ Events	SEQ Total	Risk Ratio	RR	95%-CI	Weight (common)	Weight (random)
1	14	701	7	693		1.98	[0.80; 4.87]	0.0%	2.8%
10	8	77	5	77		1.60	[0.55; 4.67]	0.0%	2.2%
11	3	61	2	63		1.55	[0.27; 8.95]	0.0%	1.0%
12	1	38	5	114		0.60	[0.07; 4.98]	0.0%	0.7%
16	12	1684	66	12074		1.30	[0.71; 2.41]	0.0%	4.2%
17	9	146	10	442		2.72	[1.13; 6.57]	0.0%	2.9%
19	19618	92023	194919	889076		0.97	[0.96; 0.99]	97.2%	7.2%
2	20	82	2	46		5.61	[1.37; 22.93]	0.0%	1.5%
21	24	1000	10	1000		2.40	[1.15; 4.99]	0.0%	3.5%
22	12	100	11	196		2.14	[0.98; 4.67]	0.0%	3.3%
23	46	579	35	0				0.0%	0.0%
24	3	340	4	338		0.75	[0.17; 3.31]	0.0%	1.3%
25	387	806	160	406		1.22	[1.06; 1.40]	0.8%	7.0%
27	15	352	8	339		1.81	[0.78; 4.20]	0.0%	3.0%
28	104	610	60	332		0.94	[0.71; 1.26]	0.2%	6.2%
31	11	564	17	553		0.63	[0.30; 1.34]	0.0%	3.4%
32	2	161	3	0				0.0%	0.0%
34	213	412	113	388		1.78	[1.48; 2.13]	0.5%	6.8%
35	2	177	1	179		2.02	[0.19; 22.11]	0.0%	0.6%
37	11	117	18	127		0.66	[0.33; 1.34]	0.0%	3.6%
40	18	1190	14	0				0.0%	0.0%
43	2	720	3	240		0.22	[0.04; 1.32]	0.0%	1.0%
44	40	541	20	320		1.18	[0.70; 1.99]	0.1%	4.7%
45	2	107	1	109		2.04	[0.19; 22.14]	0.0%	0.6%
46	3	373	2	776		3.12	[0.52; 18.60]	0.0%	1.0%
5	181	818	360	1640		1.01	[0.86; 1.18]	0.7%	6.9%
50	9	205	26	1358		2.29	[1.09; 4.82]	0.0%	3.5%
54	24	385	2	386		12.03	[2.86; 50.56]	0.0%	1.4%
57	7	480	4	459		1.67	[0.49; 5.68]	0.0%	1.8%
60	11	523	3	522		3.66	[1.03; 13.04]	0.0%	1.7%
61	11	516	4	513		2.73	[0.88; 8.53]	0.0%	2.0%
62	29	498	30	497		0.96	[0.59; 1.58]	0.1%	4.9%
64	5	148	13	149		0.39	[0.14; 1.06]	0.0%	2.4%
65	13	438	30	1099		1.09	[0.57; 2.06]	0.0%	4.0%
8	11	438	3	438		3.67	[1.03; 13.05]	0.0%	1.7%
9	2	1536	4	768		0.25	[0.05; 1.36]	0.0%	1.1%
**Common effect model**		**108946**		**915717**		**0.98**	**[0.97; 0.99]**	**100.0%**	**.**
**Random effects model**						**1.35**	**[1.11; 1.63]**	**.**	**100.0%**

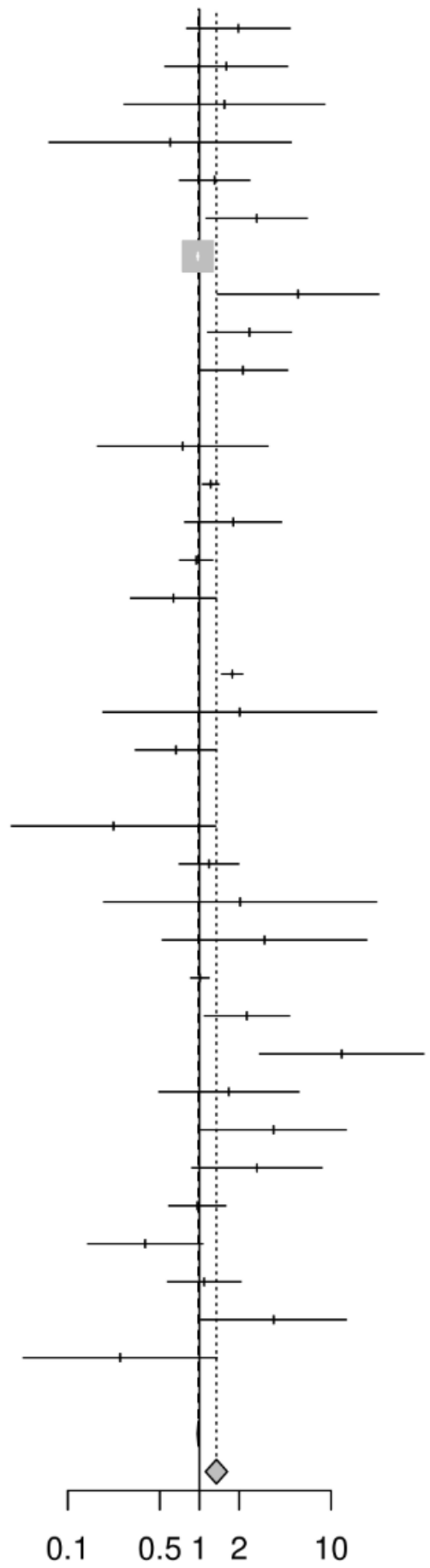

Heterogeneity: $I^2 = 73.8\%$, $\tau^2 = 0.1315$, $p < 0.0001$

Test for overall effect (random effects): $z = 3.04$ ($p = 0.0024$)

Figure S11: Risk ratio (RR) for fever comparing co-administration (CO) and non-concomitant groups (SEQ). Squares represent study-specific estimates, with square size proportional to study weight, and horizontal lines represent 95% confidence intervals. Diamonds represent overall estimates. RR values greater than 1 indicate a higher risk in the co-administration group.

Figure S12: adverse events – headache

Study	CO Events	CO Total	SEQ Events	SEQ Total	RR	95%-CI	Weight (common)	Weight (random)
1	140	701	145	693	0.95	[0.78; 1.17]	0.2%	4.2%
10	19	77	14	77	1.36	[0.73; 2.51]	0.0%	2.7%
11	5	61	3	63	1.72	[0.43; 6.89]	0.0%	1.0%
12	12	38	23	114	1.57	[0.86; 2.83]	0.0%	2.8%
15	363	551	316	560	1.17	[1.06; 1.28]	0.8%	4.5%
16	18	1684	88	12074	1.47	[0.89; 2.43]	0.0%	3.1%
19	33892	92023	332651	889076	0.98	[0.98; 0.99]	95.9%	4.5%
22	51	100	54	196	1.85	[1.37; 2.49]	0.1%	3.9%
23	15	579	8	0			0.0%	0.0%
24	159	340	134	338	1.18	[0.99; 1.40]	0.3%	4.3%
27	140	352	128	339	1.05	[0.87; 1.27]	0.2%	4.3%
31	265	564	402	553	0.65	[0.58; 0.72]	0.7%	4.5%
32	4	161	5	0			0.0%	0.0%
34	12	412	4	388	2.83	[0.92; 8.69]	0.0%	1.4%
35	27	177	30	179	0.91	[0.57; 1.47]	0.0%	3.2%
37	22	117	24	127	1.00	[0.59; 1.68]	0.0%	3.0%
39	35	90	12	90	2.92	[1.62; 5.25]	0.0%	2.8%
40	154	1190	139	0			0.0%	0.0%
42	129	600	141	596	0.91	[0.74; 1.12]	0.2%	4.2%
43	1	720	2	240	0.17	[0.02; 1.83]	0.0%	0.4%
44	82	541	57	320	0.85	[0.63; 1.16]	0.1%	3.9%
45	19	107	9	109	2.15	[1.02; 4.54]	0.0%	2.3%
46	67	373	111	776	1.26	[0.95; 1.66]	0.1%	4.0%
50	51	205	327	1358	1.03	[0.80; 1.33]	0.1%	4.1%
52	93	249	425	982	0.86	[0.72; 1.03]	0.2%	4.3%
54	190	385	76	386	2.51	[2.00; 3.14]	0.1%	4.2%
57	66	480	62	459	1.02	[0.74; 1.40]	0.1%	3.8%
60	168	523	101	522	1.66	[1.34; 2.06]	0.2%	4.2%
61	125	516	88	513	1.41	[1.11; 1.80]	0.1%	4.1%
64	4	148	4	149	1.01	[0.26; 3.95]	0.0%	1.0%
65	225	438	189	1099	2.99	[2.55; 3.50]	0.3%	4.4%
8	95	438	56	438	1.70	[1.25; 2.30]	0.1%	3.9%
9	3	1536	7	768	0.21	[0.06; 0.83]	0.0%	1.1%
**Common effect model**		**106476**		**913582**	**0.99**	**[0.98; 1.00]**	**100.0%**	**.**
**Random effects model**					**1.26**	**[1.07; 1.47]**	**.**	**100.0%**

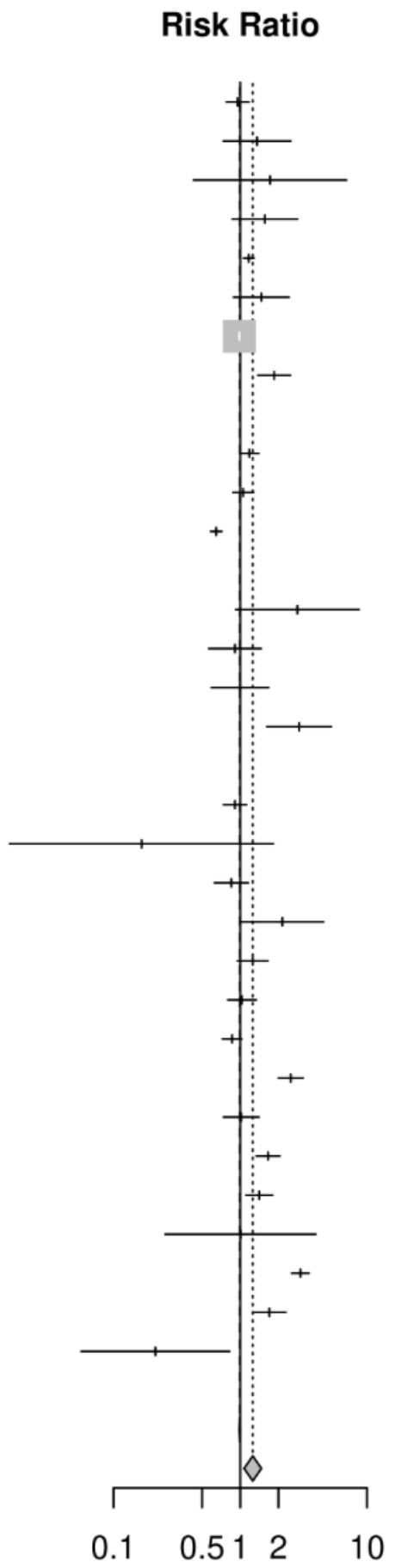

Heterogeneity: $I^2 = 93.4\%$, $\tau^2 = 0.1433$, $p < 0.0001$
Test for overall effect (random effects): $z = 2.82$ ($p = 0.0048$)

Figure S12: Risk ratio (RR) for headache comparing co-administration (CO) and non-concomitant groups (SEQ). Squares represent study-specific estimates, with square size proportional to study weight, and horizontal lines represent 95% confidence intervals. Diamonds represent overall estimates. RR values greater than 1 indicate a higher risk in the co-administration group.

Figure S13: adverse events - myalgia

Study	CO Events	CO Total	SEQ Events	SEQ Total	RR	95%-CI	Weight (common)	Weight (random)
1	112	701	83	693	1.33	[1.02; 1.74]	0.1%	4.6%
10	29	77	27	77	1.07	[0.71; 1.63]	0.0%	3.7%
11	21	61	2	63	10.84	[2.66; 44.29]	0.0%	0.9%
12	13	38	20	114	1.95	[1.08; 3.53]	0.0%	2.8%
19	33729	92023	323279	889076	1.01	[1.00; 1.02]	96.7%	5.5%
23	18	579	4	0			0.0%	0.0%
24	135	340	96	338	1.40	[1.13; 1.73]	0.2%	4.9%
27	103	352	74	339	1.34	[1.03; 1.74]	0.1%	4.6%
31	158	564	195	553	0.79	[0.67; 0.95]	0.3%	5.1%
34	7	412	3	388	2.20	[0.57; 8.44]	0.0%	0.9%
35	28	177	32	179	0.88	[0.56; 1.41]	0.0%	3.5%
37	17	117	22	127	0.84	[0.47; 1.50]	0.0%	2.9%
39	41	90	15	90	2.73	[1.63; 4.57]	0.0%	3.2%
40	126	1190	76	0			0.0%	0.0%
42	142	600	127	596	1.11	[0.90; 1.37]	0.2%	4.9%
43	2	720	1	240	0.67	[0.06; 7.32]	0.0%	0.3%
44	94	541	47	320	1.18	[0.86; 1.63]	0.1%	4.3%
45	24	107	10	109	2.44	[1.23; 4.86]	0.0%	2.4%
46	75	373	117	776	1.33	[1.03; 1.73]	0.1%	4.6%
50	58	205	288	1358	1.33	[1.05; 1.70]	0.1%	4.7%
52	85	249	410	982	0.82	[0.68; 0.99]	0.2%	5.0%
54	221	385	115	386	1.93	[1.62; 2.30]	0.2%	5.1%
57	93	480	78	459	1.14	[0.87; 1.50]	0.1%	4.6%
60	204	523	120	522	1.70	[1.40; 2.05]	0.2%	5.0%
61	207	516	175	513	1.18	[1.00; 1.38]	0.3%	5.1%
62	293	498	320	497	0.91	[0.83; 1.01]	0.8%	5.3%
64	1	148	2	149	0.50	[0.05; 5.49]	0.0%	0.3%
8	97	438	41	438	2.37	[1.68; 3.32]	0.1%	4.2%
9	6	1536	9	768	0.33	[0.12; 0.93]	0.0%	1.4%
**Common effect model**		**104040**		**900150**	**1.01**	**[1.00; 1.02]**	**100.0%**	**.**
**Random effects model**					**1.27**	**[1.10; 1.47]**	**.**	**100.0%**

Heterogeneity: $I^2 = 86.8\%$, $\tau^2 = 0.0971$, $p < 0.0001$
Test for overall effect (random effects): $z = 3.31$ ($p = 0.0009$)

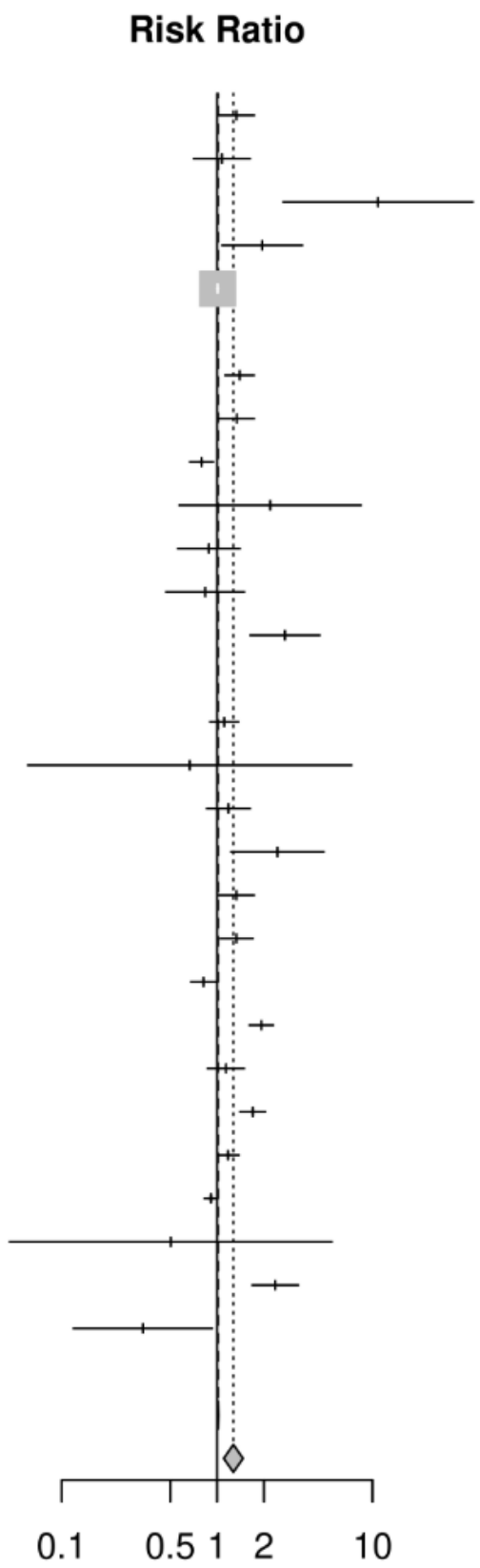

Figure S13: Risk ratio (RR) for myalgia comparing co-administration (CO) and non-concomitant groups (SEQ). Squares represent study-specific estimates, with square size proportional to study weight, and horizontal lines represent 95% confidence intervals. Diamonds represent overall estimates. RR values greater than 1 indicate a higher risk in the co-administration group.

Figure S14: adverse events - cough

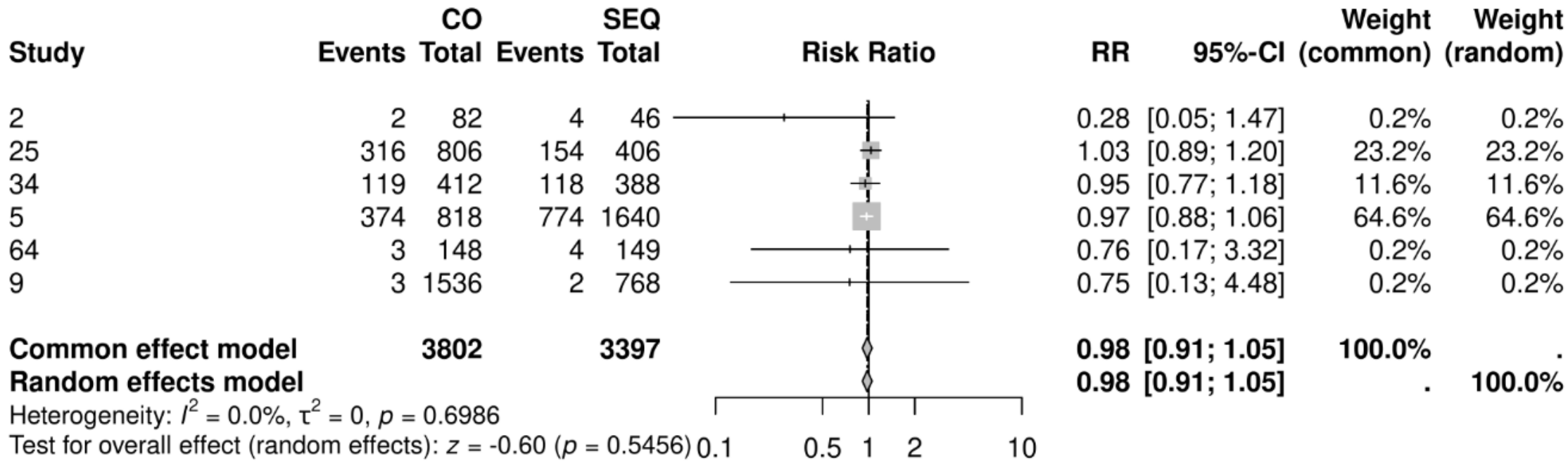

Figure S14: Risk ratio (RR) for cough comparing co-administration (CO) and non-concomitant groups (SEQ). Squares represent study-specific estimates, with square size proportional to study weight, and horizontal lines represent 95% confidence intervals. Diamonds represent overall estimates. RR values greater than 1 indicate a higher risk in the co-administration group.

Figure S15: adverse events - diarrhea

Study	CO Events	CO Total	SEQ Events	SEQ Total	RR	95%-CI	Weight (common)	Weight (random)
1	56	701	42	693	1.32	[0.90; 1.94]	0.4%	11.1%
16	3	1684	18	12074	1.19	[0.35; 4.05]	0.0%	3.1%
19	5738	92023	51420	889076	1.08	[1.05; 1.11]	96.0%	15.7%
24	28	340	24	338	1.16	[0.69; 1.96]	0.2%	8.9%
27	53	352	39	339	1.31	[0.89; 1.92]	0.5%	11.1%
31	68	564	95	553	0.70	[0.53; 0.94]	0.8%	12.8%
34	39	412	33	388	1.11	[0.72; 1.73]	0.3%	10.2%
62	80	498	119	497	0.67	[0.52; 0.87]	1.0%	13.3%
64	4	148	2	149	2.01	[0.37; 10.83]	0.0%	1.8%
65	57	438	75	1099	1.91	[1.38; 2.64]	0.6%	12.1%
**Common effect model**		**97160**		**905206**	**1.08**	**[1.05; 1.10]**	**100.0%**	**.**
**Random effects model**					**1.10**	**[0.87; 1.39]**	**.**	**100.0%**

Heterogeneity: $I^2 = 75.1\%$, $\tau^2 = 0.0937$, $p < 0.0001$

Test for overall effect (random effects): $z = 0.78$ ($p = 0.4370$)

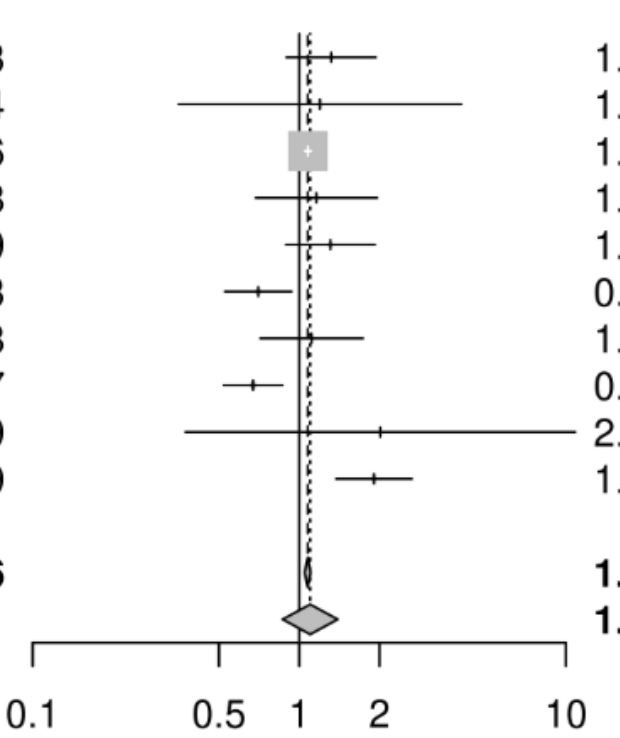

Figure S15: Risk ratio (RR) for diarrhea comparing co-administration (CO) and non-concomitant groups (SEQ). Squares represent study-specific estimates, with square size proportional to study weight, and horizontal lines represent 95% confidence intervals. Diamonds represent overall estimates. RR values greater than 1 indicate a higher risk in the co-administration group.

Figure S16: adverse events - gastrointestinal

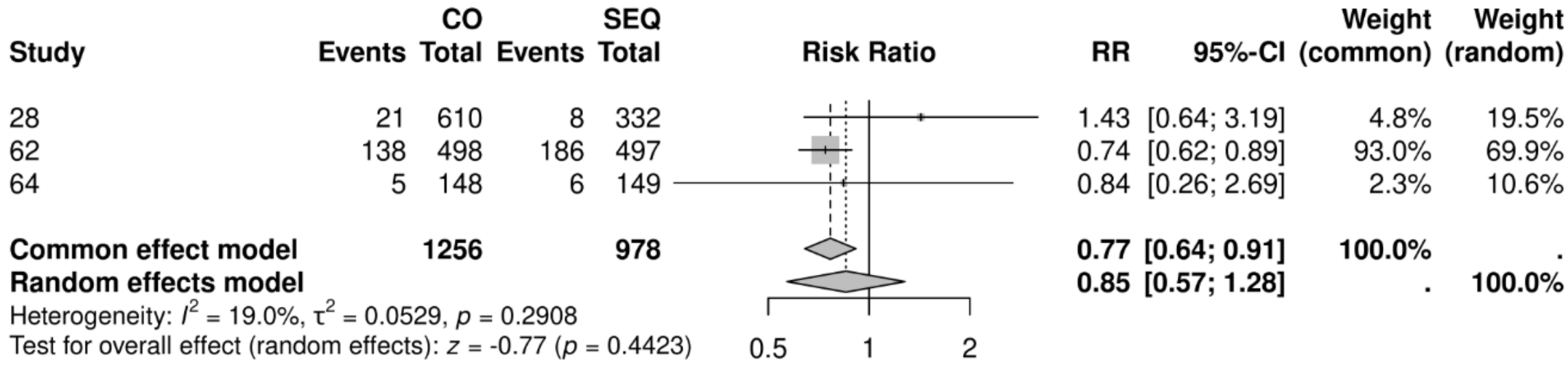

Figure S16: Risk ratio (RR) for gastrointestinal adverse events comparing co-administration (CO) and non-concomitant groups (SEQ). Squares represent study-specific estimates, with square size proportional to study weight, and horizontal lines represent 95% confidence intervals. Diamonds represent overall estimates. RR values greater than 1 indicate a higher risk in the co-administration group.

Figure S17: adverse events - malaise

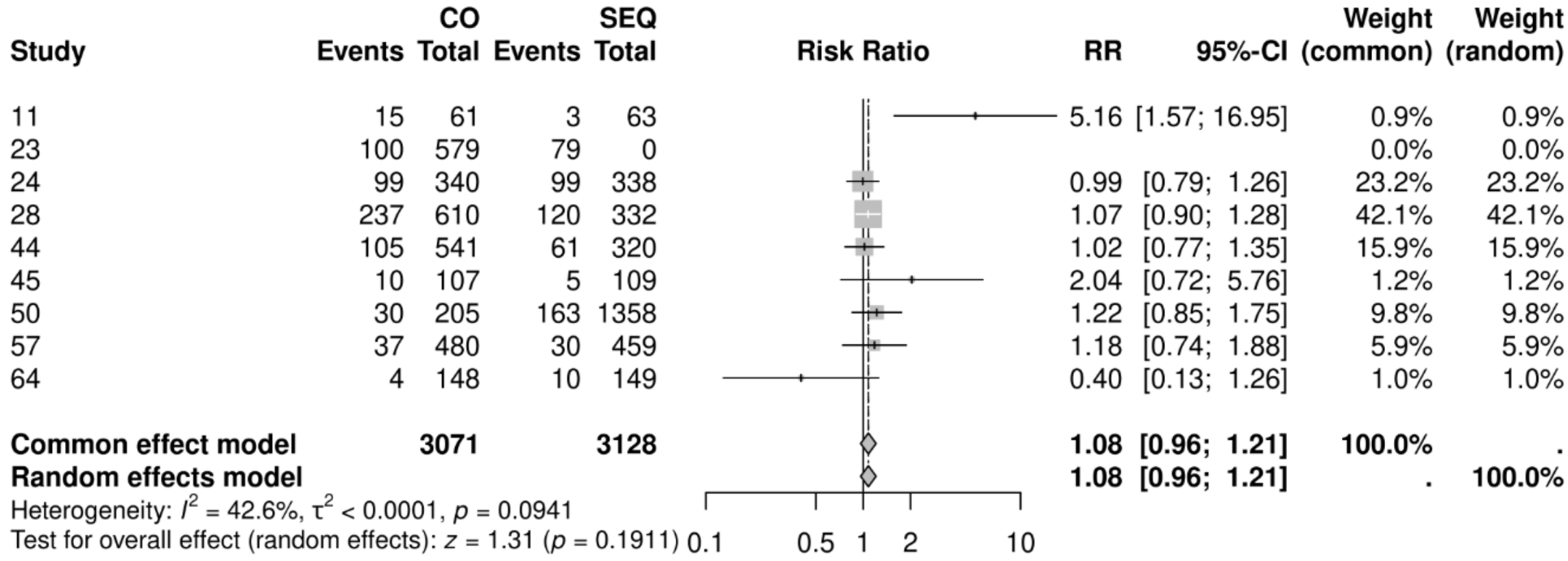

Figure S17: Risk ratio (RR) for malaise comparing co-administration (CO) and non-concomitant groups (SEQ). Squares represent study-specific estimates, with square size proportional to study weight, and horizontal lines represent 95% confidence intervals. Diamonds represent overall estimates. RR values greater than 1 indicate a higher risk in the co-administration group.

Figure S18: adverse events - nausea

Study	CO Events	CO Total	SEQ Events	SEQ Total	RR	95%-CI	Weight (common)	Weight (random)
1	56	701	42	693	1.32	[0.90; 1.94]	0.2%	10.3%
10	4	77	4	77	1.00	[0.26; 3.86]	0.0%	3.9%
11	2	61	2	63	1.03	[0.15; 7.10]	0.0%	2.3%
19	9956	92023	99411	889076	0.97	[0.95; 0.99]	98.0%	12.0%
24	44	340	34	338	1.29	[0.84; 1.96]	0.2%	10.0%
27	47	352	48	339	0.94	[0.65; 1.37]	0.3%	10.4%
37	3	117	5	127	0.65	[0.16; 2.67]	0.0%	3.7%
39	13	90	2	90	6.50	[1.51; 27.98]	0.0%	3.5%
50	11	205	73	1358	1.00	[0.54; 1.85]	0.1%	8.4%
54	76	385	37	386	2.06	[1.43; 2.97]	0.3%	10.4%
62	64	498	106	497	0.60	[0.45; 0.80]	0.5%	11.0%
65	67	438	54	1099	3.11	[2.21; 4.38]	0.3%	10.6%
9	4	1536	3	768	0.67	[0.15; 2.97]	0.0%	3.4%
**Common effect model**		**96823**		**894911**	**0.97**	**[0.95; 0.99]**	**100.0%**	**.**
**Random effects model**					**1.25**	**[0.90; 1.73]**	**.**	**100.0%**

Heterogeneity: $I^2 = 85.6\%$, $\tau^2 = 0.2322$, $p < 0.0001$
Test for overall effect (random effects): $z = 1.32$ ($p = 0.1861$)

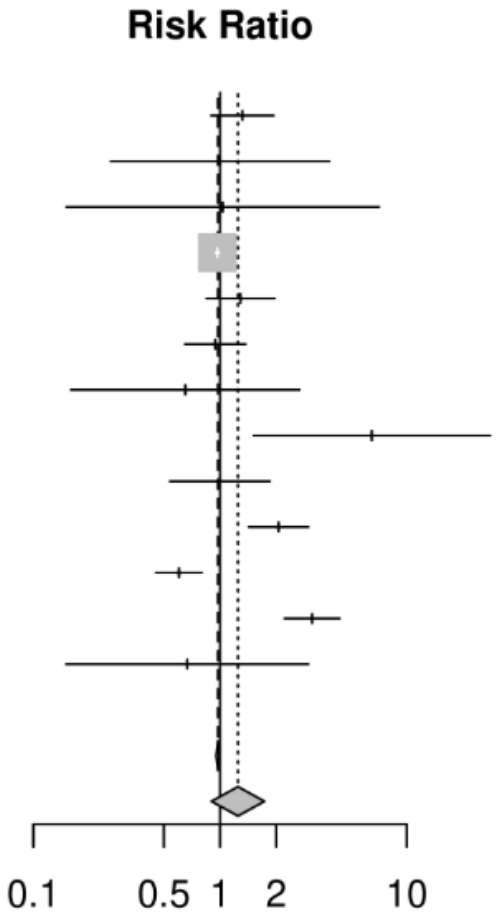

Figure S18: Risk ratio (RR) for nausea comparing co-administration (CO) and non-concomitant groups (SEQ). Squares represent study-specific estimates, with square size proportional to study weight, and horizontal lines represent 95% confidence intervals. Diamonds represent overall estimates. RR values greater than 1 indicate a higher risk in the co-administration group.

Figure S19: adverse events - rash

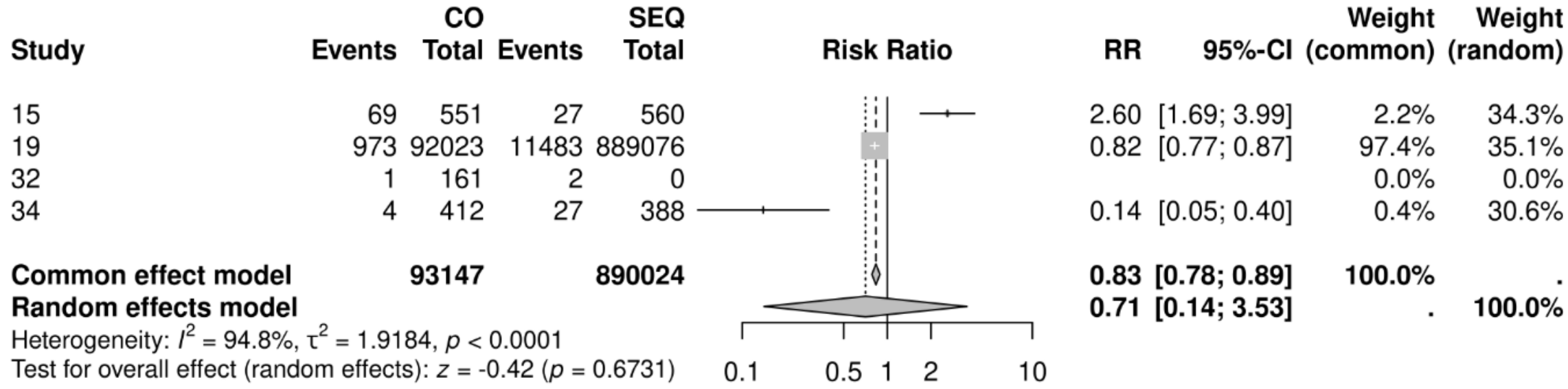

Figure S19: Risk ratio (RR) for rash comparing co-administration (CO) and non-concomitant groups (SEQ). Squares represent study-specific estimates, with square size proportional to study weight, and horizontal lines represent 95% confidence intervals. Diamonds represent overall estimates. RR values greater than 1 indicate a higher risk in the co-administration group.

Figure S20: adverse events - vomiting

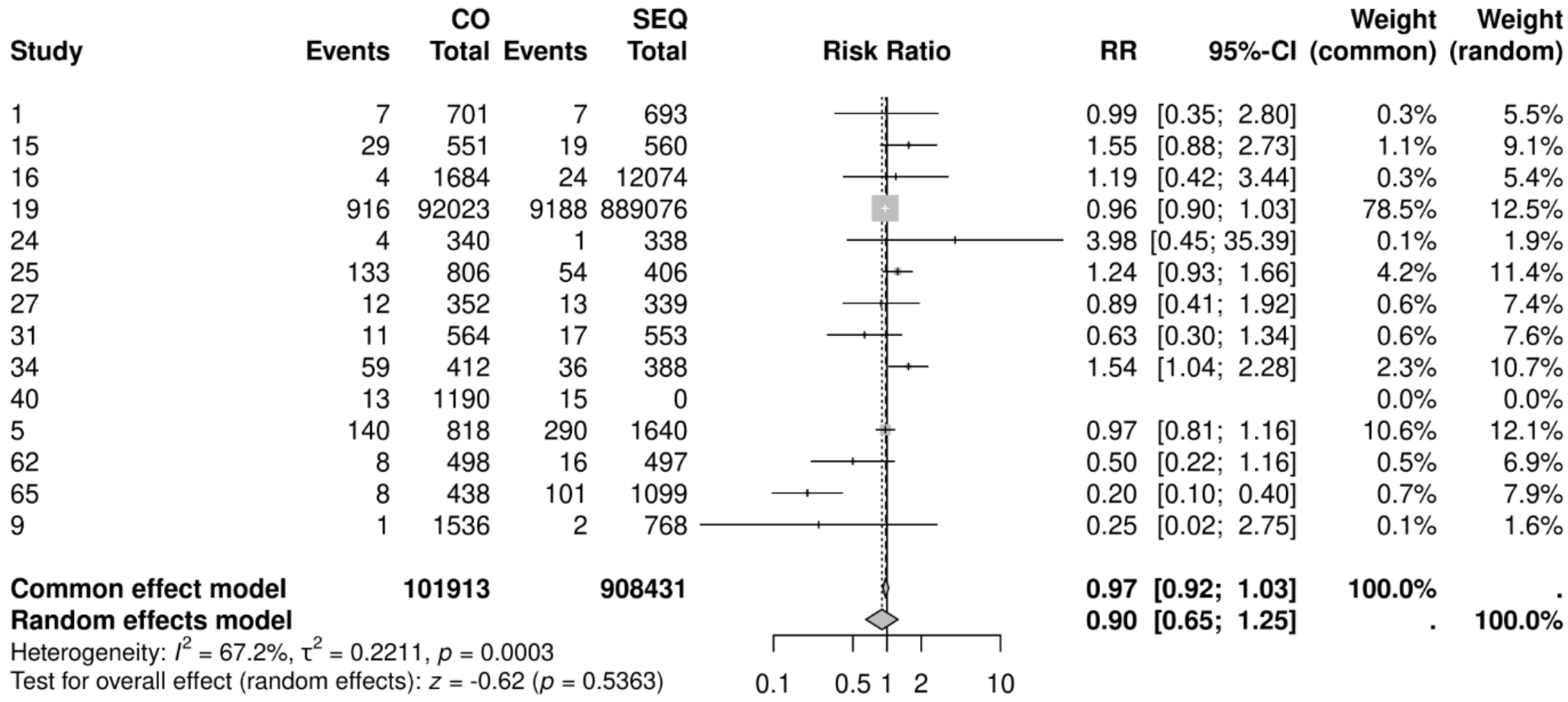

Figure S20: Risk ratio (RR) for vomiting comparing co-administration (CO) and non-concomitant groups (SEQ). Squares represent study-specific estimates, with square size proportional to study weight, and horizontal lines represent 95% confidence intervals. Diamonds represent overall estimates. RR values greater than 1 indicate a higher risk in the co-administration group.

Subgroup analyses

Age subgroups
Figure S21: GMFR Influenza H3N2 - age subgroup analysis

Study	CO Total	CO Mean	CO SD	SEQ Total	SEQ Mean	SEQ SD	ROM	95%-CI	Weight (common)	Weight (random)
**subgroup = aged**										
Schwarz et al. 2011	548	5.21	0.4324	546	6.33	0.5425	0.82	[0.81; 0.83]	3.2%	3.8%
Song et al. 2015	107	2.70	0.5389	56	2.27	0.4528	1.19	[1.12; 1.27]	0.1%	3.7%
Izikson et al. 2022	96	3.10	0.3061	86	3.50	0.4082	0.89	[0.86; 0.91]	0.3%	3.8%
Chen et al. 2022	131	11.02	1.3336	143	7.63	0.8816	1.44	[1.40; 1.49]	0.4%	3.8%
Athan et al. 2023	673	4.10	0.1811	673	5.40	0.2347	0.76	[0.76; 0.76]	14.6%	3.8%
Cannon et al. 2023	895	2.00	0.1020	896	2.00	0.1020	1.00	[1.00; 1.00]	14.3%	3.8%
Clark et al. 2024	523	5.10	0.2704	522	6.87	0.4056	0.74	[0.74; 0.75]	6.9%	3.8%
Buynak et al 2024	456	6.20	0.3852	440	5.87	0.3623	1.06	[1.05; 1.06]	4.9%	3.8%
**Common effect model**	**3429**			**3362**			**0.87**	**[0.87; 0.87]**	**44.7%**	**.**
**Random effects model**							**0.96**	**[0.82; 1.13]**	**.**	**30.6%**
Heterogeneity: I^2 = 99.9%, τ^2 = 0.0534, p = 0										
**subgroup = other**										
Breiman et al. 2009	713	4.01	0.3894	721	3.86	0.3880	1.04	[1.03; 1.05]	3.1%	3.8%
Song et al. 2016	373	5.28	0.3939	382	3.80	0.2862	1.39	[1.37; 1.40]	2.8%	3.8%
Thompson et al. 2019	427	2.30	0.1071	430	2.40	0.1122	0.96	[0.95; 0.96]	8.2%	3.8%
Lazarus et al. 2021	114	1.14	0.1022	38	0.95	0.1070	1.19	[1.15; 1.24]	0.2%	3.8%
Severance et al. 2022	569	2.20	0.1020	561	2.20	0.1020	1.00	[0.99; 1.01]	10.9%	3.8%
Moss et al. 2023	18	1.53	0.5002	18	0.59	0.7186	2.60	[1.45; 4.66]	0.0%	1.2%
Widagdo et al. 2023	337	2.98	0.2120	338	3.67	0.2578	0.81	[0.80; 0.82]	2.8%	3.8%
Zhu et al. 2024	156	8.33	0.9031	296	8.00	0.4439	1.04	[1.02; 1.06]	1.0%	3.8%
**Common effect model**	**2707**			**2784**			**1.01**	**[1.00; 1.01]**	**28.9%**	**.**
**Random effects model**							**1.09**	**[0.94; 1.26]**	**.**	**28.1%**
Heterogeneity: I^2 = 99.9%, τ^2 = 0.0404, p = 0										
**subgroup = young**										
Weston et al. 2009	711	13.61	0.9414	676	11.63	0.8348	1.17	[1.16; 1.18]	5.8%	3.8%
Frenck et al. 2012	530	10.20	0.7143	531	9.70	0.7143	1.05	[1.04; 1.06]	4.3%	3.8%
Lazarus et al. 2021	226	1.64	0.1390	228	0.94	0.0973	1.75	[1.72; 1.78]	1.1%	3.8%
Toback et al. 2021	217	6.98	0.8612	214	6.16	0.8230	1.13	[1.11; 1.16]	0.5%	3.8%
Shenyu et al. 2022	232	18.35	2.3694	228	16.65	1.5399	1.10	[1.08; 1.13]	0.8%	3.8%
Chen et al. 2022	191	7.88	0.8128	189	6.51	0.6750	1.21	[1.19; 1.24]	0.7%	3.8%
Murdoch et al. 2023	564	5.90	0.3316	564	6.30	0.3827	0.94	[0.93; 0.94]	6.9%	3.8%
Aydillo et al. 2024	82	6.91	3.9787	46	5.92	3.3085	1.17	[0.95; 1.43]	0.0%	3.1%
Nazareth et al. 2024	234	3.60	0.2806	186	3.00	0.4592	1.20	[1.17; 1.23]	0.5%	3.8%
Naficy et al. 2024	462	4.23	0.2577	454	3.78	0.2168	1.12	[1.11; 1.13]	5.4%	3.8%
Huang et al. 2024	136	4.37	0.5160	121	4.93	0.5941	0.89	[0.86; 0.91]	0.4%	3.8%
**Common effect model**	**3585**			**3437**			**1.09**	**[1.08; 1.09]**	**26.4%**	**.**
**Random effects model**							**1.14**	**[1.03; 1.27]**	**.**	**41.4%**
Heterogeneity: I^2 = 99.8%, τ^2 = 0.0309, p = 0										
**Common effect model**	**9721**			**9583**			**0.96**	**[0.96; 0.96]**	**100.0%**	**.**
**Random effects model**							**1.07**	**[0.99; 1.16]**	**.**	**100.0%**

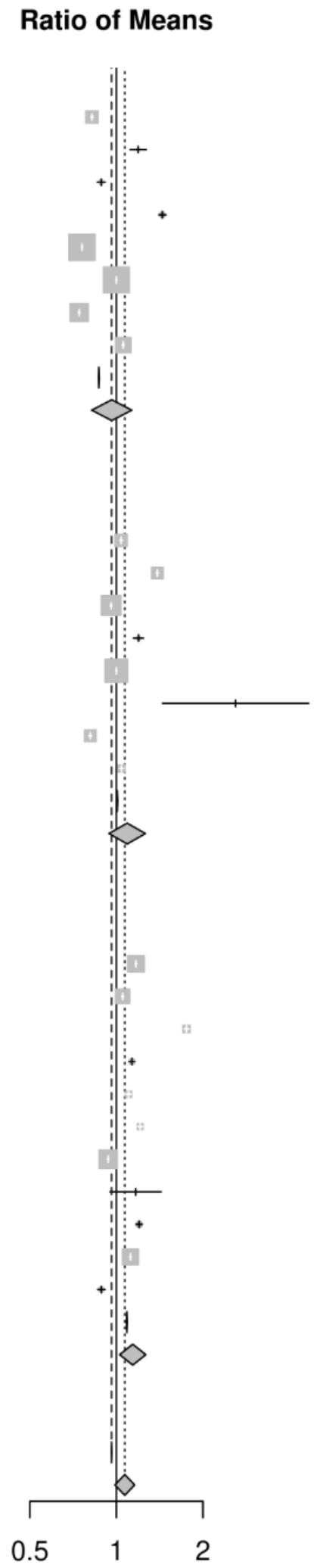

Heterogeneity: I^2 = 99.9%, τ^2 = 0.0423, p = 0
Test for overall effect (random effects): z = 1.67 (p = 0.0952)
Test for subgroup differences (common effect): χ^2_2 = 11111.83, df = 2 (p = 0)
Test for subgroup differences (random effects): χ^2_2 = 2.95, df = 2 (p = 0.2286)

Figure S21: Age subgroup analysis of the Influenza H3N2 Ratio of Means (ROM), comparing geometric mean fold rise (GMFR) between co-administration (CO) and non-concomitant groups (SEQ). Squares represent study-specific estimates, with square size proportional to study weight, and horizontal lines represent 95% confidence intervals. Diamonds represent subgroup and overall estimates. ROM values greater than 1 indicate higher GMFR in the co-administration group.

Figure S22: GMFR COVID-19 - age subgroup analysis

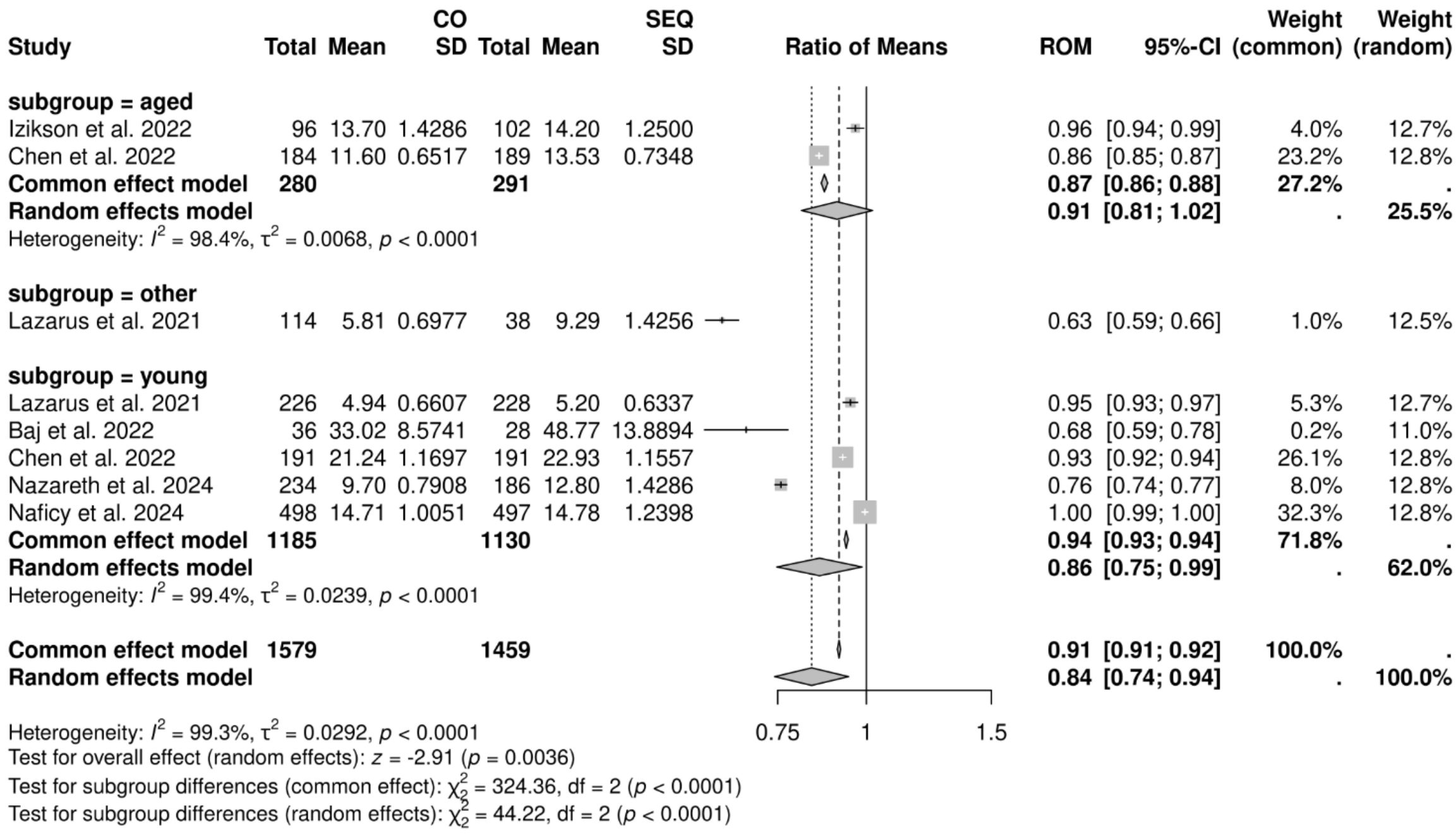

Figure S22: Age subgroup analysis of the COVID-19 Ratio of Means (ROM), comparing geometric mean fold rise (GMFR) between co-administration (CO) and non-concomitant groups (SEQ). Squares represent study-specific estimates, with square size proportional to study weight, and horizontal lines represent 95% confidence intervals. Diamonds represent subgroup and overall estimates. ROM values greater than 1 indicate higher GMFR in the co-administration group.

Sex subgroups

Figure S23: GMFR Influenza H3N2 - sex subgroup analysis

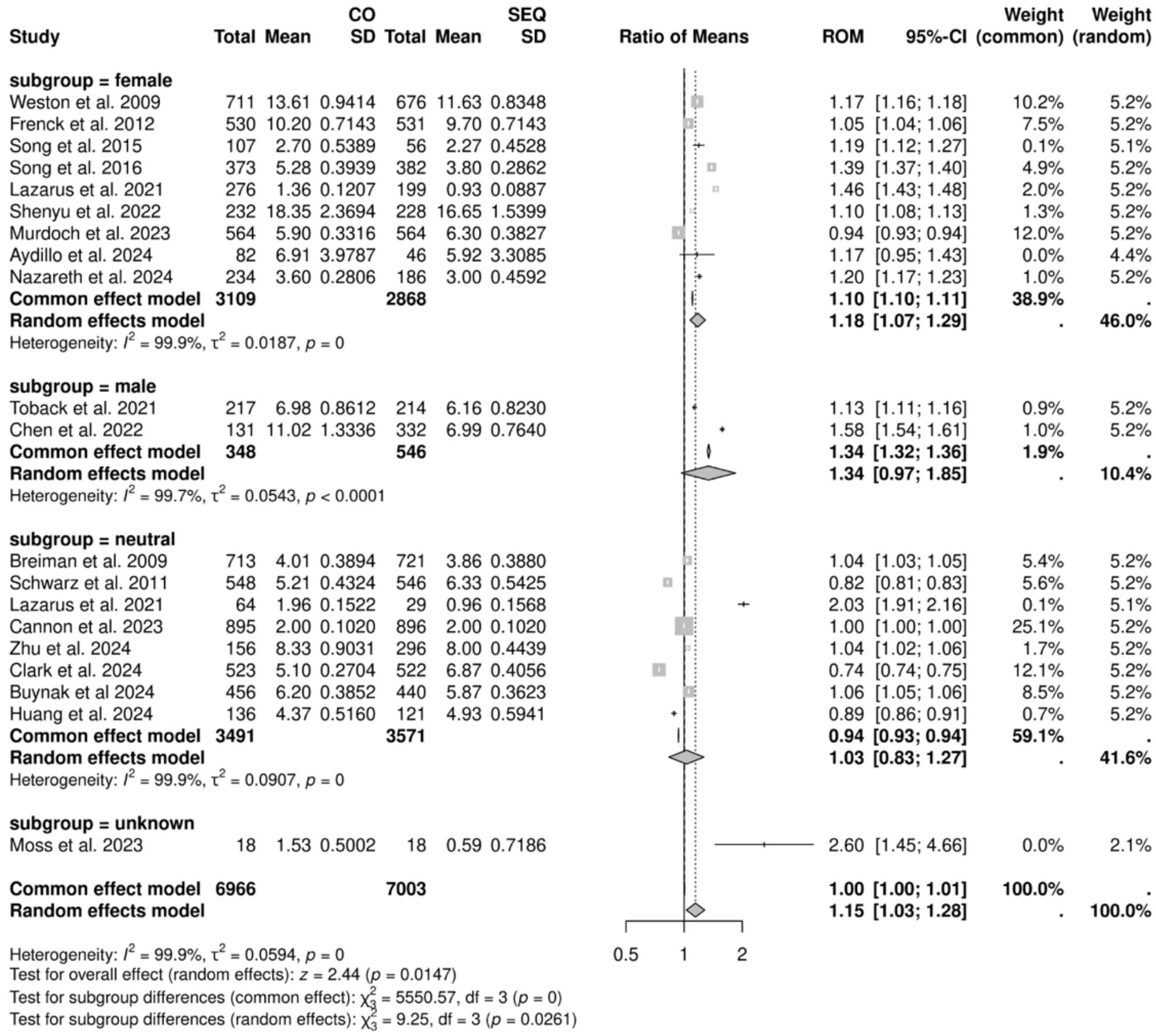

Figure S23: Sex subgroup analysis of the Influenza H3N2 Ratio of Means (ROM), comparing geometric mean fold rise (GMFR) between co-administration (CO) and non-concomitant groups (SEQ). Squares represent study-specific estimates, with square size proportional to study weight, and horizontal lines represent 95% confidence intervals. Diamonds represent subgroup and overall estimates. ROM values greater than 1 indicate higher GMFR in the co-administration group.

Study and vaccine type

Figure S24: GMFR COVID-19 - RCT/non-RCT subgroup analysis

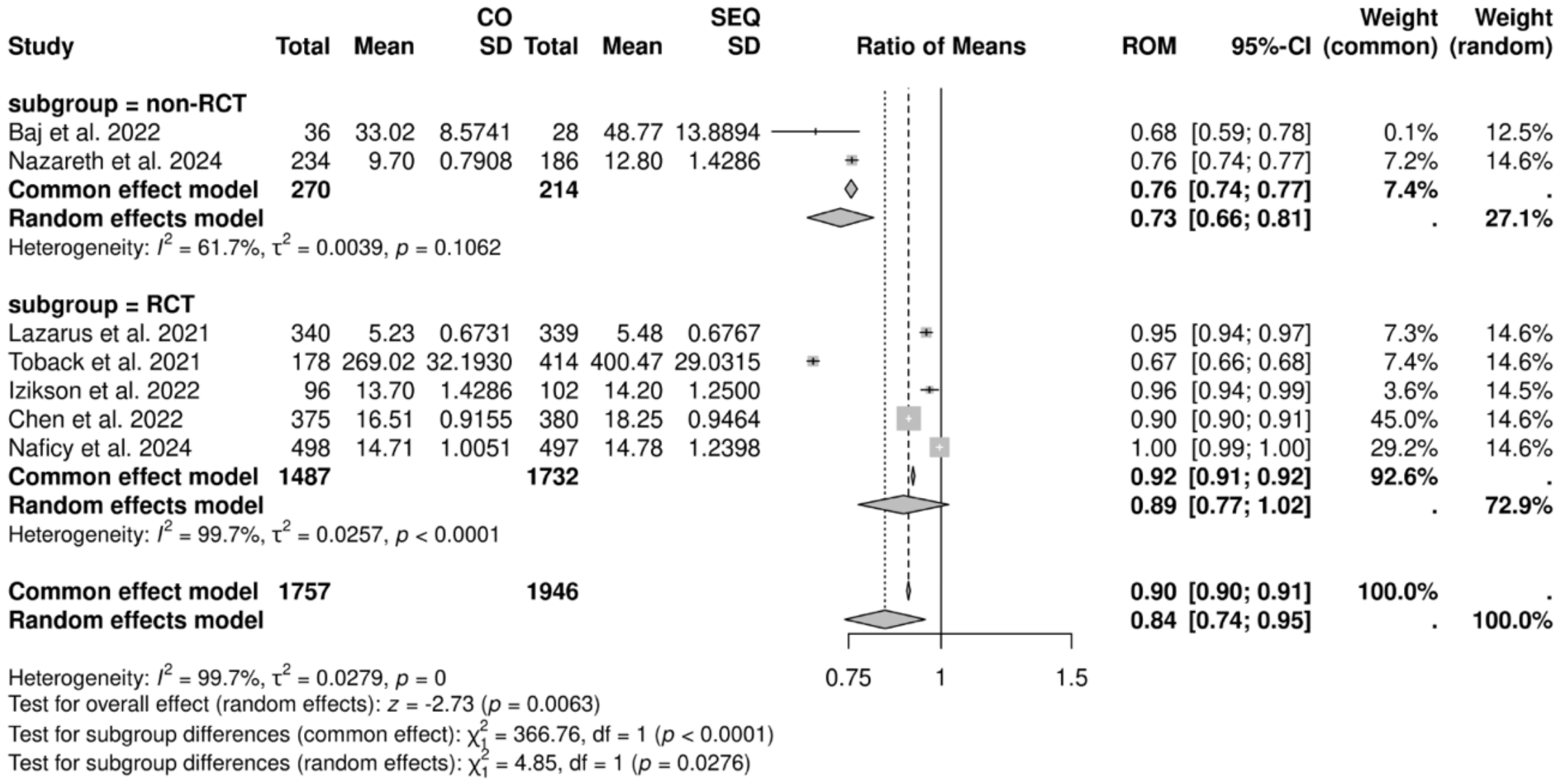

Figure S24: Subgroup analysis by study design of the COVID-19 Ratio of Means (ROM), comparing geometric mean fold rise (GMFR) between co-administration (CO) and non-concomitant groups (SEQ). Squares represent study-specific estimates, with square size proportional to study weight, and horizontal lines represent 95% confidence intervals. Diamonds represent subgroup and overall estimates. ROM values greater than 1 indicate higher GMFR in the co-administration group.

Figure S25: GMFR Influenza H1N1 - cell-based/egg-based

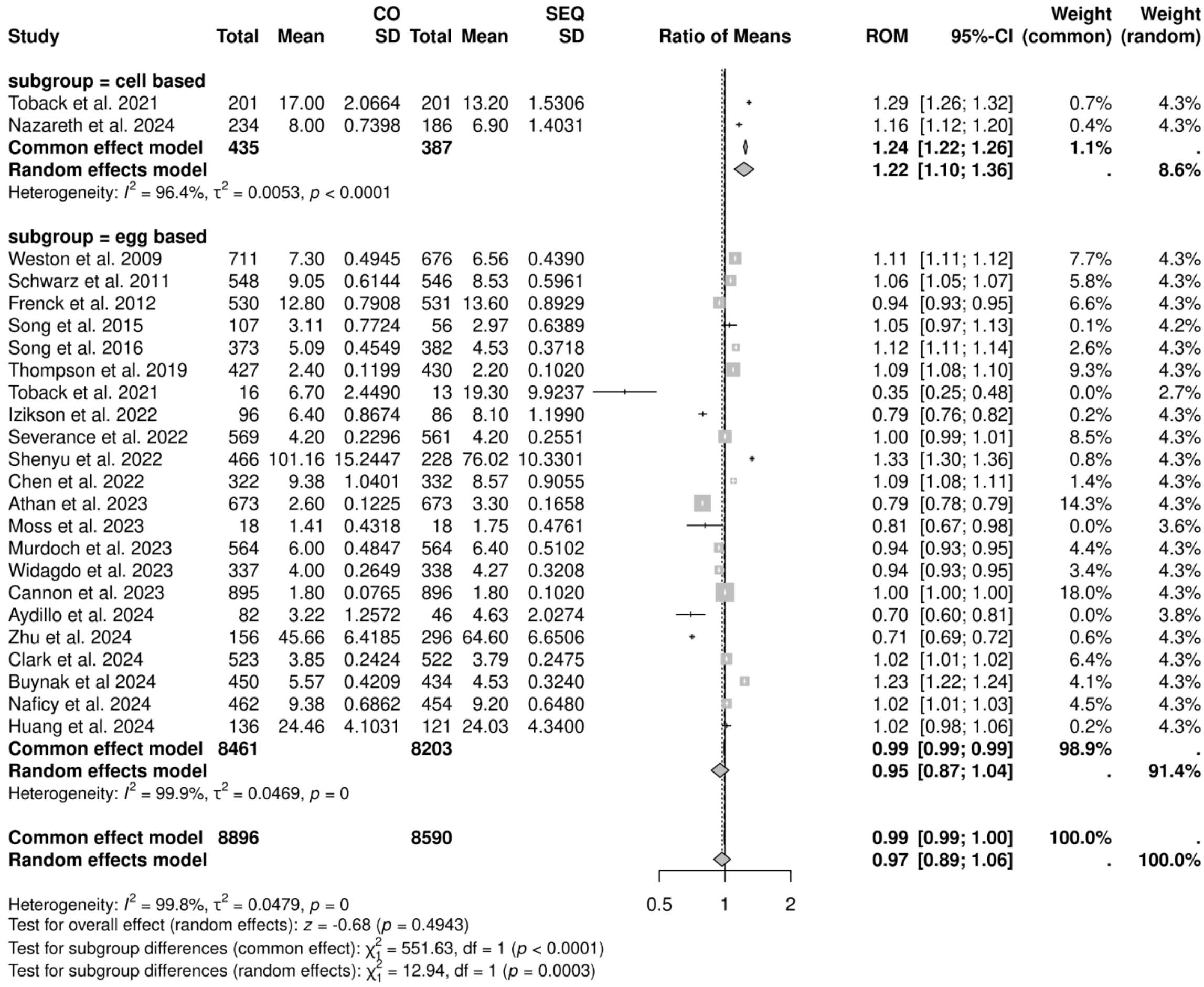

Figure S25: Subgroup analysis by influenza-vaccine platform of the Influenza H1N1 Ratio of Means (ROM), comparing geometric mean fold rise (GMFR) between co-administration (CO) and non-concomitant groups (SEQ). Squares represent study-specific estimates, with square size proportional to study weight, and horizontal lines represent 95% confidence intervals. Diamonds represent subgroup and overall estimates. ROM values greater than 1 indicate higher GMFR in the co-administration group.

Figure S26: GMFR Influenza H3N2 - cell-based/egg-based

Study	CO Total	CO Mean	CO SD	SEQ Total	SEQ Mean	SEQ SD	ROM	95%-CI	Weight (common)	Weight (random)
**subgroup = cell based**										
Toback et al. 2021	201	7.20	0.7653	201	6.10	0.6123	1.18	[1.16; 1.20]	0.8%	4.4%
Nazareth et al. 2024	234	3.60	0.2806	186	3.00	0.4592	1.20	[1.17; 1.23]	0.6%	4.4%
**Common effect model**	**435**			**387**			**1.19**	**[1.17; 1.21]**	**1.4%**	**.**
**Random effects model**							**1.19**	**[1.17; 1.21]**	**.**	**8.9%**
Heterogeneity: I^2 = 5.5%, τ^2 = < 0.0001, p = 0.3037										
**subgroup = egg based**										
Weston et al. 2009	711	13.61	0.9414	676	11.63	0.8348	1.17	[1.16; 1.18]	6.0%	4.5%
Schwarz et al. 2011	548	5.21	0.4324	546	6.33	0.5425	0.82	[0.81; 0.83]	3.3%	4.5%
Frenck et al. 2012	530	10.20	0.7143	531	9.70	0.7143	1.05	[1.04; 1.06]	4.4%	4.5%
Song et al. 2015	107	2.70	0.5389	56	2.27	0.4528	1.19	[1.12; 1.27]	0.1%	4.3%
Song et al. 2016	373	5.28	0.3939	382	3.80	0.2862	1.39	[1.37; 1.40]	2.9%	4.5%
Thompson et al. 2019	427	2.30	0.1071	430	2.40	0.1122	0.96	[0.95; 0.96]	8.5%	4.5%
Toback et al. 2021	16	4.20	2.0664	13	7.10	4.0817	0.59	[0.40; 0.88]	0.0%	2.1%
Izikson et al. 2022	96	3.10	0.3061	86	3.50	0.4082	0.89	[0.86; 0.91]	0.3%	4.4%
Severance et al. 2022	569	2.20	0.1020	561	2.20	0.1020	1.00	[0.99; 1.01]	11.3%	4.5%
Shenyu et al. 2022	466	18.34	2.0124	228	16.65	1.5399	1.10	[1.08; 1.12]	1.4%	4.4%
Chen et al. 2022	322	9.16	1.0247	332	6.99	0.7640	1.31	[1.29; 1.33]	1.2%	4.4%
Athan et al. 2023	673	4.10	0.1811	673	5.40	0.2347	0.76	[0.76; 0.76]	15.1%	4.5%
Moss et al. 2023	18	1.53	0.5002	18	0.59	0.7186	2.60	[1.45; 4.66]	0.0%	1.2%
Murdoch et al. 2023	564	5.90	0.3316	564	6.30	0.3827	0.94	[0.93; 0.94]	7.1%	4.5%
Widagdo et al. 2023	337	2.98	0.2120	338	3.67	0.2578	0.81	[0.80; 0.82]	2.9%	4.5%
Cannon et al. 2023	895	2.00	0.1020	896	2.00	0.1020	1.00	[1.00; 1.00]	14.8%	4.5%
Aydillo et al. 2024	82	6.91	3.9787	46	5.92	3.3085	1.17	[0.95; 1.43]	0.0%	3.4%
Zhu et al. 2024	156	8.33	0.9031	296	8.00	0.4439	1.04	[1.02; 1.06]	1.0%	4.4%
Clark et al. 2024	523	5.10	0.2704	522	6.87	0.4056	0.74	[0.74; 0.75]	7.2%	4.5%
Buynak et al 2024	456	6.20	0.3852	440	5.87	0.3623	1.06	[1.05; 1.06]	5.0%	4.5%
Naficy et al. 2024	462	4.23	0.2577	454	3.78	0.2168	1.12	[1.11; 1.13]	5.6%	4.5%
Huang et al. 2024	136	4.37	0.5160	121	4.93	0.5941	0.89	[0.86; 0.91]	0.4%	4.4%
**Common effect model**	**8467**			**8209**			**0.95**	**[0.95; 0.95]**	**98.6%**	**.**
**Random effects model**							**1.00**	**[0.93; 1.09]**	**.**	**91.1%**
Heterogeneity: I^2 = 99.9%, τ^2 = 0.0355, p = 0										
**Common effect model**	**8902**			**8596**			**0.95**	**[0.95; 0.96]**	**100.0%**	**.**
**Random effects model**							**1.02**	**[0.94; 1.10]**	**.**	**100.0%**

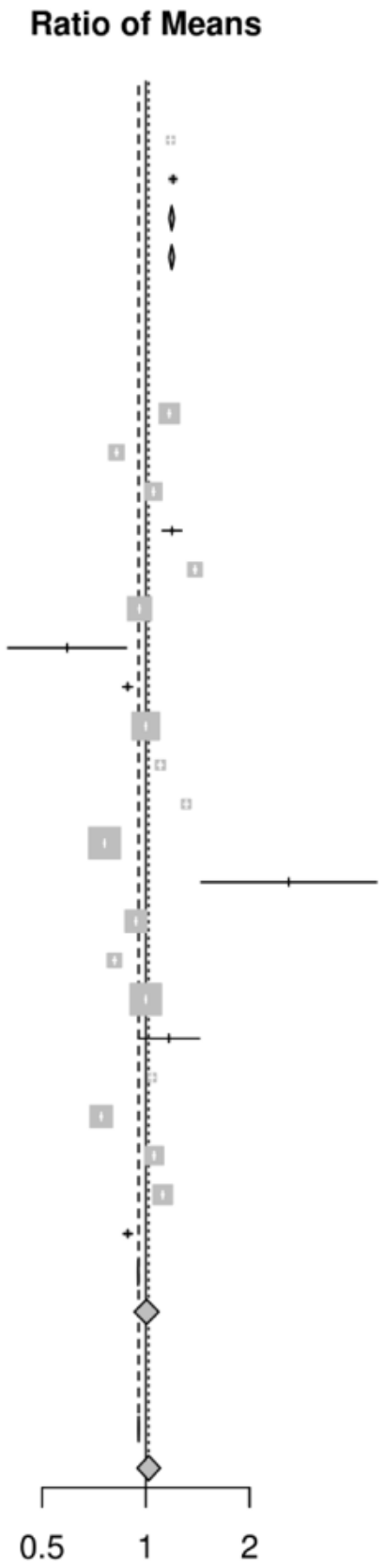

Heterogeneity: I^2 = 99.9%, τ^2 = 0.0346, p = 0
Test for overall effect (random effects): z = 0.50 (p = 0.6156)
Test for subgroup differences (common effect): χ^2_1 = 781.85, df = 1 (p < 0.0001)
Test for subgroup differences (random effects): χ^2_1 = 15.64, df = 1 (p < 0.0001)

Figure S26: Subgroup analysis by influenza-vaccine platform of the Influenza H3N2 Ratio of Means (ROM), comparing geometric mean fold rise (GMFR) between co-administration (CO) and non-concomitant groups (SEQ). Squares represent study-specific estimates, with square size proportional to study weight, and horizontal lines represent 95% confidence intervals. Diamonds represent subgroup and overall estimates. ROM values greater than 1 indicate higher GMFR in the co-administration group.

Figure S27: SPR Influenza H1N1 - Adjuvants vs no adjuvants

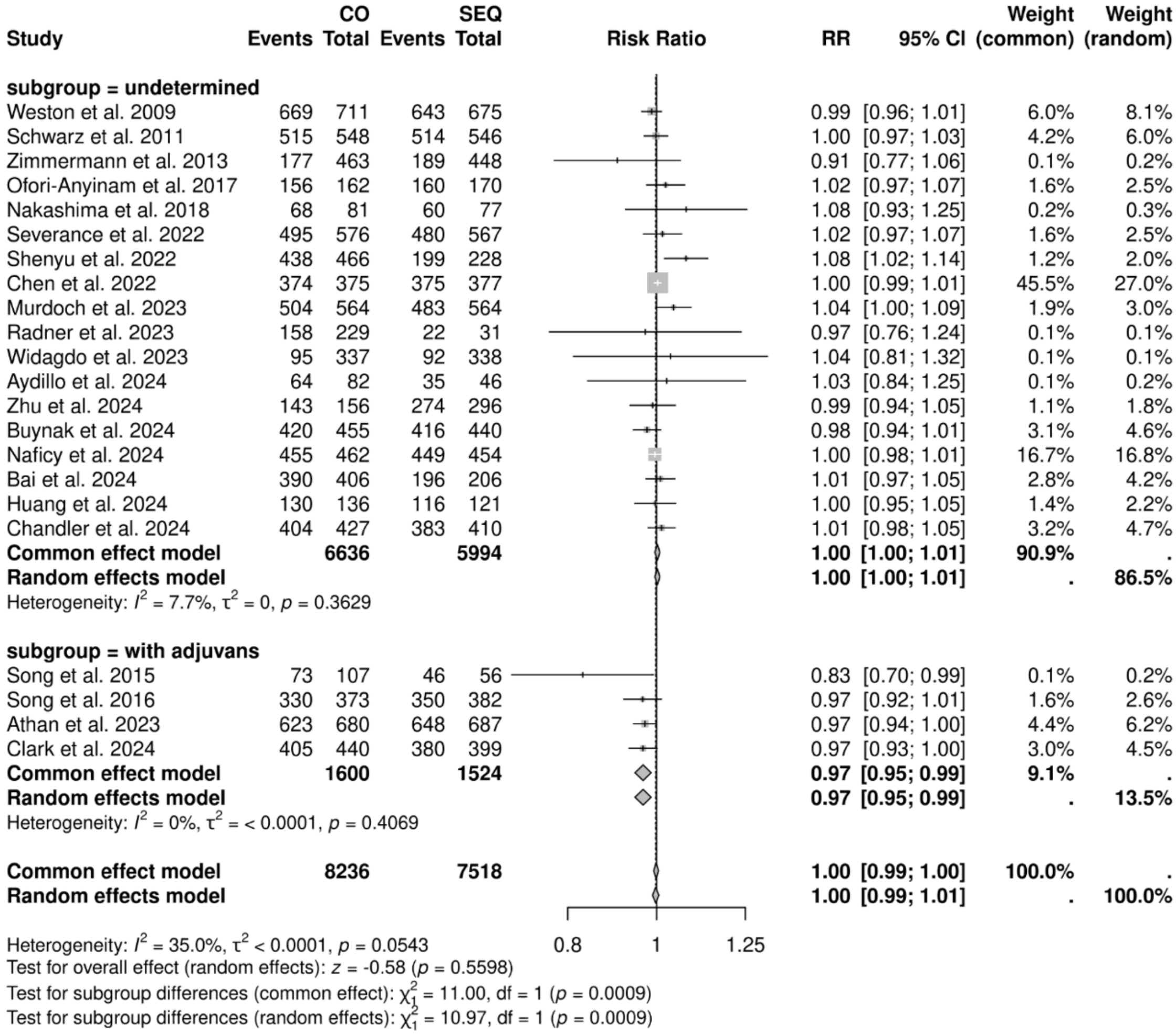

Figure S27: Subgroup analysis by adjuvant use of the Influenza H1N1 risk ratio (RR), comparing seroprotection rates (SPR) between co-administration (CO) and non-concomitant groups (SEQ). Squares represent study-specific estimates, with square size proportional to study weight, and horizontal lines represent 95% confidence intervals. Diamonds represent subgroup and overall estimates. RR values greater than 1 indicate higher seroprotection in the co-administration group.

Figure S28: GMFR Influenza B - north vs. south

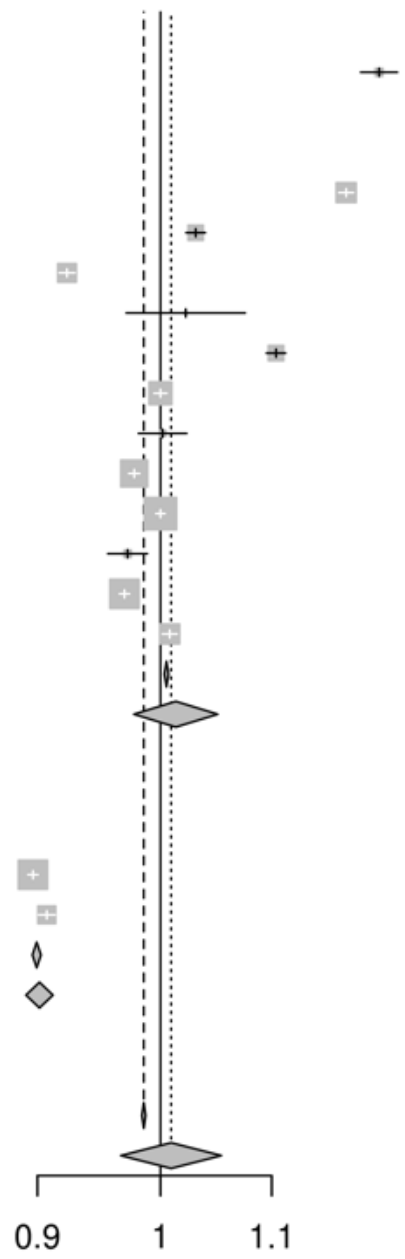

Study	CO Total	CO Mean	CO SD	SEQ Total	SEQ Mean	SEQ SD	ROM	95% CI	Weight (common)	Weight (random)
**subgroup = mixed**										
Nazareth et al. 2024	234	2.05	0.1148	186	1.70	0.1658	1.21	[1.19; 1.23]	1.0%	6.7%
**subgroup = north**										
Weston et al. 2009	711	7.10	0.4250	676	6.05	0.3630	1.17	[1.17; 1.18]	6.5%	6.7%
Schwarz et al. 2011	548	4.05	0.2798	546	3.93	0.2740	1.03	[1.02; 1.04]	3.8%	6.7%
Frenck et al. 2012	530	4.80	0.2551	531	5.20	0.3061	0.92	[0.92; 0.93]	5.7%	6.7%
Song et al. 2015	107	2.07	0.3233	56	2.02	0.3203	1.02	[0.97; 1.08]	0.1%	6.1%
Song et al. 2016	373	2.26	0.1279	382	2.05	0.1195	1.10	[1.10; 1.11]	3.9%	6.7%
Thompson et al. 2019	427	2.15	0.0906	430	2.15	0.0867	1.00	[0.99; 1.01]	8.5%	6.7%
Toback et al. 2021	217	3.74	0.3698	214	3.73	0.4425	1.00	[0.98; 1.02]	0.6%	6.6%
Severance et al. 2022	569	2.20	0.0893	561	2.25	0.0893	0.98	[0.97; 0.98]	11.9%	6.7%
Cannon et al. 2023	895	1.55	0.0510	896	1.55	0.0765	1.00	[1.00; 1.00]	17.2%	6.7%
Zhu et al. 2024	156	6.13	0.5689	296	6.30	0.4796	0.97	[0.96; 0.99]	0.9%	6.7%
Clark et al. 2024	523	1.92	0.0651	522	1.98	0.0727	0.97	[0.97; 0.97]	14.1%	6.7%
Naficy et al. 2024	462	2.54	0.1237	454	2.52	0.1225	1.01	[1.00; 1.01]	6.5%	6.7%
**Common effect model**	**5518**			**5564**			**1.01**	**[1.00; 1.01]**	**79.8%**	**.**
**Random effects model**							**1.01**	**[0.98; 1.05]**	**.**	**79.9%**
Heterogeneity: $I^2 = 99.7\%$, $\tau^2 = 0.0040$, $p = 0$										
**subgroup = south**										
Athan et al. 2023	673	2.60	0.1020	673	2.90	0.1199	0.90	[0.89; 0.90]	14.0%	6.7%
Murdoch et al. 2023	564	4.40	0.2679	564	4.85	0.2934	0.91	[0.90; 0.91]	5.2%	6.7%
**Common effect model**	**1237**			**1237**			**0.90**	**[0.90; 0.90]**	**19.2%**	**.**
**Random effects model**							**0.90**	**[0.89; 0.91]**	**.**	**13.4%**
Heterogeneity: $I^2 = 87.2\%$, $\tau^2 = < 0.0001$, $p = 0.0052$										
**Common effect model**	**6989**			**6987**			**0.99**	**[0.98; 0.99]**	**100.0%**	**.**
**Random effects model**							**1.01**	**[0.97; 1.05]**	**.**	**100.0%**

Heterogeneity: $I^2 = 99.8\%$, $\tau^2 = 0.0072$, $p = 0$
Test for overall effect (random effects): $z = 0.41$ ($p = 0.6788$)
Test for subgroup differences (common effect): $\chi^2_2 = 3459.89$, df = 2 ($p = 0$)
Test for subgroup differences (random effects): $\chi^2_2 = 851.72$, df = 2 ($p < 0.0001$)

Figure S28: Geographic subgroup analysis of the Influenza B Ratio of Means (ROM), comparing geometric mean fold rise (GMFR) between co-administration (CO) and non-concomitant groups (SEQ). Squares represent study-specific estimates, with square size proportional to study weight, and horizontal lines represent 95% confidence intervals. Diamonds represent subgroup and overall estimates. ROM values greater than 1 indicate higher GMFR in the co-administration group.

Table S7: subgroup analyses for Influenza H1N1

Subgroup	Effect size (REML)	95% CI	CO (n)	NC (n)	Heterogenity	N (studies)
**Outcome: GMFR**						
aged	1.00	[0.89 - 1.13]	3423	3356	I^2 = 99.9%, τ^2 = 0.0275	8
young	1.09	[0.95 - 1.26]	3585	3347	I^2 = 99.8%, τ^2 = 0.0565	11
female	1.09	[0.95 - 1.27]	3109	2869	I^2 = 99.8%, τ^2 = 0.0536	9
male	1.06	[0.85 - 1.34]	348	546	I^2 = 99.4%, τ^2 = 0.0272	2
RCT	1.04	[0.96 - 1.13]	9615	9400	I^2 = 99.9%, τ^2 = 0.0343	22
non-RCT	0.88	[0.64 - 1.19]	334	250	I^2 = 96.3%, τ^2 = 0.0696	3
trivalent	0.84	[0.49 - 1.43]	1207	1127	I^2 = 94.2 %, τ^2 = 0.2936	4
quadrivalent	0.98	[0.91 - 1.07]	7689	7463	I^2 = 99.9%, τ^2 = 0.0317	20
Egg based	0.95	[0.87 - 1.04]	8461	8203	I^2 = 99.9%, τ^2 = 0.0469	22
Cell based	1.22	[1.10 - 1.36]	435	387	I^2 = 96.4%, τ^2 = 0.0053	2
adjuvants	0.85	[0.62 - 1.17]	2587	2542	I^2 = 99.9%, τ^2 = 0.1554	6
no-adjuvants	1.01	[0.93 - 1.10]	7022	6769	I^2 = 99.7%, τ^2 = 0.0320	19
north	1.03	[0.94 - 1.12]	7765	7506	I^2 = 99.8%, τ^2 = 0.0399	21
south	0.86	[0.72 - 1.02]	1237	1237	I^2 = 99.9%, τ^2 = 0.0151	2
**Outcome: seroprotection rate**						
aged	0.99	[0.97 - 1.00]	2489	2393	I^2 = 49.9%, τ^2 = 0.0002	7
young	1.00	[0.99 – 1.01]	3013	2514	I^2 = 33.5%, τ^2 = 0	9
female	1.00	[0.96 - 1.05]	2298	1982	I^2 = 66.2%, τ^2 = 0.0022	7
male	1.00	[.99 - 1.01]	630	830	I^2 = 0%, τ^2 = 0	3
RCT	1.00	[0.99 - 1.01]	7925	7441	I^2 = 41%, τ^2 < 0.0001	20
non-RCT	1.00	[0.86 - 1.17]	311	77	I^2 = 0%, τ^2 = 0	2
trivalent	0.97	[0.94 - 1.00]	1654	1561	I^2 = 41.1%, τ^2 = 0.0002	4

quadrivalent	1.00	[1.00 - 1.01]	6582	5957	$I^2 = 28.5\%$, $\tau^2 < 0.0001$	18
Egg based	1.00	[0.99 - 1.01]	8236	7519	$I^2 = 35\%$, $\tau^2 < 0.0001$	22
Cell based	N/A	N/A	N/A	N/A	N/A	N/A
adjuvants	0.97	[0.95 - 0.99]	1600	1524	$I^2 = 0\%$, $\tau^2 < 0.0001$	4
no-adjuvants	1.00	[1.00 - 1.01]	6636	5994	$I^2 = 7.7\%$, $\tau^2 = 0$	18
north	1.00	[0.99 - 1.01]	6565	5857	$I^2 = 26.3\%$, $\tau^2 < 0.0001$	19
south	1.00	[0.94 - 1.08]	1244	1251	$I^2 = 85.8\%$, $\tau^2 = 0.0022$	2

Table S8: subgroup analyses for Influenza H3N2

**Subgroup**	**Effect size (REML)**	**95% CI**	**CO (n)**	**NC (n)**	**Heterogenity**	**N (studies)**
**Outcome: GMFR**						
aged	0.96	[0.82 - 1.13]	3429	3362	$I^2 = 99.9\ \%$, $\tau^2 = 0.0534$	8
young	1.14	[1.03 - 1.27]	3585	3437	$I^2 = 99.8\%$, $\tau^2 = 0.0309$	11
female	1.18	[1.07 - 1.29]	3109	2868	$I^2 = 99.9\%$, $\tau^2 = 0.0187$	9
male	1.34	[0.97 - 1.85]	348	546	$I^2 = 99.7\%$, $\tau^2 = 0.0543$	2
RCT	1.03	[0.95 - 1.11]	9621	9406	$I^2 = 99.9\%$, $\tau^2 = 0.0355$	22
non-RCT	1.41	[0.94 - 2.10]	334	250	$I^2 = 70.5\%$, $\tau^2 = 0.1028$	3
trivalent	1.08	[0.79 - 1.48]	1207	1127	$I^2 = 99.6\%$, $\tau^2 = 0.0922$	4
quadrivalent	1.00	[0.93 - 1.08]	7695	7469	$I^2 = 99.9\%$, $\tau^2 = 0.0283$	20
Egg based	1.00	[0.93 - 1.09]	8467	8209	$I^2 = 99.9\%$, $\tau^2 = 0.0355$	22
Cell based	1.19	[1.17 - 1.21]	435	387	$I^2 = 5.5\%$, $\tau^2 < 0.0001$	2
adjuvants	0.93	[0.73 - 1.18]	2587	2542	$I^2 = 100\%$, $\tau^2 = 0.0878$	6
no-adjuvants	1.04	[0.97 - 1.11]	7028	6775	$I^2 = 99.8\%$, $\tau^2 = 0.0197$	19
north	1.07	[0.98 - 1.16]	7771	7512	$I^2 = 99.9\%$, $\tau^2 = 0.0377$	21

south	0.84	[0.69 - 1.04]	1237	1237	$I^2 = 100\%$, $\tau^2 = 0.0220$	2
**Outcome: seroprotection rate**						
aged	0.98	[0.96 - 1.00]	2495	2393	$I^2 = 53.7\%$, $\tau^2 = 0.0003$	7
young	1.00	[0.99 - 1.00]	3013	2514	$I^2 = 0\%$, $\tau^2 = 0$	9
female	1.00	[0.99 - 1.01]	2298	1982	$I^2 = 0\%$, $\tau^2 = 0$	7
male	1.00	[1.00 - 1.01]	630	830	$I^2 = 0\%$, $\tau^2 < 0.0001$	4
RCT	1.00	[1.00 - 1.00]	7914	7429	$I^2 = 54.1\%$, $\tau^2 = 0$	20
non-RCT	1.08	[0.86 - 1.34]	311	77	$I^2 = 0\%$, $\tau^2 = 0$	2
trivalent	0.95	[0.89 - 1.03]	1654	1561	$I^2 = 81.7\%$, $\tau^2 = 0.0049$	4
quadrivalent	1.00	[1.00 - 1.01]	6571	5945	$I^2 = 27.1\%$, $\tau^2 = 0$	18
Egg based	1.00	[1.00 - 1.00]	8225	7506	$I^2 = 49.9\%$, $\tau^2 = 0$	22
Cell based	N/A	N/A	N/A	N/A	N/A	N/A
adjuvants	0.97	[0.95 - 1.00]	1601	1525	$I^2 = 60.3\%$, $\tau^2 = 0.0005$	4
no-adjuvants	1.00	[1.00 - 1.00]	6624	5981	$I^2 = 46.9\%$, $\tau^2 = 0$	18
north	1.00	[1.00 - 1.00]	6566	5857	$I^2 = 49.5\%$, $\tau^2 = 0$	19
south	1.00	[0.93 - 1.07]	1243	1251	$I^2 = 82.3\%$, $\tau^2 = 0.0021$	2

Table S9: subgroup analyses for Influenza B

Subgroup	Effect size (REML)	95% CI	CO (n)	NC (n)	Heterogenity	N (studies)
**Outcome: GMFR**						
aged	0.98	[0.93 - 1.03]	2746	693	I^2 = 99.8%, τ^2 = 0.0032	5
young	1.03	[0.94 - 1.13]	2718	2625	I^2 = 99.9%, τ^2 = 0.0142	6
female	1.05	[0.95 - 1.16]	2519	2395	I^2 = 99.9%, τ^2 = 0.0146	6
male	N/A	N/A	N/A	N/A	N/A	N/A
RCT	1.00	[0.96 - 1.03]	6755	6801	I^2 = 99.8%, τ^2 = 0.0051	14
non-RCT	N/A	N/A	N/A	N/A	N/A	N/A
trivalent	0.91	[0.61 - 1.34]	1207	1127	I^2 = 98.3%, τ^2 = 0.1548	4
quadrivalent	0.99	[0.95 - 1.04]	5782	5860	I^2 = 99.7%, τ^2 = 0.0061	12
Egg based	0.98	[0.93 - 1.03]	6554	6600	I^2 = 99.8%, τ^2 = 0.0084	14
Cell based	1.13	[0.99 - 1.29]	435	387	I^2 = 99.2%, τ^2 = 0.0089	2
adjuvants	0.91	[0.74 - 1.11]	2587	2542	I^2 = 99.8%, τ^2 = 0.0603	6
no-adjuvants	1.02	[0.96 - 1.08]	4402	4445	I^2 = 99.8%, τ^2 = 0.0085	10
north	1.01	[0.98–1.05]	5518	5564	I^2 = 99.7%, τ^2 = 0.0040	12
south	0.90	[0.89–0.91]	1237	1237	I^2 = 87.2%, τ^2 < 0.0001	2
**Outcome: seroprotection rate**						
aged	1.00	[1.00 - 1.00]	1164	1105	I^2 = 27.4%, τ^2 = 0	4
young	1.02	[0.99 - 1.05]	2302	1839	I^2 = 36%, τ^2 = 0.0005	8
female	1.02	[0.95 - 1.09]	1107	869	I^2 = 36.2%, τ^2 = 0.0020	4
male	0.97	[0.90 - 1.04]	630	830	I^2 = 74.8%, τ^2 = 0.0035	4
RCT	1.00	[1.00 - 1.00]	5047	4648	I^2 = 17.2%, τ^2 = 0	14
non-RCT	1.13	[0.87 - 1.47]	311	77	I^2 = 0%, τ^2 = 0	2
trivalent	N/A	N/A	N/A	N/A	N/A	N/A
quadrivalent	1.00	[1.00 - 1.00]	5358	4725	I^2 = 9.4%, τ^2 = 0	16

Egg based	1.00	[1.00 - 1.00]	5358	4725	I^2 = 9.4%, τ^2 =0	16
Cell based	N/A	N/A	N/A	N/A	N/A	N/A
adjuvants	N/A	N/A	N/A	N/A	N/A	N/A
no-adjuvants	1.00	[1.00 - 1.00]	4916	4325	I^2 = 15.4%, τ^2 = 0	15
north	1.00	[1.00–1.00]	4367	3751	I^2 = 0%, τ^2 = 0	13
south	N/A	N/A	N/A	N/A	N/A	N/A

Table S10: subgroup analyses for COVID-19

Subgroup	Effect size (REML)	95% CI	CO (n)	NC (n)	Heterogenity	N (studies)
**Outcome: GMFR**						
aged	0.91	[0.81 - 1.02]	280	291	I^2 = 98.4%, τ^2 = 0.0068	2
young	0.86	[0.75 - 0.99]	1185	1130	I^2 = 99.4%, τ^2 = 0.0239	5
female	0.83	[0.62 - 1.12]	546	413	I^2 = 99.7%, τ^2 = 0.0669	3
male	N/A	N/A	N/A	N/A	N/A	N/A
RCT	0.89	[0.77 - 1.02]	1487	1732	I^2 = 99.7%, τ^2 = 0.0039	5
non-RCT	0.73	[0.66 - 0.81]	270	214	I^2 = 61.7%, τ^2 = 0.0039	2
north	N/A	N/A	N/A	N/A	N/A	N/A
south	N/A	N/A	N/A	N/A	N/A	N/A

Table S11: Number of studies contributing to AE pooled estimate and zero-event studies excluded

Adverse event	studies contributing to estimate	zero-event studies excluded
Any AE	17	0
Serious AE	26	3
Arthralgia	19	1
Chills	15	0
Fatigue	26	0
Fever	33	1
Headache	30	0
Myalgia	27	0
Cough	6	1
Diarrhea	10	3
Gastrointestinal AE	3	0
Malaise	8	0
Nausea	13	2
Rash	3	1
Vomiting	13	2

Table S12: Eligible seasonal influenza vaccine co-administration combinations and comparator groups[1]

Index vaccine	co-administered vaccine category	Intervention vaccine combination	Eligible comparator/control group
Seasonal Influenza Vaccine	COVID-19 vaccine	Influenza + COVID-19 vaccine	Influenza vaccine only; COVID-19 vaccine only; influenza vaccine + placebo; COVID-19 vaccine + placebo; sequential administration
Seasonal Influenza Vaccine	Pneumococcal vaccine	Influenza + pneumococcal vaccine	Influenza vaccine only; pneumococcal vaccine only; influenza vaccine + placebo; pneumococcal vaccine + placebo; sequential administration
Seasonal Influenza Vaccine	RSV vaccine	Influenza + RSV vaccine	Influenza vaccine only; RSV vaccine only; influenza vaccine + placebo; RSV vaccine + placebo; sequential administration
Seasonal Influenza Vaccine	DTP-containing vaccine	Influenza + DTP-containing vaccine	Influenza vaccine only; DTP-containing vaccine only; sequential administration of both vaccines
Seasonal Influenza Vaccine	Poliovirus vaccine	Influenza + poliovirus vaccine	Influenza vaccine only; polio vaccine only; sequential administration of both vaccines
Seasonal Influenza Vaccine	BCG vaccine	Influenza + BCG vaccine	Influenza vaccine only; BCG vaccine only; influenza vaccine + placebo; BCG vaccine + placebo; sequential administration
Seasonal Influenza Vaccine	MMR vaccine	Influenza + MMR vaccine	Influenza vaccine only; MMR vaccine only; influenza vaccine + placebo; MMR vaccine + placebo; sequential administration

[1] Comparator/control groups listed in this table were eligible according to the outcome assessed. For influenza immunogenicity analyses, comparator groups were required to provide influenza immunogenicity data from a non-concomitant influenza-containing regimen. For safety analyses, all eligible non-concomitant comparator/control regimens were considered because adverse events were analysed at the regimen level.
